# Isatin-tethered halogen-containing acylhydrazone derivatives as monoamine oxidase inhibitor with neuroprotective effect

**DOI:** 10.1038/s41598-024-51728-x

**Published:** 2024-01-13

**Authors:** Sunil Kumar, Jong Min Oh, Prabitha Prabhakaran, Abhimanyu Awasti, Hoon Kim, Bijo Mathew

**Affiliations:** 1 Department of Pharmaceutical Chemistry, Amrita School of Pharmacy, Amrita Vishwa Vidyapeetham, AIMS Health Sciences Campus, Kochi, 682041 India; 2 Department of Pharmacy, and Research Institute of Life Pharmaceutical Sciences, Sunchon National University, Suncheon, 57922 Republic of Korea; 3 Department of Pharmaceutical Chemistry, JSS College of Pharmacy, JSS Academy of Higher Education and Research, Mysuru, 570015 India

**Keywords:** Biochemistry, Biological techniques, Drug discovery

## Abstract

Sixteen isatin-based hydrazone derivatives (**IS1**–**IS16**) were synthesized and assessed for their ability to inhibit monoamine oxidases (MAOs). All the molecules showed improved inhibitory MAO-B activity compared to MAO-A. Compound **IS7** most potently inhibited MAO-B with an IC_50_ value of 0.082 μM, followed by **IS13** and **IS6** (IC_50_ = 0.104 and 0.124 μM, respectively). Compound **IS15** most potently inhibited MAO-A with an IC_50_ value of 1.852 μM, followed by **IS3** (IC_50_ = 2.385 μM). Compound **IS6** had the highest selectivity index (SI) value of 263.80, followed by **IS7** and **IS13** (233.85 and 212.57, respectively). In the kinetic study, the K_i_ values of **IS6**, **IS7**, and **IS13** for MAO-B were 0.068 ± 0.022, 0.044 ± 0.002, and 0.061 ± 0.001 μM, respectively, and that of **IS15** for MAO-A was 1.004 ± 0.171 μM, and the compounds were reversible-type inhibitors. The lead compounds were central nervous system (CNS) permeable, as per parallel artificial membrane permeability assay (PAMPA) test results. The lead compounds were examined for their cytotoxicity and potential neuroprotective benefits in hazardous lipopolysaccharide (LPS)-exposed SH-SY5Y neuroblastoma cells. Pre-treatment with lead compounds enhanced anti-oxidant levels (SOD, CAT, GSH, and GPx) and decreased ROS and pro-inflammatory cytokine (IL-6, TNF-alpha, and NF-kB) production in LPS-intoxicated SH-SY5Y cells. To confirm the promising effects of the compound, molecular docking, dynamics, and MM-GBSA binding energy were used to examine the molecular basis of the **IS7**-MAO-B interaction. Our findings indicate that lead compounds are potential therapeutic agents to treat neurological illnesses, such as Parkinson's disease.

## Introduction

Neurological illnesses are characterized by the progressive and permanent loss of neurons in particular brain areas^1^. Many neurodegenerative disorders are the result of several causes, most likely involving a variety of mechanistic pathways^2^. Monoamine oxidase-B (MAO-B) represents such a pathway and may play a significant role in neurological disorders such as Alzheimer's disease (AD) and Parkinson's disease (PD)^3^. The mitochondrial outer membranes of neurons, glia, and other mammalian cells are closely related to the C-terminal transmembrane polypeptide components of MAOs, also known as flavin adenine dinucleotide (FAD)-carrying enzymes^4,5^. For synaptic connections to function appropriately, xenobiotic and biogenic amine oxidation must be stimulated^6^. The three-dimensional shapes of the MAO isoforms MAO-A and MAO-B share 70% identical amino acid residues. With only a change of six amino acids between the 16 active-site residues of the two MAOs, their active-site geometries are also similar^7–10^. The exact location of MAO isoforms in the brain is not yet fully elucidated. In contrast to studies that employed cell cultures and suggested MAO-A localization in glial cells, tests in both primate and non-primate species demonstrated that the glial enzyme is primarily present as type B in the intact brain^11,12^. MAO-B catalyzes phenyl ethylamine and phenyl methylamine disintegration, whereas MAO-A catalyzes noradrenaline, adrenaline, and serotonin deamination. MAO-A and MAO-B also metabolize dopamine, tryptamine, and tyramine. While MAO-B inhibition increases dopamine levels in the Parkinsonian brain, partially depletes dopaminergic neurons in the substantia nigra pars compacta, and has anti-Parkinsonian effects, selective MAO-A inhibition increases neurotransmitter levels in central nervous system (CNS) noradrenergic and 5-hydroxytryptaminergic neurons^13–15^.

The mechanism-based inhibitors, selegiline and rasagiline (both MAO-B inhibitors) and clorgyline (a MAO-A inhibitor), are among the isoform-specific inhibitors described. Both MAO isoforms are inhibited by pargyline, a different propargylamine molecule. Other notable irreversible MAO inhibitors include the nonspecific inhibitors phenelzine and tranylcypromine^16,17^. The MAO-A inhibitors, toloxatone and moclobemide, and the MAO-B inhibitor, safinamide, are well-known examples of isoform-specific reversible inhibitors^18–20^. Dry mouth, nausea, diarrhea, constipation, drowsiness, sleeplessness, dizziness, and light-headedness are the most frequently reported side effects of the current medications used for treatment. When using a patch, skin irritation may also develop at the patch site. The search for novel MAO-A and MAO-B inhibitors has extensively used a variety of heterocycle families as scaffolds, including pyrazolines, chromones, chalcones, xanthines, benzyloxy, thiazoles, coumarins, and their precursors, isatin congeners, thiazolidiniones, and betacarboline^21–26^. As a result, isatin was identified as an effective MAO inhibitor.

**Figure 1:**
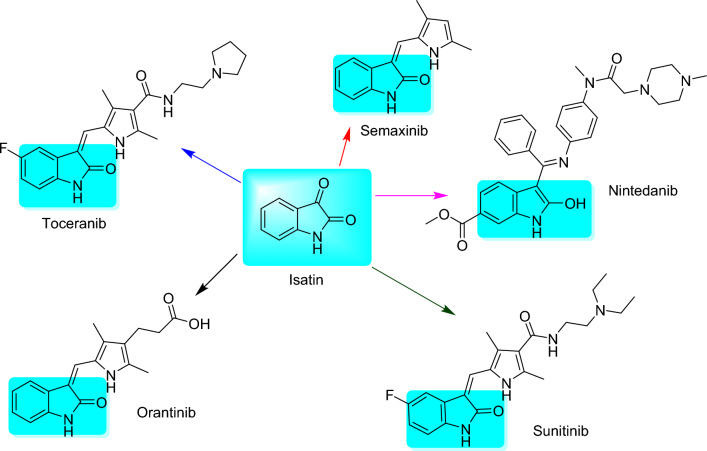
Base structure of isatin and their derivatives for FDA-approved drugs.

Isatin (Fig. 1) is an endogenous small molecule with an indole-containing moiety and exhibits a broad range of biological and pharmacological activities. It comprises a nitrogen atom at position 1 and two carbonyl groups at positions 2 and 3. It further contains two rings: a six-membered aromatic ring and a five-membered antiaromatic ring^27,28^. It is widely distributed in the body fluids and different tissues of mammals and occurs naturally in plants^29^. In addition to clinical studies on the anticancer medications Toceranib, Semaxinib, and Orantinib, the Food and Drug Administration (FDA) has approved isatin-based therapies, such as Sunitinib (anti-tumor) and Nintedanib (anti-tumor)^30–32^ (Fig. 1).

**Figure 2:**
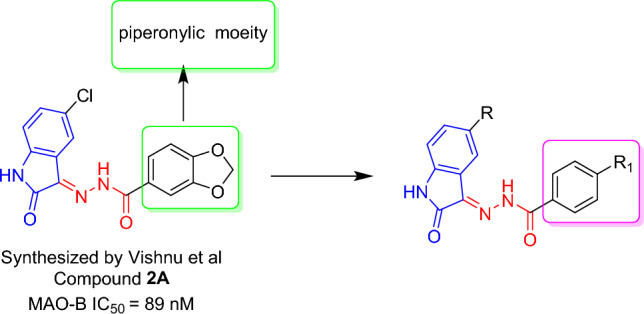
Design strategy of acylhydrazone-based isatin derivatives.

Isatin reversibly inhibits human MAO-A and MAO-B, with K_i_ values of 15 and 3 µM, respectively^33^. According to previous studies, isatin is located close to the FAD cofactor in the MAO-B substrate cavity. The entrance cavity of the enzyme is free as isatin binds to its substrate cavity^34,35^. We hypothesized that the C-3 position could be exploited with hydrophobic moieties to improve MAO efficacy^26^. Therefore, we selected the C-3 position and replaced it with an acyl hydrazone linker and a halogenated phenyl (hydrophobic) moiety. The structural cores of acyl hydrazones, which include two distinctly connected nitrogen atoms, are generally responsible for the physical and chemical properties of these compounds. Therefore, acyl hydrazones are frequently used to develop novel molecules with various functions. Hydrazone derivatives have also been linked to MAO inhibition. Recently, Vishnu et al. synthesized piperonylic hydrazone-based isatin derivatives, however, insignificant interactions were observed in 3,4-methylenedioxy groups with MAO-B binding pocket^36^. Therefore, we replaced piperonylic with phenyl moiety and designed new approach toward acylhydrazone-based isatin derivatives (Fig. 2) to get a new family of effective MAO inhibitors in this study.

## Materials and methods

### Chemicals

For synthesis, isatin derivatives, hydrazine hydrate, and benzoic acid were purchased from Sigma-Aldrich (St. Louis, MO, USA) and TCI Chemical (Toshima, Tokyo, Japan). Substrates (benzylamine and kynuramine) and reference inhibitors (clorgyline, lazabemide, pargyline, and toloxatone) as well as recombinant human MAO-A and MAO-B were purchased from Sigma-Aldrich. Reversibility test was performed by using Dialyzer DiaEasy™ (6–8 kDa, BioVision, St. Grove, MA, USA).

### Synthesis

**Scheme 1:**
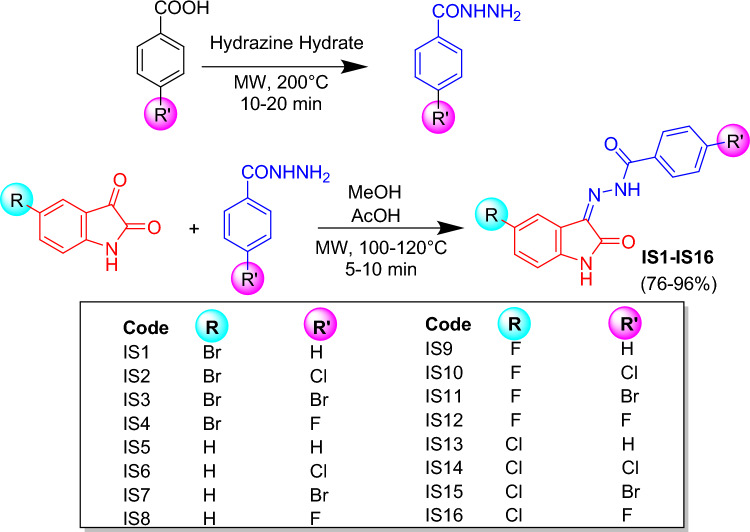
Synthetic protocol of IS1-IS16.

Benzoic acid (1 eq.) and hydrazine hydrate (2.5 eq.) were combined, and the reaction was conducted by using a microwave synthesizer (Monowave 50 Synthesizer, Anton-Paar, Graz, Austria) at 200 °C for 10–20 min. Upon completion of the reaction, the product benzohydrazide was recrystallized from methanol. Then, the mixture of isatin or substituted isatin (0.001 mol) and benzohydrazide (0.001 mol)^37,38^ in methanol, by adding a catalytic amount of acetic acid, was placed in a reaction vial and subjected to the microwave synthesizer at 100–120 °C for 5–10 min. The reaction progress was monitored by using thin-layer chromatography (TLC) with an eluent of ethyl acetate and hexane (50:50). Cold ethanol was used to wash the reaction mixture upon completion, and the resulting product was dried to obtain acylhydrazone-based isatin derivatives (76–96% yield). The synthetic scheme of the isatin derivatives is illustrated in Scheme 1.

### MAO-A and MAO-B inhibition studies

MAO-A and MAO-B activities were determined using 0.06 mM kynuramine and 0.3 mM benzylamine, respectively^39^. In preliminary kinetic study, the K_m_ values of MAO-A and MAO-B were about 0.039 and 0.20 mM, respectively. Concentrations of the substrates used were around 2.0 times of their K_m_ values for the enzyme assay. The absorbance was measured using the continuous assay method described previously^40^. Compound inhibitions were compared to those of the reference inhibitors of MAO-A (toloxatone and clorgyline) and MAO-B (lazabemide and pargyline).

### Enzyme kinetics

The compounds IC_50_ values were calculated using the GraphPad Prism software 5^41^. The selectivity index (SI) values of the compounds were calculated as follows: (IC_50_ of MAO-A)/(IC_50_ of MAO-B)^42^. The type of enzyme inhibition was determined at five different substrate concentrations (0.0075–0.12 μM of MAO-A and 0.0375–0.6 μM of MAO-B). The inhibitor was used at three concentrations (approximately 0.5, 1.0, and 1.5) times its IC_50_ value^41^. Enzyme inhibition patterns and K_i_ values were determined by comparing Lineweaver–Burk (LB) plots and their secondary plots, respectively^40^.

### Reversibility studies

The lead compounds reversibility for MAO-A and MAO-B inhibition was evaluated by comparing the undialyzed and dialyzed values at a concentration of 1.5 × the IC_50_ value after incubation for 30 min prior to measurement, as previously described^39,43^. The restored activities of the compounds were compared to those of the reference compounds toloxatone and clorgyline (reversible and irreversible inhibitors, respectively) of MAO-A, and lazabemide and pargyline (reversible and irreversible inhibitors, respectively) of MAO-B. Reversibility patterns were determined by comparing the activities of undialyzed (A_U_) and dialyzed (A_D_) compounds^41,43^.

### Parallel artificial membrane permeability assay (PAMPA)

The blood–brain barrier (BBB) permeation abilities of the four lead molecules were analyzed using the PAMPA method^44,45^. The detailed procedure is explained in the Supplementary Data.

### Cytotoxicity, reactive oxygen species (ROS), anti-oxidant, and anti-inflammatory characteristics of the cell line-based assay

#### Cell culture and treatments

The National Center for Cell Science (NCCS), Pune, India, supplied the SH-SY5Y-human bone marrow neuroblastoma cell line, which was maintained in accordance with the recommended protocol in DMEM-high-glucose medium (Cat No. AL111, Himedia) supplemented with 10% fetal bovine serum (FBS) and a 1% antibiotic–antimycotic solution at 37 °C in a CO_2_ incubator. Subcultures were performed every two days. Briefly, 5 × 10^5^ cells/mL were cultured on a plate and incubated for 24 h to promote cell attachment and reach the required cell density. Neuroinflammation was induced in the SH-SY5Y cells with 1 ug/mL for 2 h followed by cell treatment with different concentrations of the test molecules (**IS6**, **IS7**, **IS13**, and **IS15**) and incubation for 24 h. LPS-treated cells served as positive controls, and untreated cells served as controls.

#### 3-(4,5-Dimethylthiazol-2-yl)-2,5-diphenyltetrazolium bromide (MTT) assay

The cytotoxicity of synthetic compounds **IS6, IS7,** and **IS13** was assessed using the common MTT test using SHSY-5Y cells. The cells were grown in 96-well plates with 20,000 cells per well. Different compound concentrations were applied to the cells, and the treated cells were then left to incubate for 24 h. After applying 50 µL of MTT (0.5 mg/mL) to each well, the plates were incubated for 3 h, then 100 µL of DMSO was added to dissolve the purple formazan crystal, and an absorbance was measured at 570 nm using a microplate reader (ELX-800, BioTek, CA, USA). Compounds' growth inhibitory concentration (IC_50_) values were computed.

#### Superoxide dismutase (SOD) activity

Using a superoxide dismutase assay kit (KrishGen Biosystems, India), which measures the concentration of formazan crystals using a colorimetric assay, SOD activity was determined. In this test, a tetrazolium salt was used to detect the superoxide radicals produced by xanthine oxidase and hypoxanthine. After treatment, the medium was removed, centrifuged at 2000 rpm for 5 min at room temperature, and placed on ice. A diluted radical detector and lysed cell supernatant or a standard were applied to each well of a 96-well plate to evaluate SOD activity using an ELISA kit, according to the manufacturer’s instructions. The absorbance of the wells was then determined after 5 min at a wavelength of 450 nm using a microplate reader (Safire2, Tecan Group Ltd., Maennedorf, Switzerland). The results were displayed as ng/mL^46–49^.

#### Glutathione (GSH) activity

A glutathione assay kit (KrishGen Biosystems) was used to determine the GSH levels. The assay kit was based on the disulfide dimer-oxidized GSH reductase recycling method for 5,5'-dithiobis-2-(nitrobenzoic acid) (DTNB). After treatment, adherent cells were removed by scraping the media from the wells. Following suspension in 50 mM phosphate solution (0.5 mL) with a pH of 6.5 and 1 mM ethylenediaminetetraacetic acid, the cells were chilled. The lysed cell supernatant was used to test GSH levels using an ELISA kit. The absorbance of the yellow product was determined at a wavelength of 450 nm. The total GSH activity was estimated using a GSH standard curve. The results were obtained as ng/mL^46–49^.

#### Glutathione peroxidase (GPx) activity

A GPx assay kit (KrishGen Biosystems) was used to evaluate the GPx activity. The kit uses a colorimetric assay to determine the quantity of GPx. Glutathione reductase (GR) mediates GPx activity Oxidized glutathione (GSSG) is produced via hydroperoxide reduction by GPx. This glutathione is recycled back to its reduced state by GR and NADPH. The NADPH to NADP + oxidation was accompanied by a decrease in absorbance at 450 nm (A450). When GPx activity was rate-limiting, the rate of decline in A450 was directly correlated with GPx activity. Following treatment, adherent cells were removed from the wells, suspended in cold PBS, sonicated, and frozen. In accordance with the ELISA kit’s instructions, the lysed cell supernatant, or standard, was applied to all 96 wells of a plate together with a diluted radical detector to assay the activity of GPx. A microplate reader was used to measure the absorbance of the wells after 5 min^46–49^. Results were obtained in ng/mL.

#### Catalase (CAT) activity

CAT activity was determined according to Aebi^50^. A human CAT ELISA kit was purchased commercially (KrishGen Biosystems). Following treatment, the adherent cells were scraped off, suspended in cold PBS, sonicated, and placed on ice. The medium was then removed from each well. The 3 mL CAT assay combination comprised of extract (0.05 mL), phosphate buffer (1.5 mL, 100 mM buffer, pH 7.0), H_2_O_2_ (0.5 mL), and distilled water (0.95 mL). The absorbance decreased at 450 nm. CAT activity was reported in terms of ng/mL of H_2_O_2_ oxidized per min per gram^46–49^.

#### ROS assays

The OxiSelect Intracellular ROS Assay Kit (Cell Biolabs Inc., San Diego, CA, USA) was used to quantify the levels of the fluorescent probe 20,70-dichlorodihydrofluorescin diacetate (DCFH-DA). In a microplate reader, fluorescence was measured using excitation and emission filters at wavelengths of 488 and 535 nm, respectively^51^.

#### IL-6, TNF-α, and NF-kB expression

TNF-α, IL-6, and NF-kB expression in cell lysates was assessed using the respective antibodies (PerCP-Cy5.5, PE, and p65–FITC) according to the manufacturer’s protocol. Briefly, the spent medium was aspirated, and the cells were treated with LPS (1 µg/mL) for 2 h. Then, the required concentrations of experimental compounds and controls were added and incubated for 24 h. The cells were harvested into polystyrene tubes and centrifuged at 25 °C, washed with PBS, and 70% cold ethanol was added drop wise to create a cell pellet while vortexing. The mixture was then incubated at − 20 °C. The cells were pelleted at a high speed, washed twice with PBS, antibodies added (10 µL), mixed thoroughly, and incubated for 30 min in the dark at 20–25 °C. PBS (500 µL) was added and mixed thoroughly, and the reaction was analyzed using BD FACS—Cell Quest pro software^46–49,52^.

### Statistical analysis

Statistical significance was determined by one-way ANOVA followed by Dunnett-t test using Graph Pad Prism Version 8.0.2.

### Computational studies

#### Molecular docking

The Schrödinger suite^53^ was used to perform the molecular docking investigation of **IS3**, **IS6**, **IS7** and **IS13**, and **IS15**. The human MAO-A (hMAO-A, 2Z5Z) and MAO-B (hMAO-B, 2V5Z) X-ray solved structure was obtained from the Protein Data Bank^9,54^. The both crystal structures were improved and optimized using the protein preparation wizard included in the Schrödinger suite, which performed energy minimization, hydrogen atom addition, protonation-state correction, and protonation-state addition. The LigPrep tool was used to construct the ligand structures. The co-crystallized ligands served as the automated center of the grid box. For docking simulations, the force Field OPLS4 default settings and extra precision (XP) docking protocol default settings were used^55,56^.

#### Molecular dynamic simulation

Schrödinger LLC’s Desmond simulation program was used to run the molecular dynamics (MD) simulations^52^. The protein–ligand combination was initially created for the Desmond system builder panel using compound **IS7** against MAO-B in an aqueous solvent system. For complete protein–ligand simulations and stability trajectory analysis (RMSD, RMSF, and protein–ligand contact), the simulation parameters were 100 ns at 300 K, 1.01325 bar pressure, and 1000 frames^55,56^.

#### MM-GBSA

The Generalized Born and Surface Area (MM-GBSA) solvation technique in Molecular Mechanics was utilized to compute the free binding energies of the ligands to the proteins. In this case, we used several postures from MD simulations of the docked complex to evaluate macromolecular stability and protein–ligand binding affinity. The free energy was calculated using the following formula at the post-processing stage that comes after the MD studies.

$${\text{G}} = {\text{E}}_{{{\text{int}}}} + {\text{E}}_{{{\text{ele}}}} + {\text{E}}_{{{\text{vdw}}}} + {\text{G}}_{{{\text{pol}}}} + {\text{G}}_{{{\text{np}}}} - {\text{TS}}$$

The contributions of the internal, electrostatic, and van der Waals energies to molecular mechanics are denoted by the symbols E_int_, E_ele_, and E_vdw_, respectively. Within the equation, the free energy contributions of the polar and non-polar solvation systems are denoted by G_pol_ and G_np_, respectively. S is an estimate of the entropy, and T is the absolute temperature. The following formula was used to estimate the binding free energy, or ΔG Bind, between the ligand and the protein.

$$\Delta {\text{G}}\;{\text{Bind}} = \left( {{\text{G}}_{{{\text{PL}}}} } \right) - \left( {{\text{G}}_{{\text{P}}} } \right) - \left( {{\text{G}}_{{\text{L}}} } \right)$$

The protein, ligand, and protein–ligand complex are denoted by the letters P, L, and PL, respectively. The equation above expressed the free energy for each of these entities. We used the solvated systems that we obtained prior to performing MD calculations to determine free binding energies. In this case, solvent molecules more than 5 Å away from the bound ligand were replaced with an implicit model via the GB approach, in their post-processing stages^57,58^.

## Results and discussion

### Synthesis

The target molecules were synthesized in two steps. In the first step, an intermediate *acylhydrazide* molecule was synthesized by reacting benzoic acid with hydrazine hydrate. This intermediate was then reacted with isatin and halogenated substituted isatins to obtain the final molecules (substituted acylhydrazone-based isatin derivatives: (**IS1**–**IS16**) via an acid-catalyzed nucleophilic addition reaction. All the procedures were performed using the microwave reactor. The structures of all synthesized compounds were confirmed by ^1^H and ^13^C nuclear magnetic resonance ((Bruker Advance Neo 400 MHz NMR spectrometer). The de-shielded protons in all compounds were NH atoms from isatin, and the hydrazone linker exhibited ranges of 11.5–11.0 δ and 12.50–14.0 δ, respectively. Sharp de-shielded Sp^2^ carbonyl carbons of the isatin and hydrazone linkers were observed at 163.60 δ and 141.10 δ, respectively (Supporting Information Figs. S1–S48).

### MAO-A and MAO-B inhibition studies

Of the 16 compounds, **IS7** most potently inhibited MAO-B with an IC_50_ value of 0.082 μM, followed by **IS13** (IC_50_ = 0.104 μM) (Table 1, Fig. S52). Compounds **IS7** and **IS6** (*para*-Br and –Cl in the B-ring, respectively) showed higher MAO-B inhibition than the basic compound **IS5** (–H in B-ring, IC_50_ = 4.136 μM), i.e., –Br > –Cl > –H > –F in order. In contrast, MAO-B inhibition decreased in the order of *meta*-position substitution in the A-ring, that is, **IS5** (–H) > **IS13** (–Cl) > **IS1** (–Br) > **IS9** (–F), suggesting that the *meta*-F substituent of the A-ring contributed to a decrease in MAO-B inhibition.

**Table 1:** Monoamine oxidase (MAO)-A and MAO-B inhibition by the 16 compounds of the IS series^a^.

Compound	Residual activity at 10 µM (%)		IC_50_ (µM)		SI^b^
	MAO-A	MAO-B	MAO-A	MAO-B	
IS1	83.08 ± 2.34	22.00 ± 2.75	> 40	0.420 ± 0.028	> 95.24
IS2	46.93 ± 2.73	1.18 ± 2.04	9.155 ± 0.324	0.269 ± 0.004	34.03
IS3	29.53 ± 0.34	1.04 ± 0.23	2.385 ± 0.018	0.514 ± 0.023	4.64
IS4	70.07 ± 1.93	42.46 ± 8.93	32.849 ± 0.134	7.284 ± 1.724	4.51
IS5	75.75 ± 7.44	38.20 ± 4.61	> 40	4.136 ± 0.068	> 9.67
IS6	62.04 ± 1.87	8.53 ± 1.71	32.711 ± 0.210	0.124 ± 0.015	263.80
IS7	50.76 ± 7.29	3.20 ± 0.77	19.176 ± 5.960	0.082 ± 0.010	233.85
IS8	69.65 ± 3.22	67.38 ± 7.80	35.001 ± 0.000	25.473 ± 2.034	> 1.37
IS9	67.38 ± 1.00	45.56 ± 6.28	> 40	9.094 ± 1.281	4.40
IS10	54.06 ± 0.78	42.64 ± 0.82	18.308 ± 0.729	3.995 ± 0.472	4.58
IS11	43.56 ± 0.42	53.24 ± 2.60	13.718 ± 0.396	10.586 ± 0.256	1.30
IS12	36.59 ± 1.60	44.29 ± 4.48	4.288 ± 0.552	7.099 ± 0.956	0.60
IS13	62.28 ± 0.30	5.66 ± 1.75	22.107 ± 0.063	0.104 ± 0.005	212.57
IS14	53.15 ± 0.91	23.00 ± 2.48	11.268 ± 0.414	0.234 ± 0.008	48.15
IS15	34.67 ± 0.71	12.47 ± 3.64	1.852 ± 0.085	0.337 ± 0.007	5.50
IS16	43.66 ± 1.65	26.10 ± 4.39	8.455 ± 0.395	3.848 ± 0.392	2.20
Toloxatone			1.080 ± 0.025	–	
Lazabemide			–	0.110 ± 0.016	
Clorgyline			0.007 ± 0.0007	–	
Pargyline			–	0.140 ± 0.0059	

^a^Results are the means ± standard errors of duplicate or triplicate experiments.

^b^Selectivity index (SI) values are expressed for MAO-B compared to MAO-A.

These IC_50_ values were lower than those of the aldoxime- and hydroxy-functionalized chalcones **ACE7** and **HC6** (IC_50_ = 0.012 and 0.0046 μM, respectively)^59^, but higher than those of the dimethoxy-halogenated chalcone **DM2** (IC_50_ = 0.067 μM)^60^. In contrast, compound **IS15** most inhibited MAO-A with an IC_50_ value of 1.852 μM, followed by **IS3** (IC_50_ = 2.385 μM). These values are more efficient than those of the halogenated pyrazoline **EH8** (IC_50_ = 4.31 μM). Compound **IS6** had the highest selectivity index (SI) value (263.8); however, compounds **IS7** and **IS13** showed similar SI values (SI = 233.85 and 212.57, respectively) and high MAO-B inhibition. These SI values indicated that compounds **IS6**, **IS7,** and **IS13** are selective MAO-B inhibitors (Table 1).

**Figure 3:**
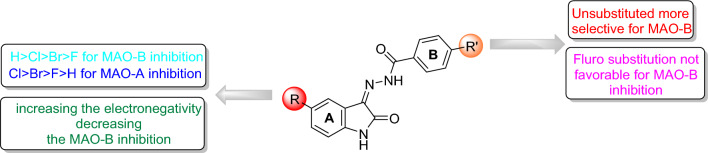
Structure–activity relationship of acylhydrazone-based isatin derivatives.

Structurally, compound **IS7** (–Br in the B-ring) showed higher MAO-B inhibition than **IS6** (–Cl in the B-ring), and both compounds showed 50.4 × and 33.4 × , higher inhibition than the basic compound **IS5** (–H in the B-ring), respectively. In the subseries, MAO-B inhibition increased in the following order: Br > Cl > H > F at the *para*-position of the B-ring. In contrast, in the sub-series containing –Br in the A-ring, **IS2** (–Cl in the B-ring, IC_50_ = 0.269 μM) showed a higher MAO-B inhibition than the sub-parental compound **IS1** (–H in B-ring, IC_50_ = 0.420 μM), and the inhibition increased with the substituents of –Cl > –H > –Br > –F at *para*-position in the B-ring in order). In the other sub-series containing –F in the A ring, **IS10** (–Cl in B-ring, IC_50_ = 3.995 μM) showed higher MAO-B inhibition than the sub-parental compound **IS9** (–H in B), and MAO-B inhibition increased with the substituents of –Cl > –F > –H > –Br at *para*-position in B-ring in order. In the sub-series containing –Cl in the A-ring, **IS13** (–H in the B-ring, IC_50_ = 0.104 μM) showed the highest MAO-B inhibition, which increased with the substituents of –H > –Cl > –Br > –F at *para*-position in the B-ring. In comparing substituents in A ring, MAO-B inhibition increased in order by –Cl (**IS13**, IC_50_ = 0.104 μM) > –Br (**IS1**, IC_50_ = 0.420 μM) > –H (**IS5**, 4.136 μM) > –F (**IS9**, 9.094 μM), and by –H (**IS7**, 0.082 μM) > –Cl (**IS15**, 0.337 μM) > –Br (**IS3**, 0.514 μM) > –F (**IS11**, 10.586 μM). Overall, most compounds with F substituents showed low MAO-B inhibition (Table 1, Fig. 3). **IS7**, **IS6**, and **IS13** were more selective (SI = 233.85, 263.80, and 212.57, respectively) towards MAO-B. The lead molecules (**IS7**, **IS6**, and **IS13**) were comparable to lazabemide and pargyline.

In MAO-A inhibition, compound **IS15** (–Cl in the A-ring and –Br in the B-ring) was the highest (IC_50_ = 1.852 μM) (Table 1, Fig. S53) and showed 11.94-times higher MAO-A inhibition than **IS13** (–Cl in the A-ring and –H in the B-ring), and 10.35-times higher than **IS7** (–H in the A-ring and –Br in the B-ring). This indicates that the –Cl substituent in the A-ring contributed to an increase in MAO-A inhibition (Table 1, Fig. 3). These results suggest that compounds **IS6**, **IS7**, and **IS13** are potent selective MAO-B inhibitors and that compound **IS15** is a selective MAO-A inhibitor.

#### Reversibility studies

**Figure 4:**
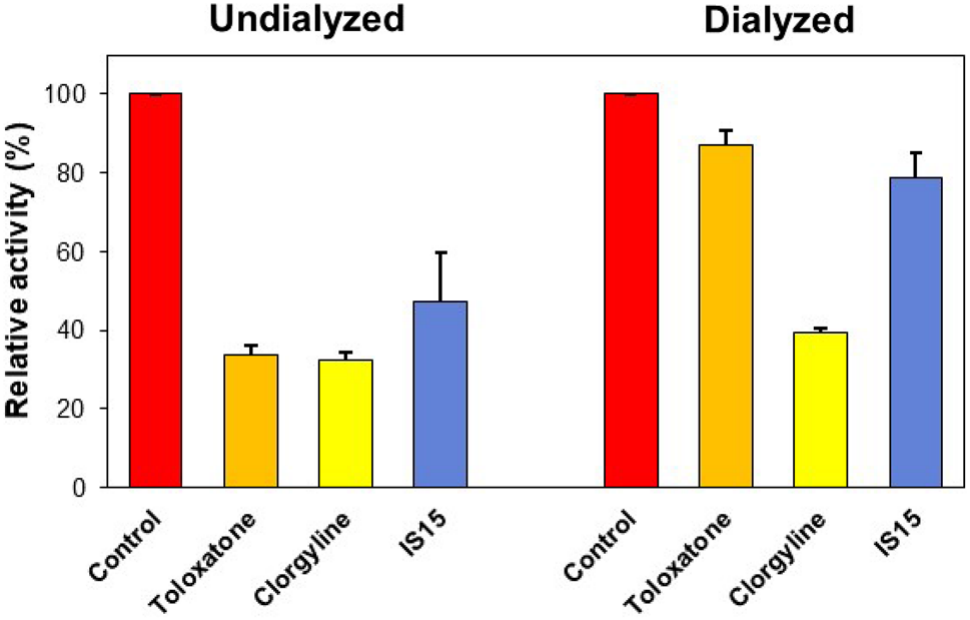
Recovery of MAO-A inhibition by **IS15** using dialysis experiments.

**Figure 5:**
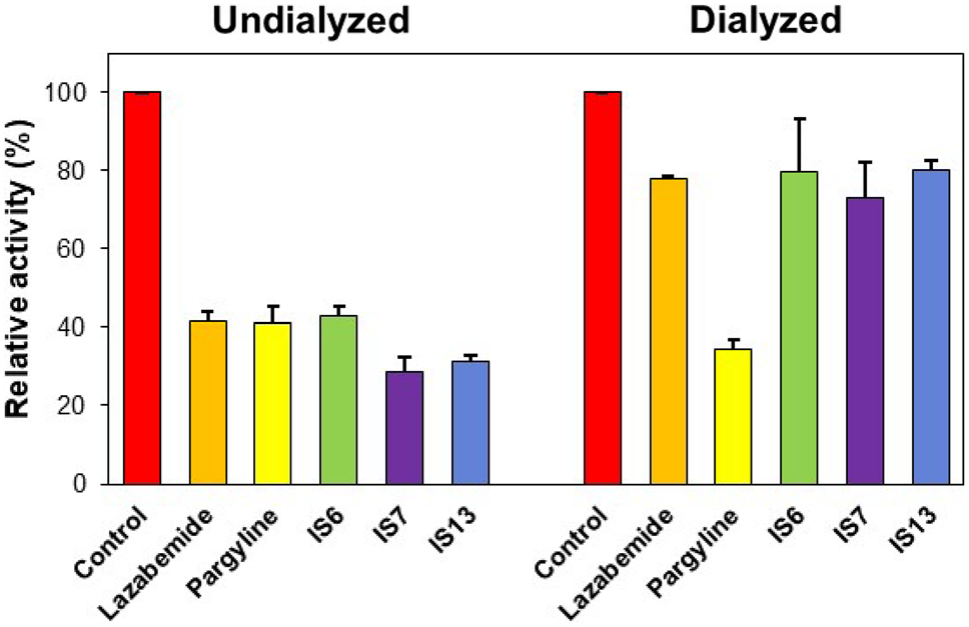
Recovery of MAO-B inhibition by **IS6**, **IS7**, and **IS13** using dialysis experiments.

Reversibility tests were performed using the dialysis method. In this study, the concentration of compound **IS15** used for MAO-A was 1.5 × that of the IC_50_ (3.00 μM), and those of compounds **IS6**, **IS7**, and **IS13** used for MAO-B were 1.5 × that of the IC_50_ (0.18, 0.12, and 0.15 μM, respectively). Recovery patterns were compared using undialyzed (A_U_) and dialyzed (A_D_) relative activity after 30 min of pre-incubation. For MAO-A inhibition, compound **IS15** recovered from 47.16 to 78.73% (Fig. 4). The recovery of the compound was similar to that of toloxatone (from 33.76 to 87.22%), and it could be distinguished from clorgyline (from 32.32 to 39.23%). For MAO-B inhibition, compounds **IS6**, **IS7,** and **IS13** recovered from 42.81 to 79.52%, 28.65–72.89%, and 31.45–80.12%, respectively (Fig. 5). The recovery values of the compounds were similar to those of lazabemide (from 41.48 to 77.71%) and could be distinguished from those of pargyline (from 41.04 to 34.34%). These results indicate that **IS15** is a reversible inhibitor of MAO-A, whereas **IS6**, **IS7**, and **IS15** are reversible inhibitors of MAO-B.

#### Enzyme kinetics

**Figure 6:**
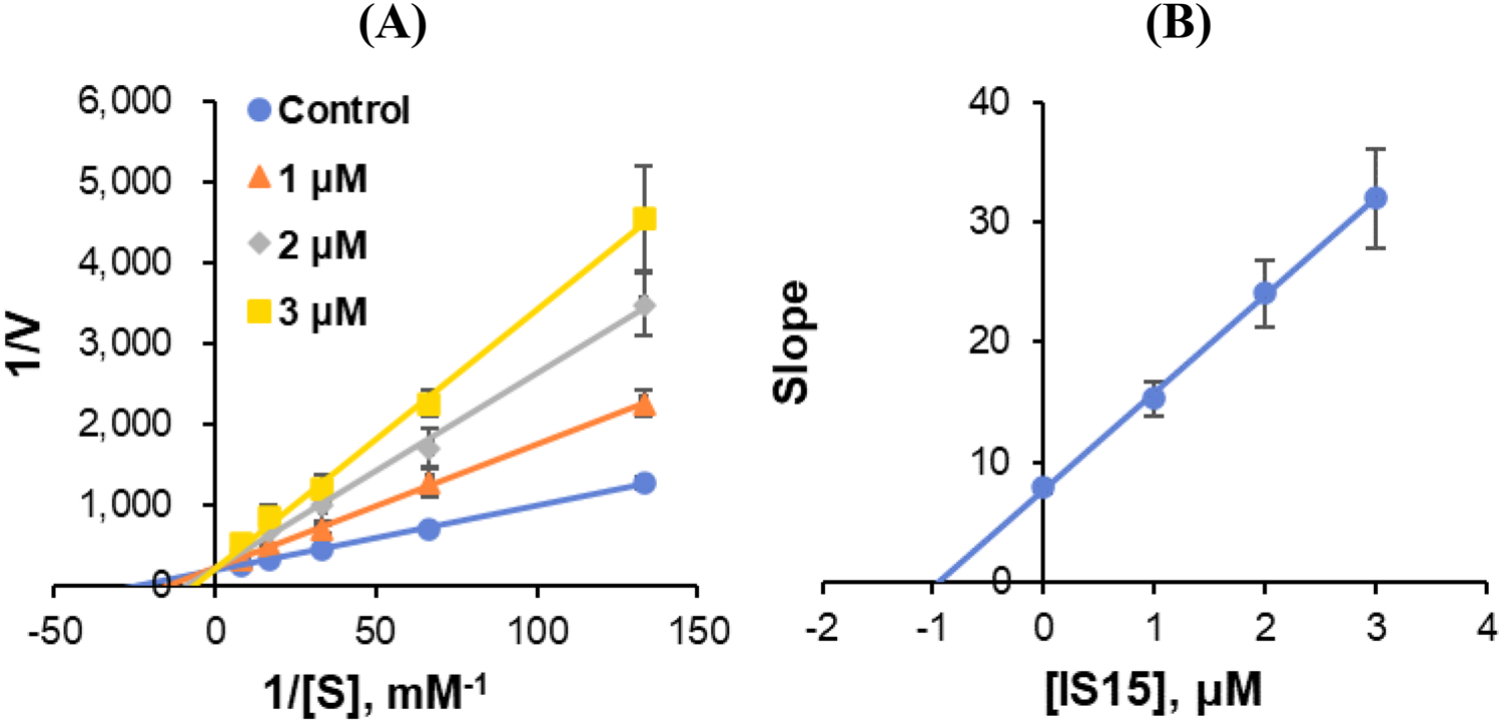
Lineweaver–Burk (LB) plots for MAO-A inhibition by **IS15** (**A**) and their respective secondary plots (**B**) of the slopes vs. inhibitor concentrations.

**Figure 7:**
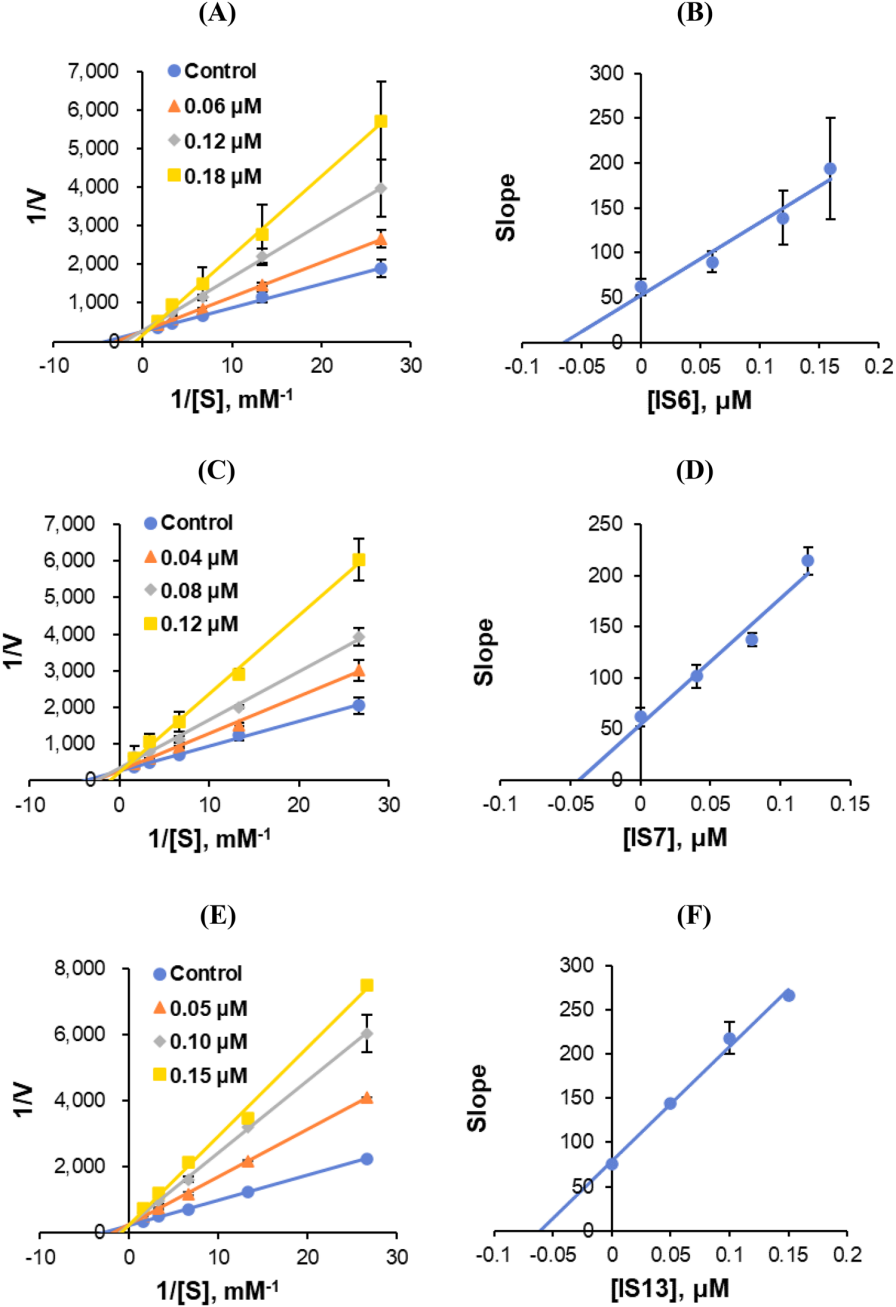
Lineweaver–Burk (LB) plots for MAO-B inhibition by **IS6**, **IS7**, and **IS13** (**A**, **C**, and **E**, respectively), and their respective secondary plots (**B**, **D**, and **F**, respectively) of the slopes vs. inhibitor concentrations.

The enzyme kinetics and inhibition types were analyzed at five substrate concentrations and three inhibitor concentrations. In the LB plot, **IS15** showed was a competitive MAO-A inhibitor (Fig. 6A), and the secondary plot revealed that the K_i_ value was 1.004 ± 0.171 μM (Fig. 6B). In contrast, **IS6**, **IS7**, and **IS13** LB plots indicated competitive MAO-B inhibitors (Fig. 7A, C, and E), and the secondary plots showed that their K_i_ values were 0.068 ± 0.022, 0.044 ± 0.002, and 0.061 ± 0.001 μM, respectively (Fig. 7B, D, and F). The K_i_ value of the inhibitor was calculated by the secondary plot constructed with each slope vs. inhibitor concentration in LB plot. The minus value of X-axis of the plot means − K_i_. Though **IS6** and **IS7** were not exactly intercepted on one point of Y-axis, V_max_ values in the presence of the inhibitors were almost same within the experimental error range, indicating both also were competitive inhibitors. In the presence of the inhibitors **IS6**, **IS7**, **IS13**, and **IS15**, K_m_ values were increased and V_max_ values were the same as the control. These results suggest that **IS15** is a competitive MAO-A inhibitor, whereas **IS6**, **IS7**, and **IS13** are competitive MAO-B inhibitors.

#### PAMPA assay

The PAMPA assay demonstrated that isatin-based hydrazone derivatives (**IS6**, **IS7**, **IS13**, and **IS15**) had high permeability and CNS bioavailability, with *P*e values of > 4.00 × 10^–6^ cm/s (Table 2). Brain penetration is crucial for the efficient administration of CNS medication^61^. The effective permeability of the chemical and the equation were used to calculate the penetration rate (Log Pe). A compound is categorized as potentially permeable (CNS+), if its P*e* value is > 4.00 × 10^–6^ cm/s, and perhaps non-BBB permeable (CNS-), if < 2.00 × 10^–6^ cm/s. This study showed that while halogenated isatin has BBB permeability, the substitution of the phenyl ring results in greater penetration. Chloro substitution resulted in higher BBB permeability, as revealed in this study.

**Table 2:** Blood–brain barrier (BBB) assay of key compounds of isatin-based hydrazone derivatives by the parallel artificial membrane permeability assay (PAMPA) method.

Compound	Experimental *P*e (× 10^−6^ cm/s)	Prediction
IS6	4.86 ± 0.26	CNS+
IS7	5.02 ± 0.19	CNS+
IS13	4.66 ± 0.18	CNS+
IS15	4.39 ± 0.30	CNS+
Selegiline	5.69 ± 0.04	CNS+

*Pe* (10^−6^ cm/s) > 4.00: CNS + (high permeation); *Pe* (10^−6^ cm/s) < 2.00: CNS − (low permeation); *Pe* (10^−6^ cm/s) from 2.00 to 4.00: CNS ± (BBB permeation uncertain).

#### MTT, ROS, anti-oxidant, and anti-inflammatory characteristics of the cell line-based assay

**Figure 8:**
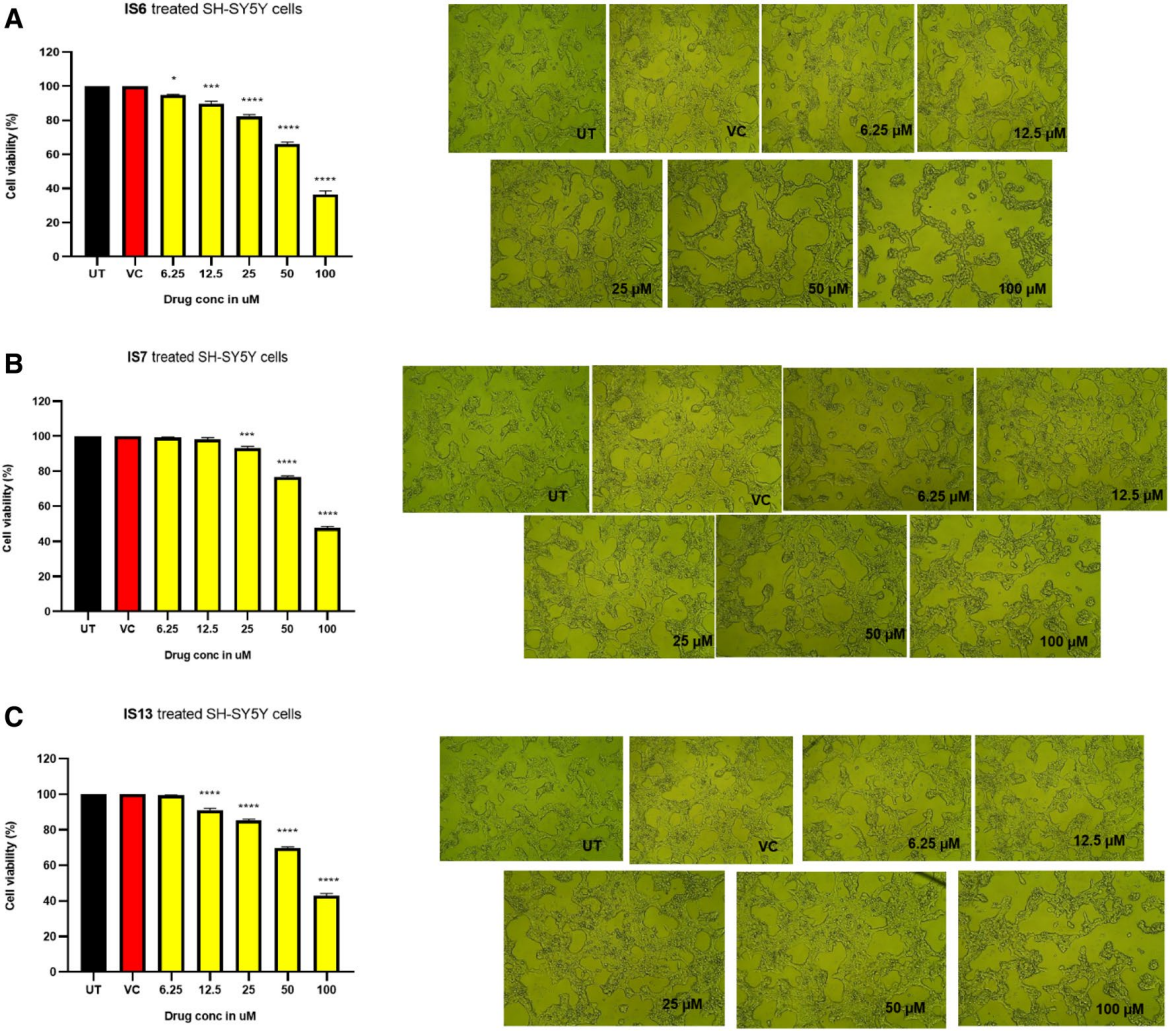
MTT assay of the lead compounds using SH-SY5Y cell for cell viability and morphological studies under a phase-contrast microscope, exposed for 24 h. (**A**) **IS6**, (**B**) **IS7**, and (**C**) **IS13**. Values are expressed as mean ± SEM. ***, *p* < 0.001; **, *p* < 0.01; *, *p* < 0.05 vs VC. VC, vehicle control; UT, untreated.

Cytotoxic activity of **IS6**, **IS7**, and **IS13** was tested by MTT assay using the human neuroblastoma SH-SY5Y cells. The cytotoxicity level was assessed by how well the live cells converted the tetrazolium dye into formazan crystals, with the untreated cells serving as the control group. **IS6**, **IS7**, and I**S13** remarkably decreased cell viability at 100 μM. However, IC_50_ values of **IS6**, **IS7**, and **1S13** were 75.72, 97.15, and 85.30 μM, respectively, indicating that these were cell- proliferative and non-cytotoxic in nature or working low concentration to SH-SY5Y cells (Fig. 8).

The preliminary in vitro neuroprotective activity against LPS-induced inflammatory events was tested using the synthesized **IS6**, **IS7**, **IS13**, and **IS15** in SH-SY5Y neuroblastoma cell lines. Cell viability assays and ELISA measurements of the intracellular pro-inflammatory cytokines TNF-alpha, IL-6, and NF-kB were used to evaluate neuroprotective activity. A common technique is to incubate SH-SY5Y cell lines with LPS (10 ng/mL) in minimum necessary medium at 37 °C for 24 h to induce neuroinflammation. We previously discovered that a 2 h incubation period with 1 ug/ml of LPS is adequate to trigger inflammatory responses.

**Figure 9:**
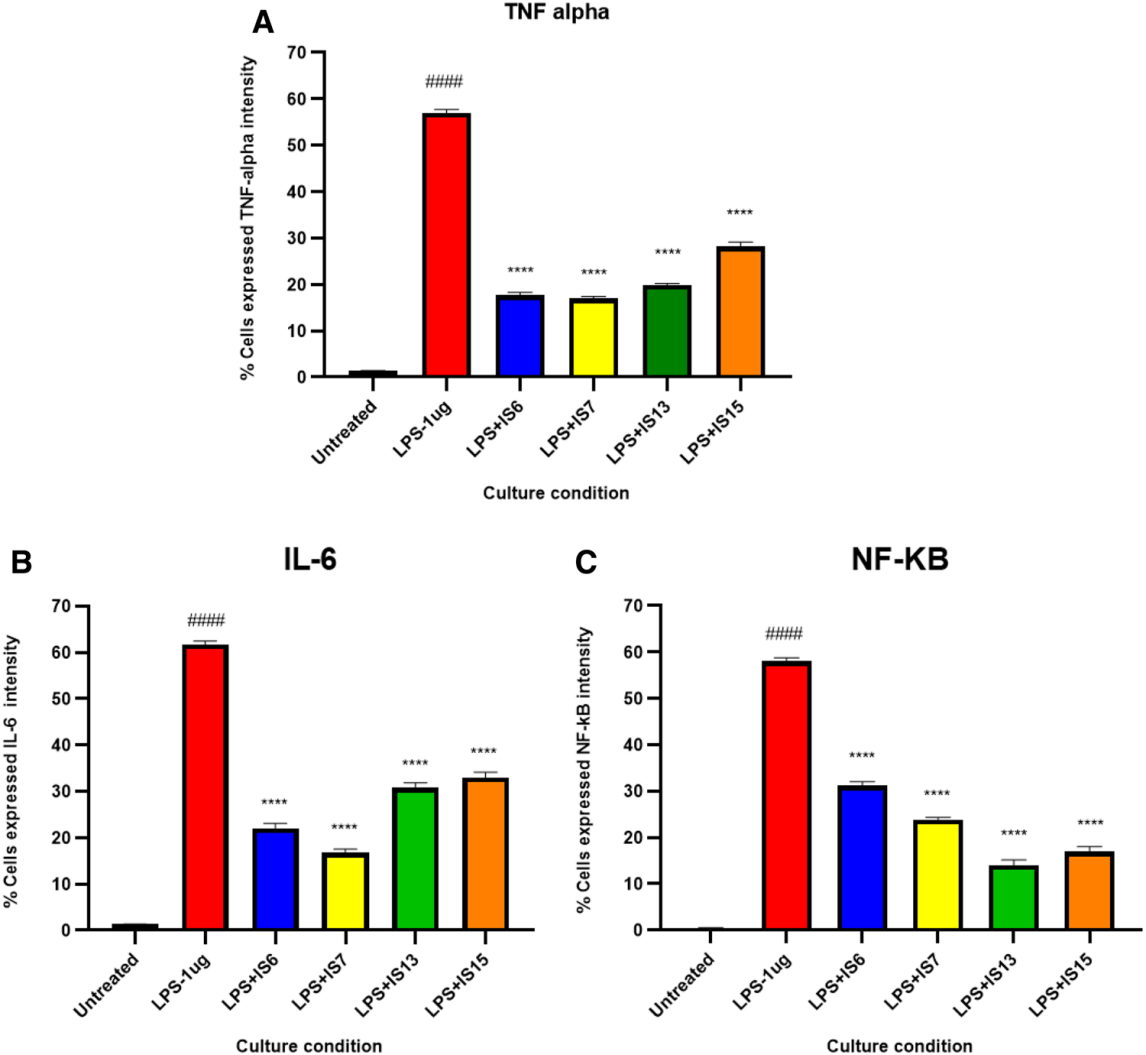
Effect of the lead compounds on anti-neuroinflammation in lipopolysaccharide (LPS)-induced SH-SY5Y cells. (**A**) Tumor necrosis factor (TNF)-α, (**B**) Interleukin (IL)-6, and C) Nuclear factor (NF)-kB. Values are expressed as mean ± SEM. ####, *p* < 0.0001 vs untreated; ****, *p* < 0.0001 vs LPS.

LPS treatment significantly raised the levels of IL-6, TNF-alpha, and NF-kB in LPS-intoxicated SH-SY5Y cell lines compared to control SH-SY5Y cell lines, demonstrating the magnitude of inflammatory reactions mediated by LPS toxicity (Fig. 9). **IS6**, **IS7**, **IS13**, and **IS15** pretreatment significantly (*p* < 0.0001) reduced TNF-alpha (Fig. 9A), IL-6 (Fig. 9B), and NF-kB (Fig. 9C) levels in comparison to the LPS-treated group, demonstrating the anti-inflammatory potential of isatin derivatives (Figs. S49, S50). Intriguingly, in LPS-treated cell lines, **IS6** and **IS7** significantly decreased the levels of TNF-alpha and IL-6 compared to **IS13** and **IS15**. When compared to **IS6** and **IS7**, **IS15** and **IS13** have significantly lower NF-kB expression. The lead compounds confirmed the anti-inflammatory potential of all the compounds in the human neuroblastoma model by inhibiting LPS-induced pro-inflammatory cytokine expression (IL-6, TNF-alpha, and NF-kB).

**Figure 10:**
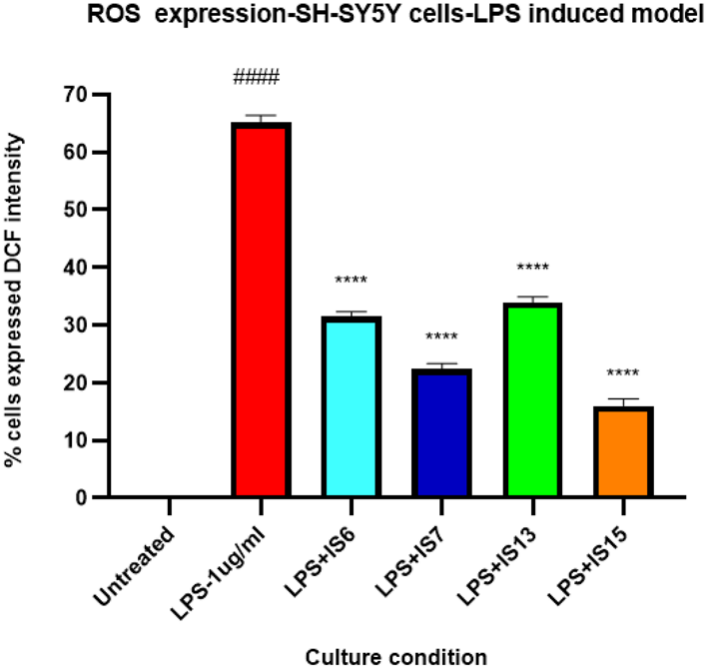
Effect of the lead compounds on reduction of ROS generation in SH-SY5Y cells. Values are expressed as mean ± SEM. ####, *p* < 0.0001 vs untreated; ****, *p* < 0.0001 vs LPS.

**Figure 11:**
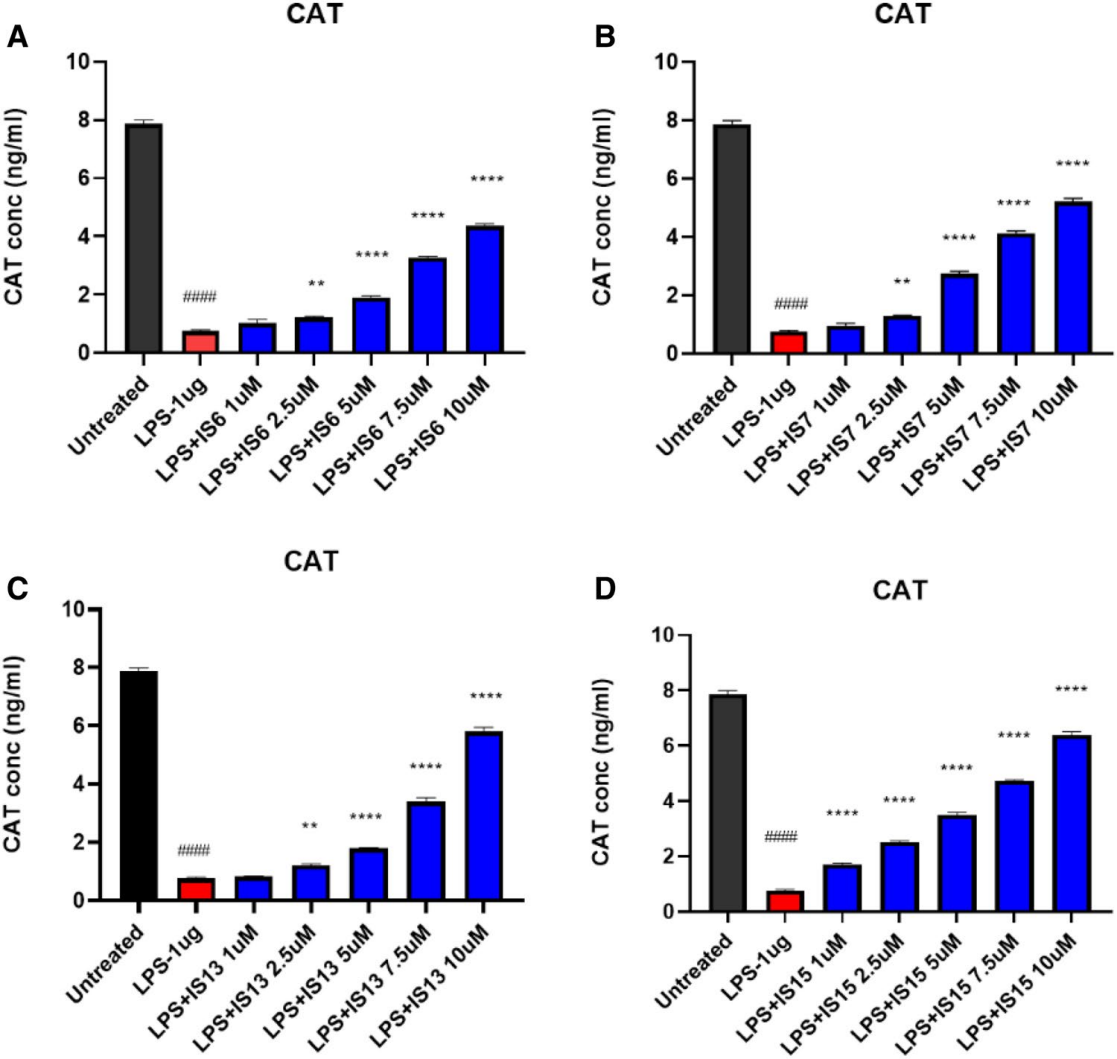
Catalase (CAT) levels observed in different concentrations of (**A**) **IS6**, (**B**) **IS7**, (**C**) **IS13**, and (**D**) **IS15** treatment against the LPS-induced SH-SY5Y cells. Values are expressed as mean ± SEM. ####, *p* < 0.0001 vs untreated; ****, *p* < 0.0001; **, *p* < 0.01 vs LPS.

**Figure 12:**
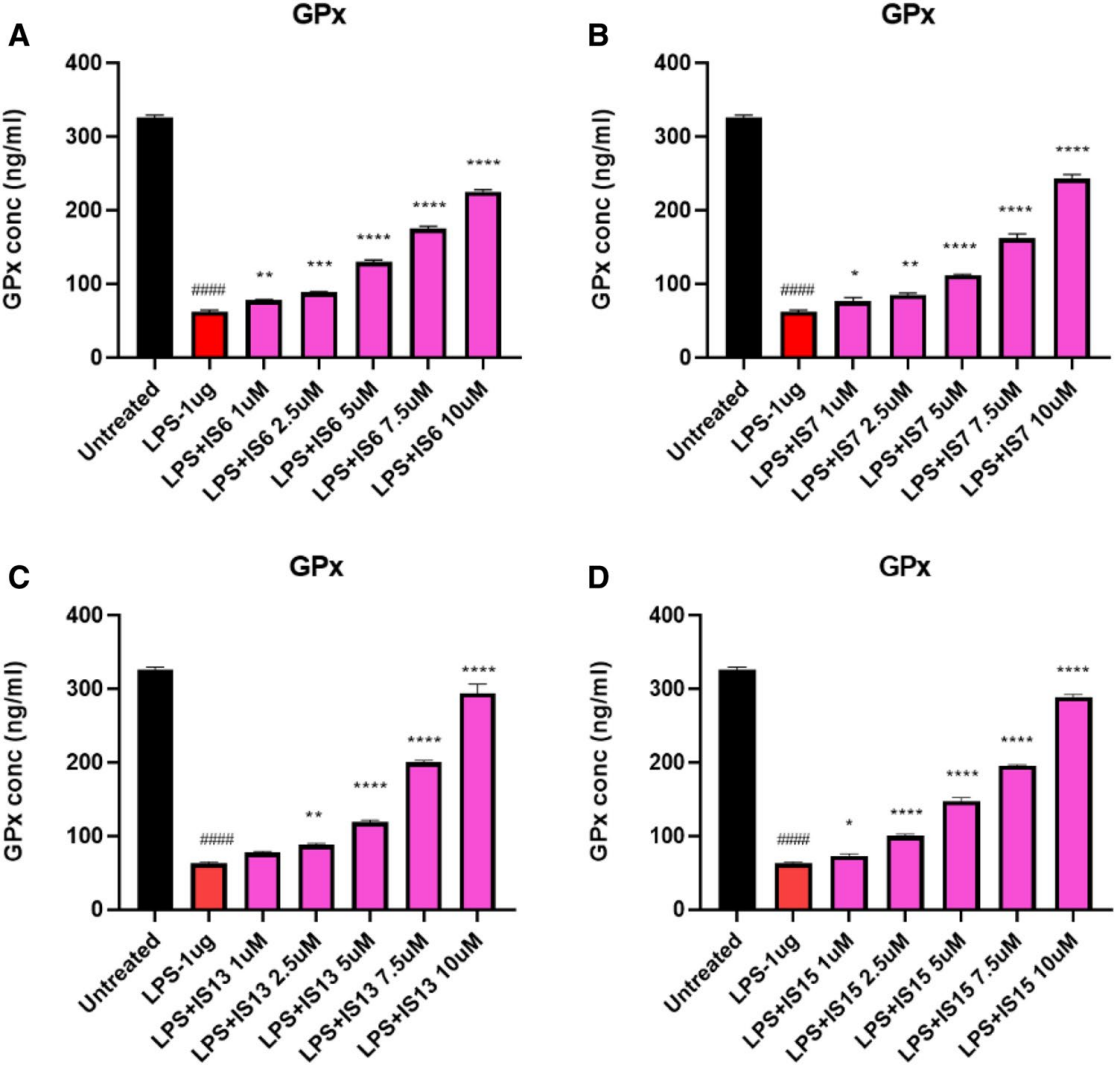
Glutathione peroxidase (GPx) levels observed in different concentrations of (**A**) **IS6**, (**B**) **IS7**, (**C**) **IS13**, and (**D**) **IS15** treatment against the LPS-induced SH-SY5Y cells. Values are expressed as mean ± SEM. ####, *p* < 0.0001 vs untreated; ****, *p* < 0.0001; ***, *p* < 0.001; **, *p* < 0.01; *, *p* < 0.05 vs LPS.

**Figure 13:**
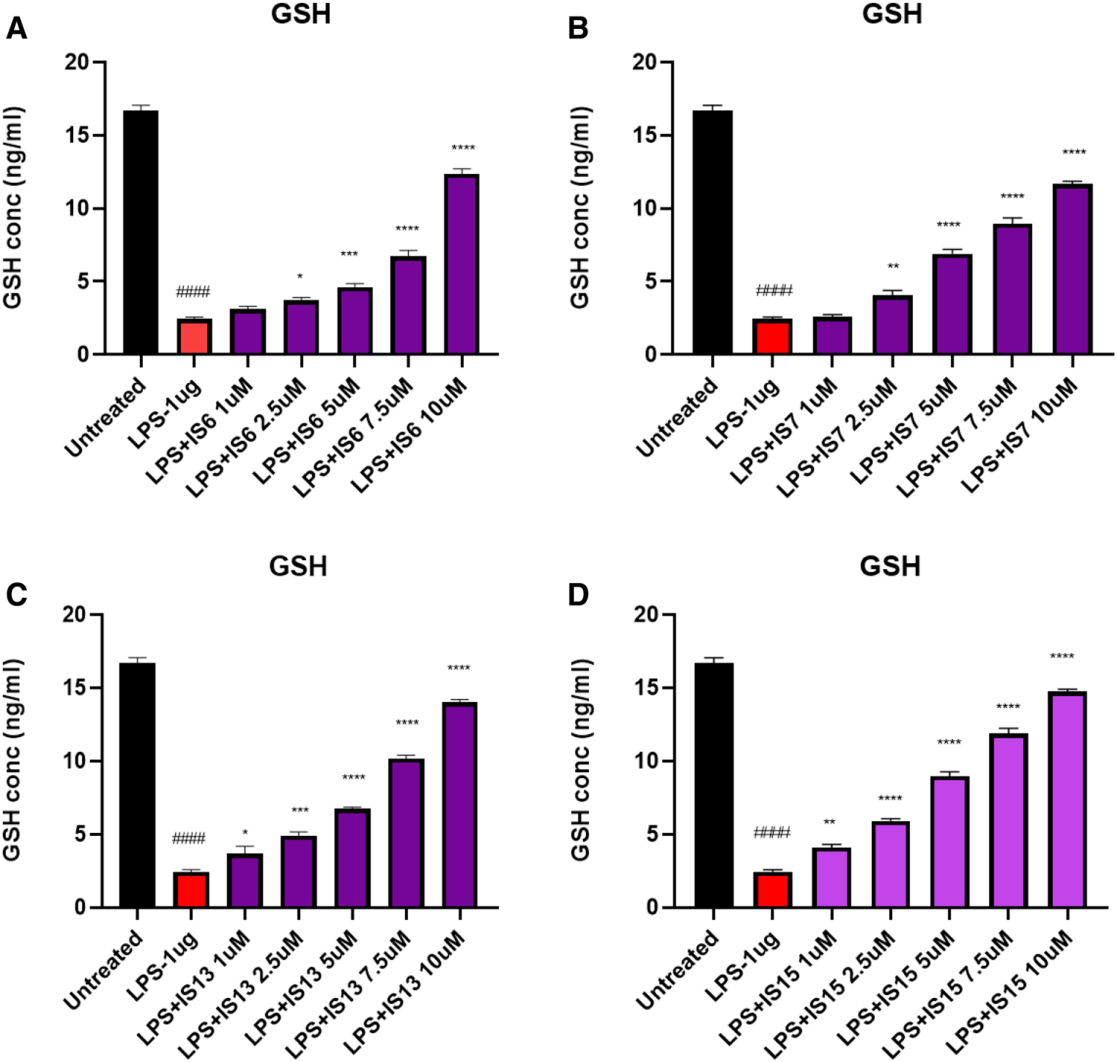
Glutathione levels observed in different concentrations of (**A**) **IS6**, (**B**) **IS7**, (**C**) **IS13**, and (**D**) **IS15** treatment of the LPS-induced SH-SY5Y cells. Values are expressed as mean ± SEM. ####, *p* < 0.0001 vs untreated; ****, *p* < 0.0001; ***, *p* < 0.001; **, *p* < 0.01; *, *p* < 0.05 vs LPS.

**Figure 14:**
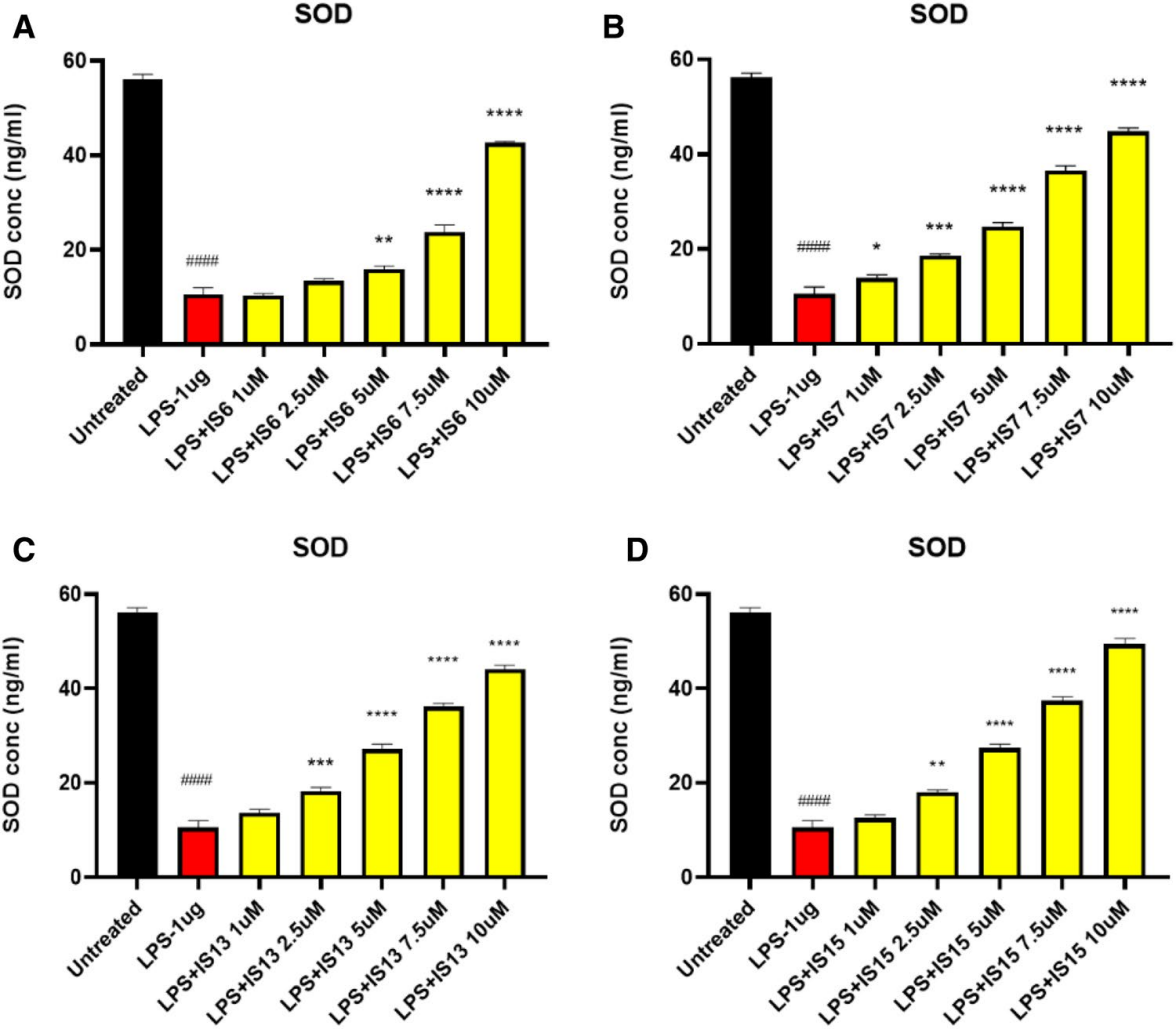
Superoxide dismutase (SOD) levels observed in different concentrations of (**A**) **IS6**, (**B**) **IS7**, (**C**) **IS13**, and (**D**) **IS15** treatment of the LPS-induced SH-SY5Y cells. Values are expressed as mean ± SEM. ####, *p* < 0.0001 vs untreated; ****, *p* < 0.0001; ***, *p* < 0.001; **, *p* < 0.01; *, *p* < 0.05 vs LPS.

To further confirm the neuroprotective and anti-oxidant effects of the lead compounds, the effect of the lead compounds on decrease of ROS production using the LPS-treated SH-SY5Y cells (Fig. 10). The compounds **IS6**, **IS7**, **IS13,** and **IS15** significantly inhibited (*p* < 0.0001) 2′,7′-dichlorofluorescin (DCF) expression in the LPS-induced model compared to cells treated with LPS alone. The maximum concentration of test compounds that significantly inhibited LPS inflammation was considered in the ROS study. The lead compounds, **IS15** and **IS7**, at 10 µM/mL concentrations, exhibited an effective DCF intensity decrease compared to the LPS-induced cells, whereas **IS6** and **IS13**, at 10 µM concentrations, exhibited moderate DCF intensity suppression (Fig. S51). LPS alone induced 65% of DCF expression. The cellular anti-oxidant assay results suggested that the lead compounds showed significant neuroprotective activity by enhancing the cellular oxidant enzymes CAT, GPx, GSH, and SOD (Figs. 11, 12, 13 and 14) in the LPS-induced model, while cells treated with LPS alone effectively expressed SOD, CAT, GSH, and GPx activities. The obtained values confirmed the promising neuroprotective and neuro-inflammatory activity of **IS6**, **IS7**, **IS13,** and **IS15** compounds (at 10 µM/mL) (*p* < 0.0001) in relation to the neuroprotective effect in the LPS-induced human neuroblastoma model by enhancing the enzyme activity and inhibiting the oxidative stress-induced apoptosis caused by LPS by determining DCF intensity.

#### Molecular docking

**Figure 15:**
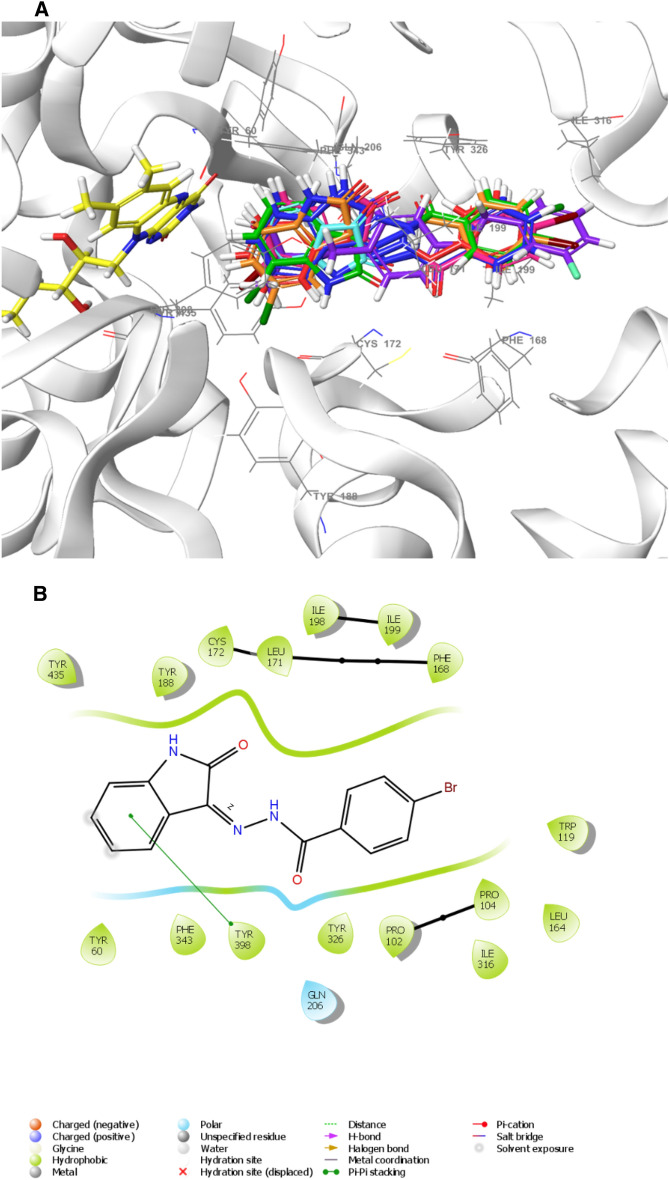
**A**) 3-D visualization of superimposed orientations of **IS3** (pink), **IS6** (red) **IS7** (green), **IS13** (blue), **IS15** (orange), isatin (cyan), and safinamide (violet) in the active site of MAO-B and the co-factor flavin adenine dinucleotide (FAD) (yellow). **B**) 2-D interaction of lead inhibitor **IS7** with MAO-B pocket.

Molecular docking studies were performed to better understand the binding processes of lead compounds. Interactions occurred between the molecules **IS3**, **IS6**, **IS7**, **IS13**, and **IS15**, with MAO-B (2V5Z) and MAO-A (2Z5X). Native ligands were used to confirm docking^57^. The compounds were mentioned in Table 3, docking scores (XP mode) ranges were − 6.55 to − 10.85 kcal/mol. The scores of the best lead molecules **IS6**, **IS7**, and **IS13**, through biological evaluation, were − 9.47, − 9.88, and − 9.72 kcal/mol, respectively, while safinamide had a score that was comparable (− 10.85 kcal/mol). The docking scores showed similar affinity like biological activity (IC_50_). When looking into the orientations, the fluorobenzyl group of safinamide was placed towards the opening of the cavity, whereas the amide side chain pointed in the direction of the FAD molecule. A similar orientation was present in the compounds **IS3**, **IS6**, **IS7**, **IS13**, **IS15**, and isatin (Fig. 15A), where the phenyl group was positioned towards the cavity opening and the variable isatin moiety was oriented towards FAD. The entrance and substrate cavities of the MAO-B-binding pocket (Fig. 15B) were completely occupied with all inhibitors. The best lead molecule (**IS7**) interacted with Tyr60, Pro102, Pro104, Trp119, Phe168, Leu171, Cys172, Tyr326, and Tyr435 were primarily hydrophobic, whereas Gln206 was in polar contact. The isatin moiety demonstrated that Pi-Pi stacking with Tyr398 provided the **IS7**-MAO-B protein with complex stability.

**Table 3:** Docking score of MAO-A and MAO–B with IS3, IS6, IS7, IS13, IS15, and their native ligands.

Compound	Docking score (kcal/mol)	
	MAO-A	MAO-B
IS3	− 7.65	− 9.16
IS6	− 3.96	− 9.47
IS7	− 4.32	− 9.88
IS13	− 4.93	− 9.72
IS15	− 8.00	− 9.36
Isatin	–	− 6.55
Harmine	− 6.04	–
Safinamide	–	− 10.85

**Figure 16:**
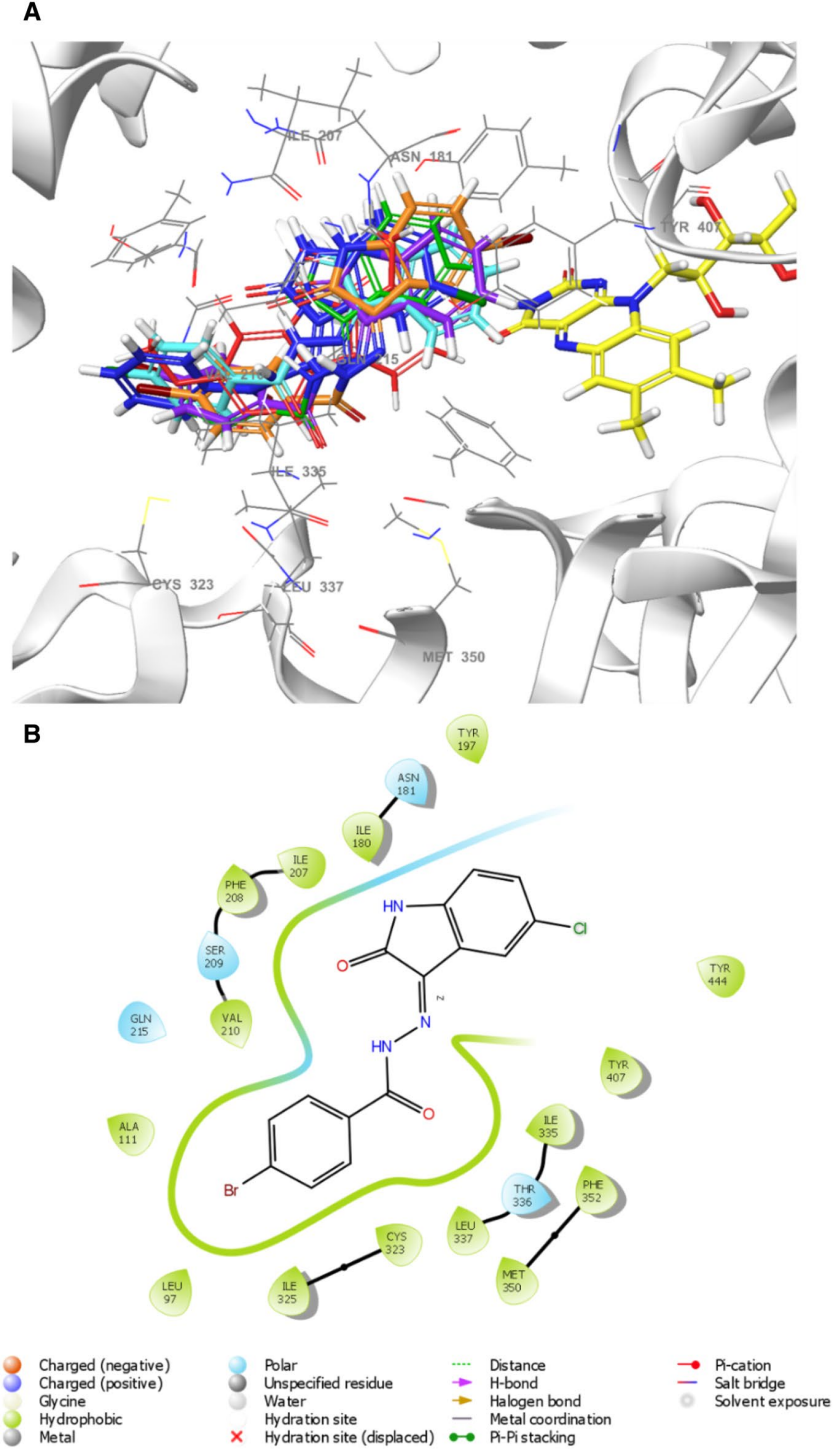
(**A**) 3-D visualization of superimposed orientations of **IS3** (orange), **IS6** (cyan) **IS7** (violet), **IS13** (blue), **IS15** (green), and harmine (red) in the active site of MAO-A and the co-factor FAD (yellow). (**B**) 2-D interaction of lead inhibitor **IS15** with MAO-A pocket.

For MAO-A, the docking score range is − 3.96 to − 8.00 kcal/mol (Table 3). The **IS15** and Harmine docking scores (XP mode) were − 8.00 and − 6.04 kcal/mol, respectively. Comparing the docking score to each compound’s IC_50_, **IS15** had the best profile, followed by **IS3** (− 7.65 kcal/mol), **IS13** (− 4.93 kcal/mol), **IS7** (− 4.32 kcal/mol), and **IS6** (− 3.96 kcal/mol). Every molecule was oriented in the same way as its native ligand, and its isatin moiety was always closed to the substrate cavity (FAD) (Fig. 16A). Thorough analysis of compound **IS15** in the MAO-A active site revealed that it was present at the following positions: Leu97, Ala111, Ile180, Asn181, Tyr197, Ile207, Phe208, Ser209, Val210, Gln215, Cys323, Ile325, Thr326, Ile335, Leu337, Tyr407, and Tyr444 (Fig. 16B). Through hydrophobic contacts, the phenyl ring of **IS15** interacted with Leu97, Ala111, Val210, Ile325, and Cys323. Residues Ile180, Asn181, Tyr197, Tyr407, and Tyr444 interacted with the isatin ring through hydrophobic and polar interactions. Its similar interaction with **IS15** and harmine^62^ during the binding contact demonstrated its potential to inhibit MAO-A.

#### Molecular dynamic simulation

**Figure 17:**
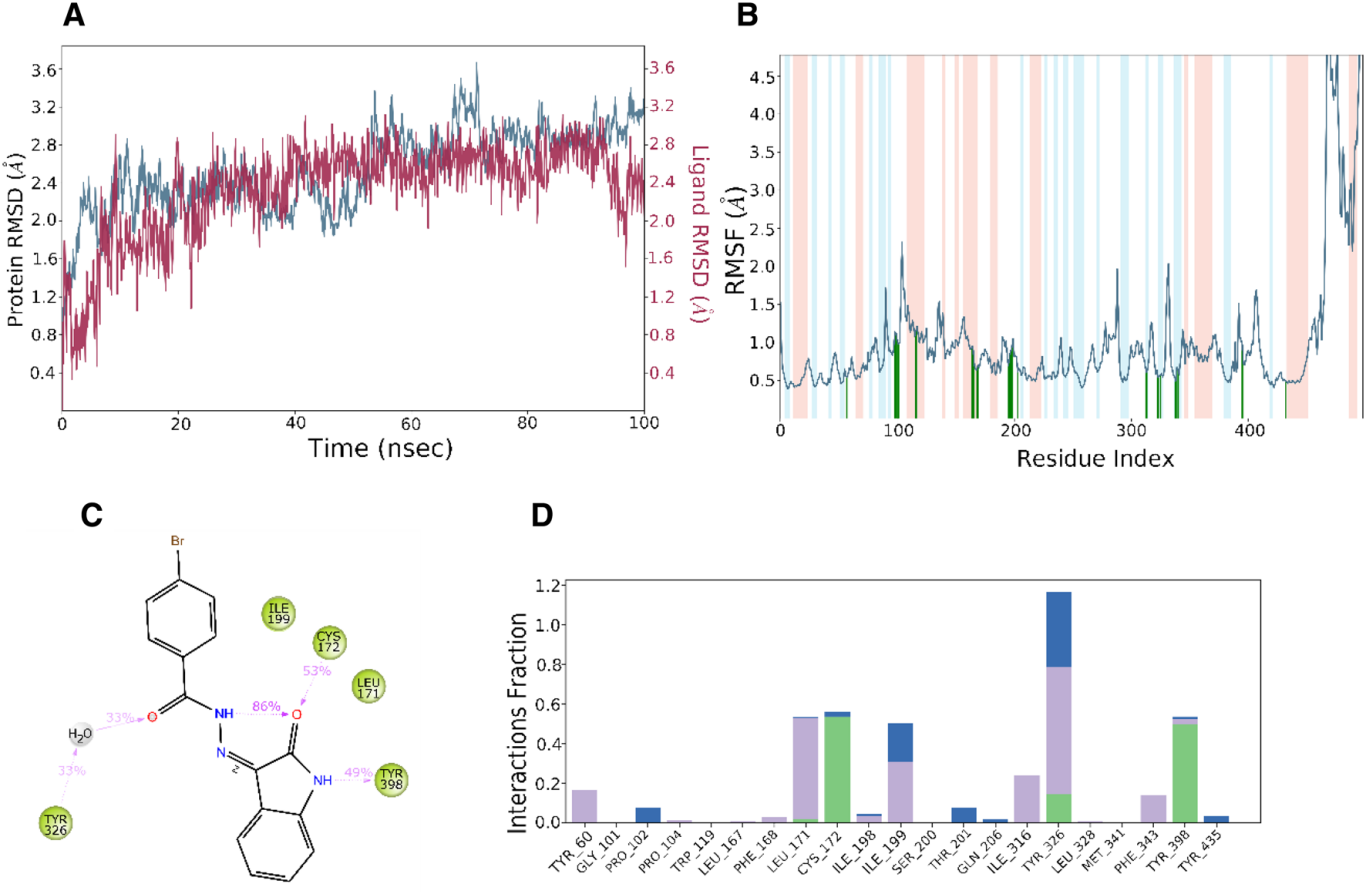
Desmond’s MD simulation analysis of the **IS7**-MAO-B complex. (**A**) Root mean square deviation (RMSD) (protein and **IS7** RMSD are shown in blue and red, respectively). (**B**) Individual RMSF for proteins' amino acids. (**C**) Diagram of the 2-D Interaction. (**D**) Protein–ligand contacts with number of specific contacts of amino acids with **IS7**.

Desmond MD simulations were used to follow the binding mode of **IS7** in the inhibitor-binding cavity (IBC) of MAO-B. Protein C-alpha and ligand were tracked within an acceptable range for a long simulation duration (100 ns) according to root mean square deviation (RMSD) analysis. In contrast to the protein RMSD, the ligand (red) RMSD remained steady after 25 ns. The protein RMSD ranged between 1.2 and 3.6 Å with an average of 2.54 ± 0.01 Å (Fig. 17A). The protein-specific RMSD for the simulation was constant, with the exception of a slight variation, reaching a maximum of 3.6 Å at 68–70 ns, where after it stabilized. The simulation evaluated the flexibility of the protein system by computing the RMSF for each amino acid residue of the protein. The 480–498 residues of MAO-B showed a larger fluctuation. The atoms in the benzoyl ring of the RMSF ligand (Fig. 17B) showed slight fluctuations during the binding process. The 21 amino acid residues that interacted with the ligand were Tyr60 (0.541 Å),Gly101 (1.13 Å), Pro102(1.117 Å), Pro104 (0.981 Å), Trp119 (1.15 Å), Leu167 (0.955 Å), Phe168 (0.892 Å), Leu171 (0.638 Å), Cys172 (0.706 Å), Ile198 (0.689 Å), Ile199 (0.833 Å), Ser200 (0.88 Å), Thr201 (0.94 Å), Gln206 (0.618 Å), Ile316 (0.604 Å), Tyr326 (0.544 Å), Leu328 (0.572 Å), Met341 (0.493 Å), Phe343 (0.642 Å), Tyr398 (0.97 Å), and Tyr435 (0.497 Å). Hydrogen bonds, hydrophobic contacts, and water bridges were identified in the interaction histograms of **IS7** and MAO-B (Fig. 17C and D). Over a trajectory of 100 ns, the number of individual interactions between the amino acids and ligand was normalized. Several significant amino acids, including Tyr326 (hydrogen bond, water bridge, and hydrophobic), Tyr398 (hydrogen bond), Leu171 (hydrophobic), Cys172 (hydrogen bond and water bridge), and Ile199 (water bridge and hydrophobic), interact with **IS7**. The measured fraction of interactions with Tyr326 was > 1.0. As previously observed^63,64^, the hydrophobic interaction of Tyr326 at the active site of MAO-B was significant. Figure 17C and D depict hydrogen bonding, water bridges, and hydrophobic stability in the ligand–protein complexes. Cys172 forms an 86% hydrogen bond with the carbonyl and NH atoms in the linker between the isatin and benzoyl rings. Tyr398 contributed 49% via hydrogen bonding with the NH atom of the isatin ring. With carbonyl and water molecules, Tyr326 is a 33% active participant in hydrogen bonding. Overall, it is estimated from the trajectory analysis and full MD simulation that the lead compound **IS7** will inhibit MAO-B.

#### MM-GBSA

From their MD simulation frames, the free binding energy was estimated for the best molecule **IS7** with the highest docking energy and activity value prediction. Total average energies of ΔG Bind, ΔG Bind H-bond, ΔG Bind Lipo, and ΔG Bind vdW was − 190.04, − 12.26, − 45.94, − 140.23 for 0–100 ns MD snapshot, respectively. Across all interactions, the ΔG Bind vdW and ΔG Bind Lipo energies exerted the most significant impact on the average binding energy (Table 4).

**Table 4:** Free binding energies of the molecule IS7 through MM-GBSA*.

MD (ns)	ΔG Bind	ΔG Bind H-bond	ΔG Bind Lipo	ΔG Bind vdW
0	− 181.24	− 10.01	− 52.45	− 161.63
10	− 198.99	− 11.61	− 55.85	− 159.96
20	− 185.63	− 7.16	− 48.34	− 119
30	− 208.04	− 15.31	− 52.77	− 129.35
40	− 225.93	− 11.7	− 54.78	− 135.01
50	− 211.25	− 14.81	− 45.46	− 137.82
60	− 156.03	− 11.38	− 34.52	− 107.01
70	− 190.78	− 11.32	− 51.15	− 140.55
80	− 191.13	− 14.03	− 34.11	− 155.83
90	− 200.74	− 16.38	− 45.74	− 177.62
100	− 140.75	− 11.15	− 30.27	− 118.82

* kcal/mol.

The values ΔG Bind vdW for the interactions of **IS7** with protein complexes indicated the presence of stable van der Waals interaction with amino acid residues. Consequently, the MM-GBSA calculations, derived from MD simulation trajectories, aligned well with the binding energies computed from the docking results. The molecule exhibited very low free binding energy, indicating its higher binding affinity towards the receptor. Consequently, it can be inferred that **IS7** compound exhibited a strong affinity for the MAO-B protein.

## Conclusion

We synthesized acylhydrazone-based isatin compounds and evaluated their ability to inhibit MAOs. **IS15** was a potent competitive reversible MAO-A inhibitor, whereas **IS6**, **IS7**, and **IS13** were potent competitive reversible and selective MAO-B inhibitors. A CNS permeability study using a PAMPA assay revealed that the lead compounds were BBB-permeable. The lead compounds also exhibit non-cytotoxic, neuroprotective and anti-inflammatory effects. The lead compounds (at a concentration of 10 µM/mL) effectively reduced DCF intensity. Additionally, a docking analysis of MAO-B and **IS7** revealed the stability of the complex due to the pi–pi stacking of Tyr326. The Cys172 residue participated in the interaction with the ligand at 86% during dynamic examination. Finally, MM-GBSA energy binding revealed that **IS7** provided strong stability to MAO-B protein. Overall, the results of this investigation suggest that the lead compounds, **IS7**, **IS6**, **IS13**, and **IS15,** may be viable therapeutic agents for the treatment of neurological disorders such as PD.

### Supplementary Information

Supplementary Information.
