# Supplementary material for: Isatin-tethered halogen-containing acylhydrazone derivatives as monoamine oxidase inhibitor with neuroprotective effect
Source: Sci Rep. 2024 Jan 13;14:1264. doi: 10.1038/s41598-024-51728-x (PMC10787790; doi:10.1038/s41598-024-51728-x)
Supplement: Supplementary file 1 — Supplementary Information. [file 41598_2024_51728_MOESM1_ESM.docx]

**Isatin-tethered halogen-containing acylhydrazone derivatives as monoamine oxidase inhibitor with neuroprotective effect**

Sunil Kumar^a#^, Jong Min Oh^b#^, Prabitha Prabhakaran^c^, Abhimanyu Awasti^c^, Hoon Kim^b*^, Bijo Mathew^a*^

^a^ Department of Pharmaceutical Chemistry, Amrita School of Pharmacy, Amrita Vishwa Vidyapeetham, AIMS Health Sciences Campus, Kochi 682 041, India.

^b^ Department of Pharmacy, and Research Institute of Life Pharmaceutical Sciences, Sunchon National University, Suncheon 57922, Republic of Korea.

^c^ Department of Pharmaceutical Chemistry, JSS College of Pharmacy, JSS Academy of Higher Education and Research Mysuru 570015, India.

^#^Authors contributed equally.

^*^Corresponding Authors:

Bijo Mathew (B. Mathew) (bijomathew@aims.amrita.edu) (bijovilaventgu@gmail.com)

Hoon Kim (H. Kim) ([hoon@sunchon.ac.kr](mailto:hoon@sunchon.ac.kr))

***(Z)-N'-(5-bromo-2-oxoindolin-3-ylidene) benzohydrazide* (IS1)**: Yield 86%. NMR (400 MHz) δ 11.37 (1H, NH), 7.88–7.86 (m, 3H, Ar-H), 7.67 (m, 1H, Ar-H), 7.61-7.57(m, 2H, Ar-H), 7.24 (m, 1H, Ar-H), 6.94 (m 1H, Ar-H). ^13^C NMR (400 MHz) δ: 163.40, 141.66, 133.56, 132.38, 131.76, 129.75, 127.95, 127.44, 122.06, 121.02, 113.33. Molecular Formula: C_15_H_10_BrN_3_O_2_ (ESI) Calculated= 344.1628, Observed= 344.1698.


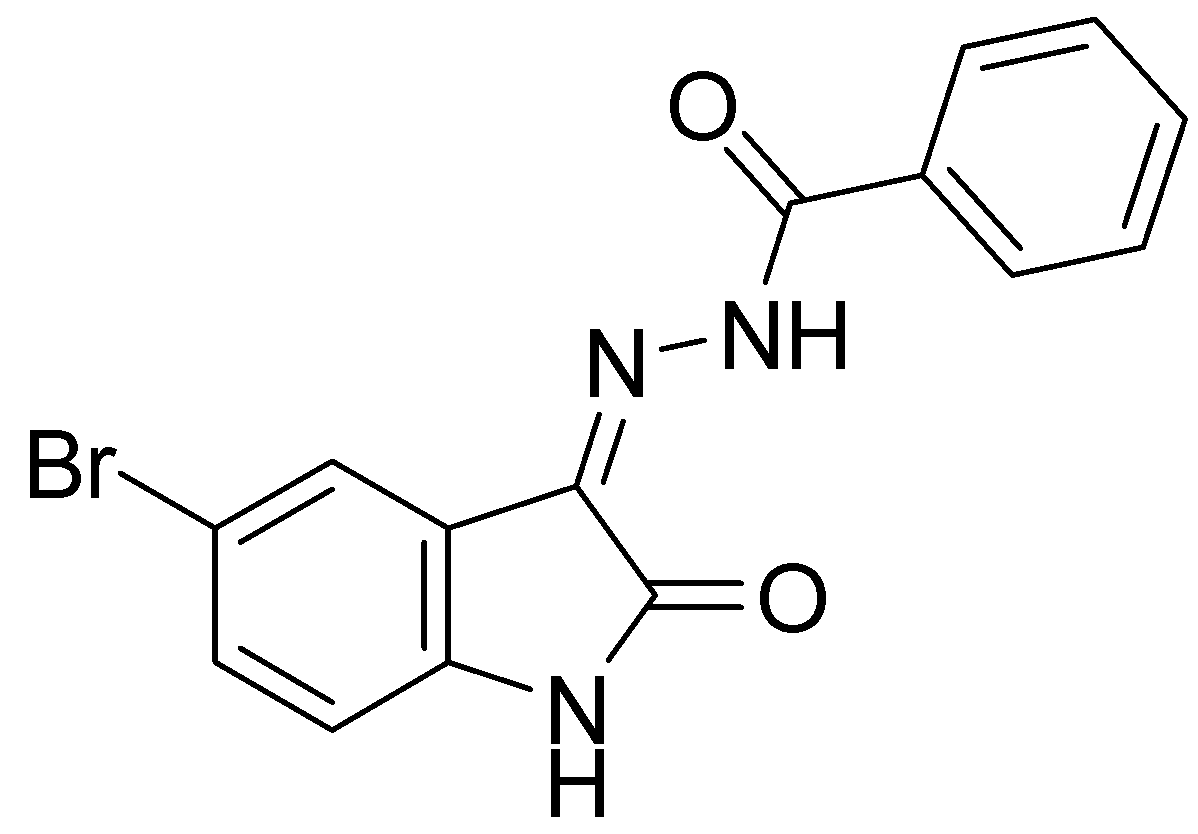


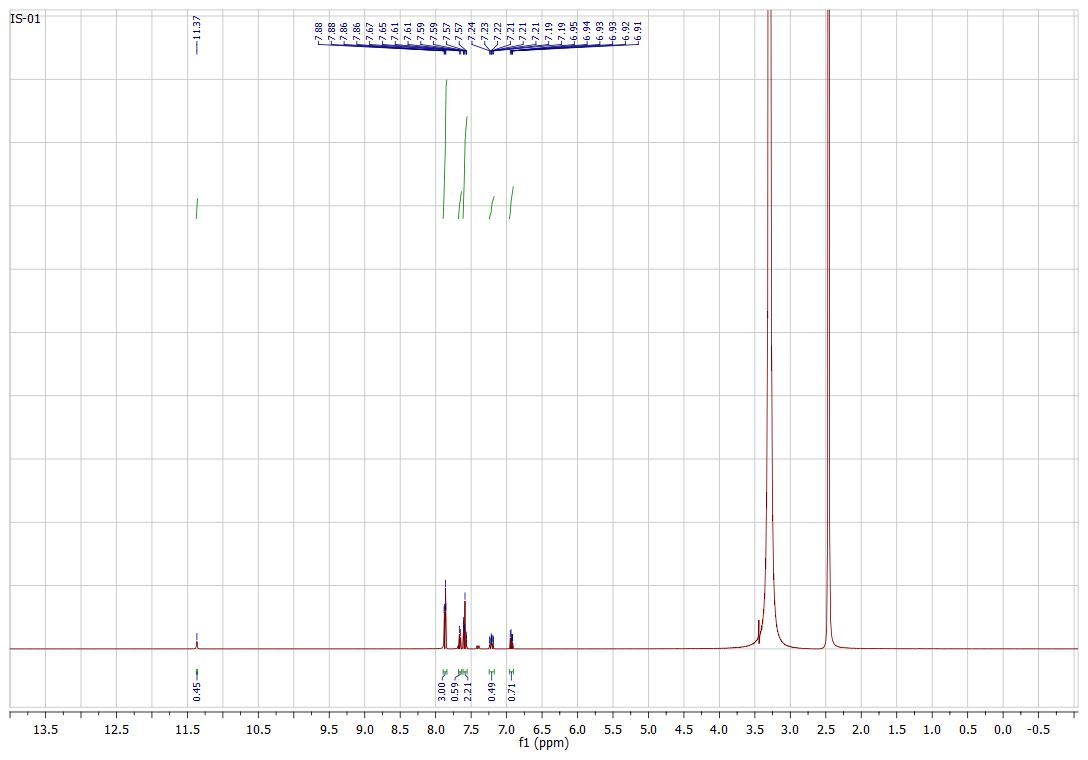


**Figure S1.** ^1^H NMR of **IS1** compound.


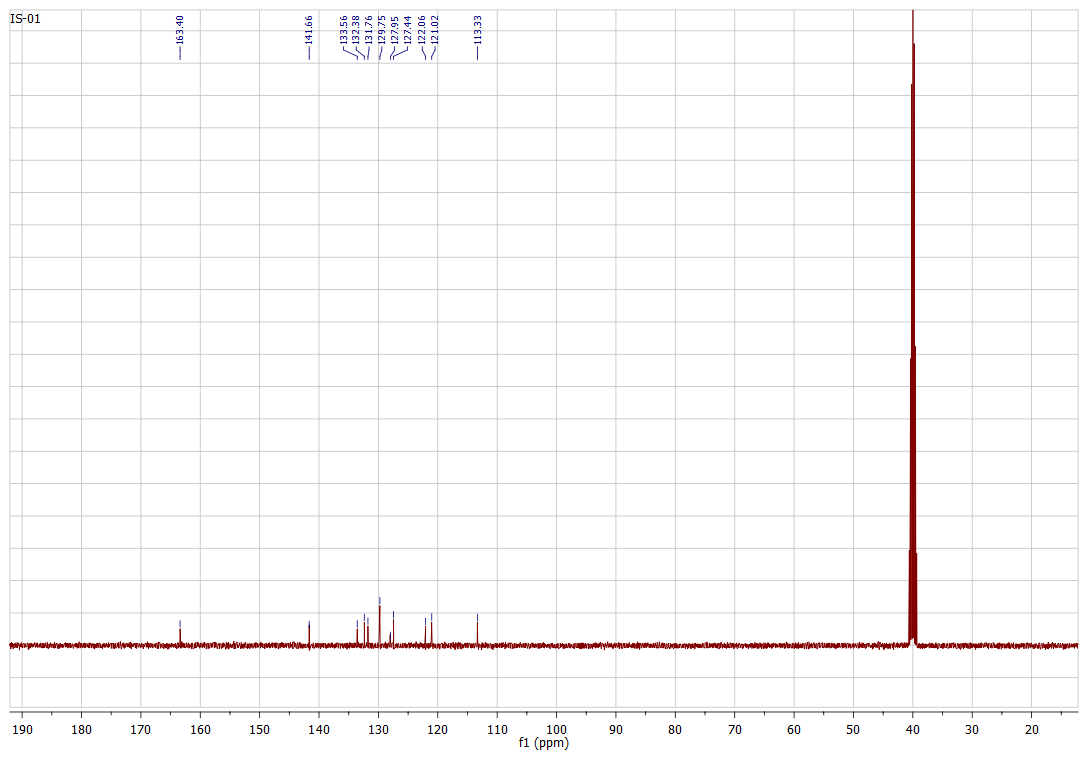


**Figure S2.** ^13^C NMR of **IS1** compound.


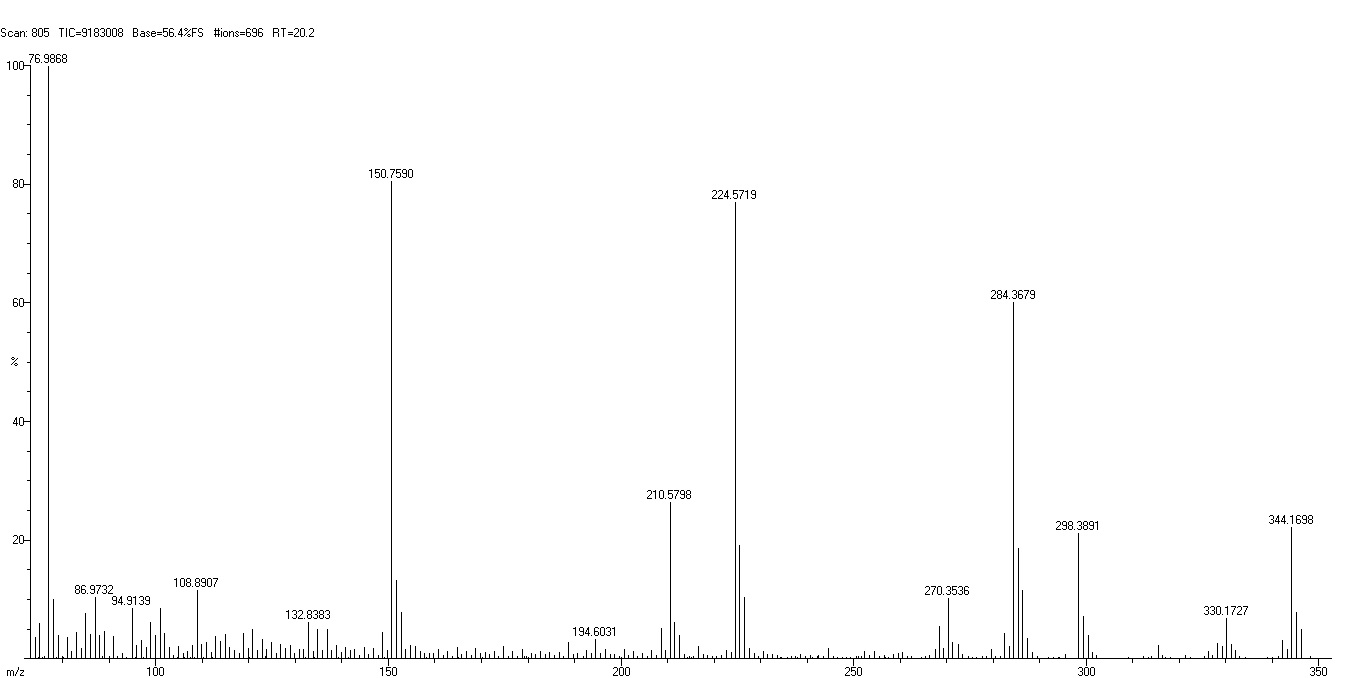


**Figure S3.** MASS of **IS1** compound.

***(Z)-N'-(5-bromo-2-oxoindolin-3-ylidene)-4-chlorobenzohydrazide (IS2):*** Yield 80%. ^1^H NMR (400 MHz) δ: 11.47 (1H, NH), 10.78 (1H, NH), 7.88 (m, 1H, Ar-H), 7.67 (m, 2H, Ar-H), 7.40 (m, 1H, Ar-H), 7.27 (m, 1H, Ar-H), 6.89 (m, 1H, Ar-H), 6.78 (m, 1H, Ar-H). ^13^C NMR (400MHz) δ: 163.35, 141.74, 138.35, 131.85, 131.19, 129.84, 127.46, 122.00, 121.07, 113.35. Molecular Formula: C_15_H_9_BrClN_3_O_2_ (ESI) Calculated= 378.6078, Observed= 378.6098.


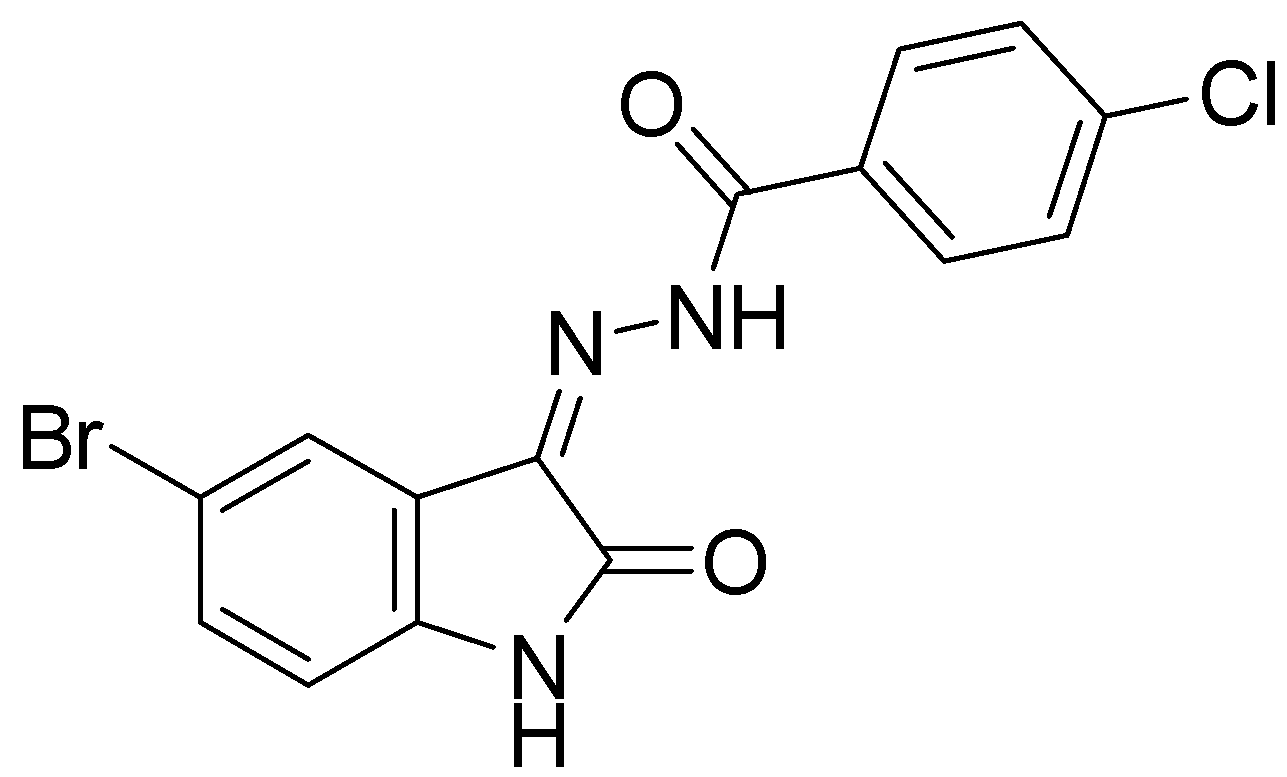


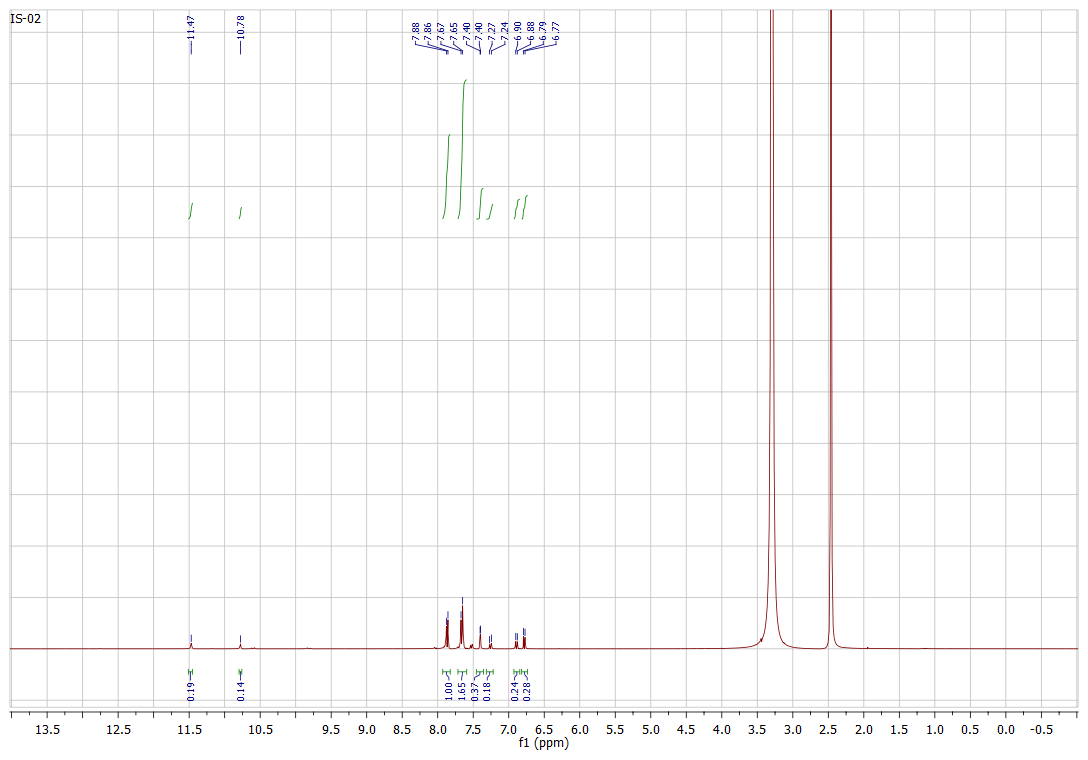


**Figure S4.** ^1^H NMR of **IS2** compound.


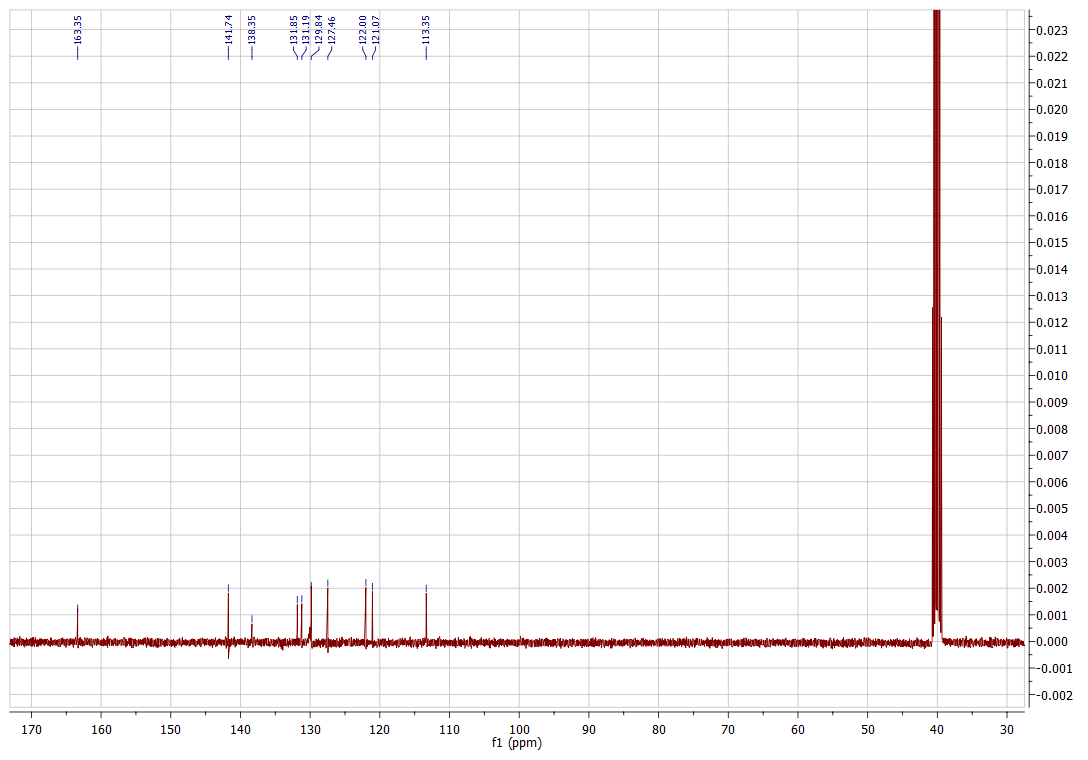


**Figure S5.** ^13^C NMR of **IS2** compound.


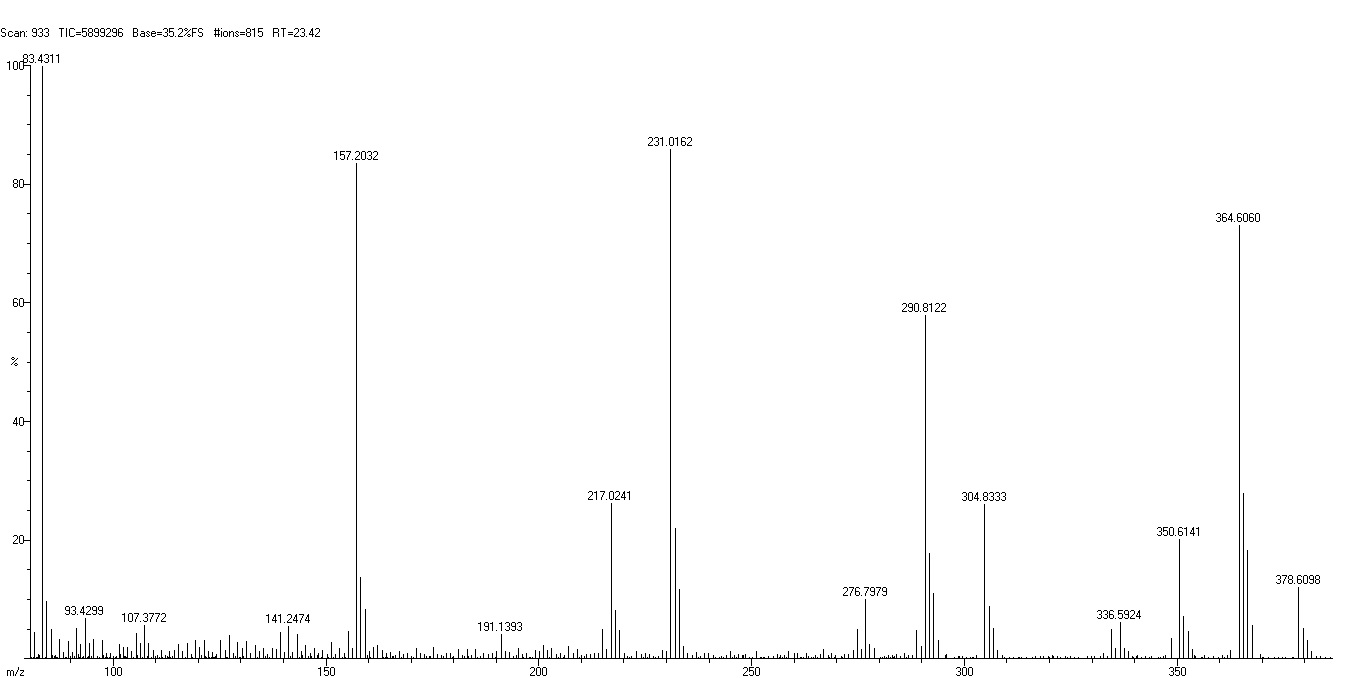


**Figure S6.** MASS of **IS2** compound.

***(Z)-4-bromo-N'-(5-bromo-2-oxoindolin-3-ylidene)benzohydrazide (IS3)***: Yield 82%. NMR (400 MHz, ) δ 11.47 (s, 1H, NH), 7.80 (m, 4H, Ar-H), 7.69-7.63 (m, 1H, Ar-H), 7.54 (dd, 1H, Ar-H), 6.90 (m, 1H, Ar-H).  ^13^C NMR (400 MHz) δ: 162.95, 142.11, 138.03, 134.64, 129.74, 125.02, 123.84, 122.42, 120.20, 115.03, 113.80, 112.43. Molecular Formula: C_15_H_9_Br_2_N_3_O_2_ (ESI) Calculated= 423.0588, Observed= 423.0598.


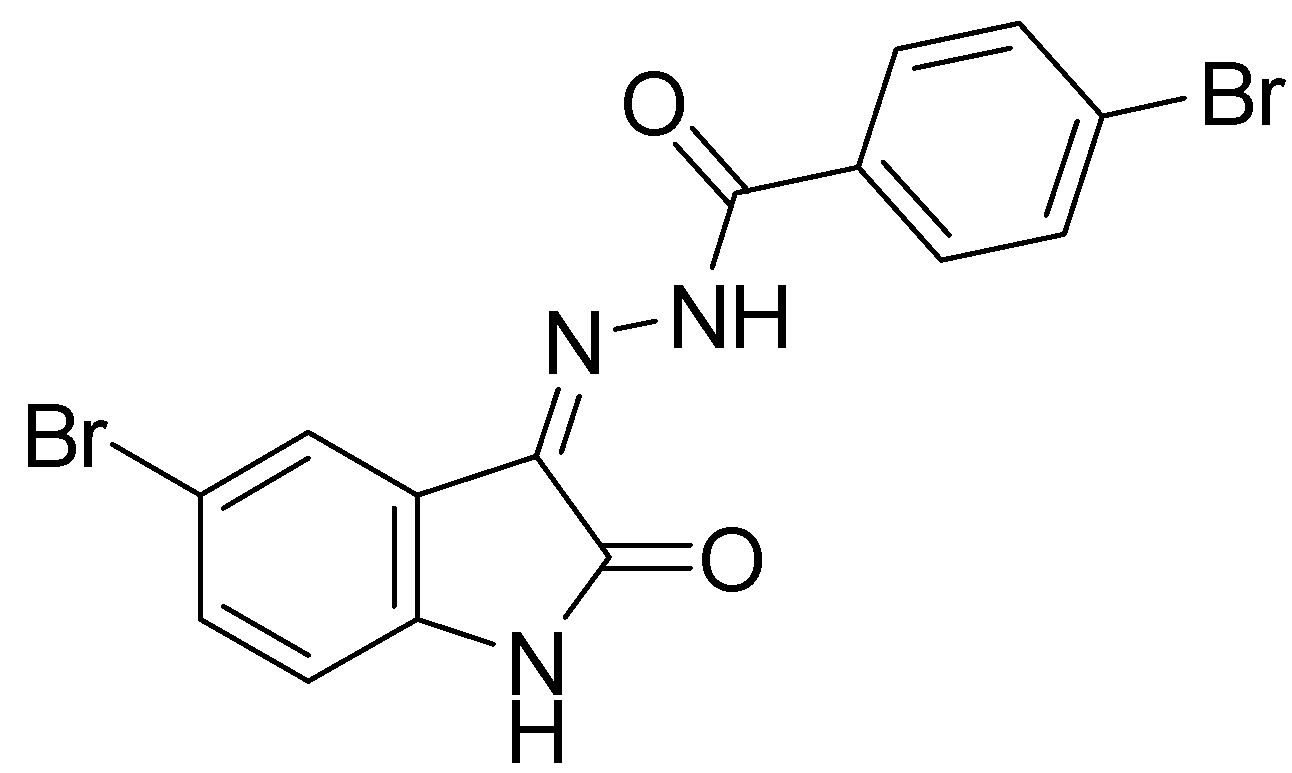


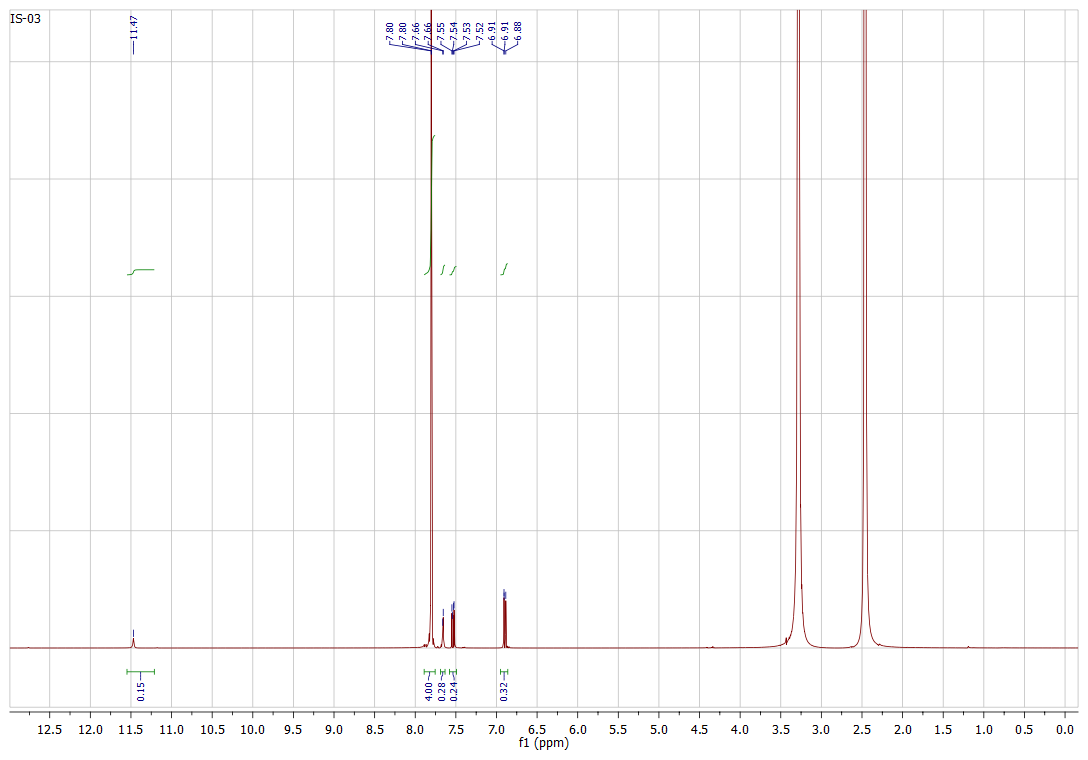


**Figure S7.** ^1^H NMR of **IS3** compound.


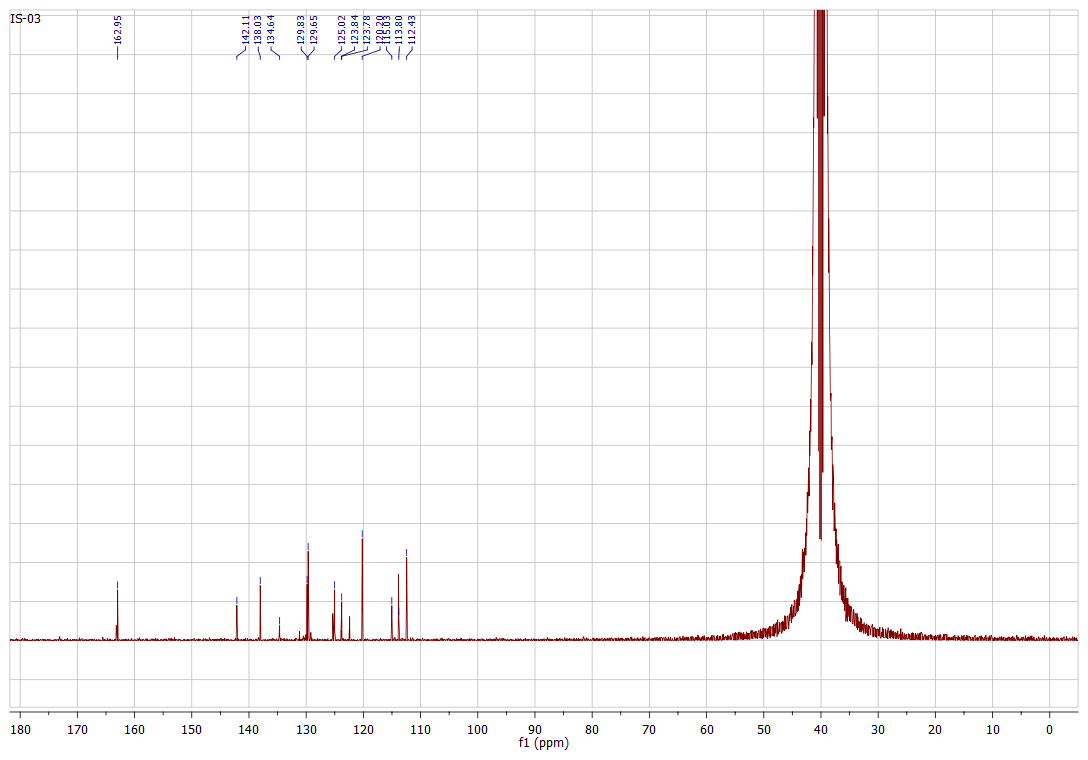


**Figure S8.** ^13^C NMR of **IS3** compound.


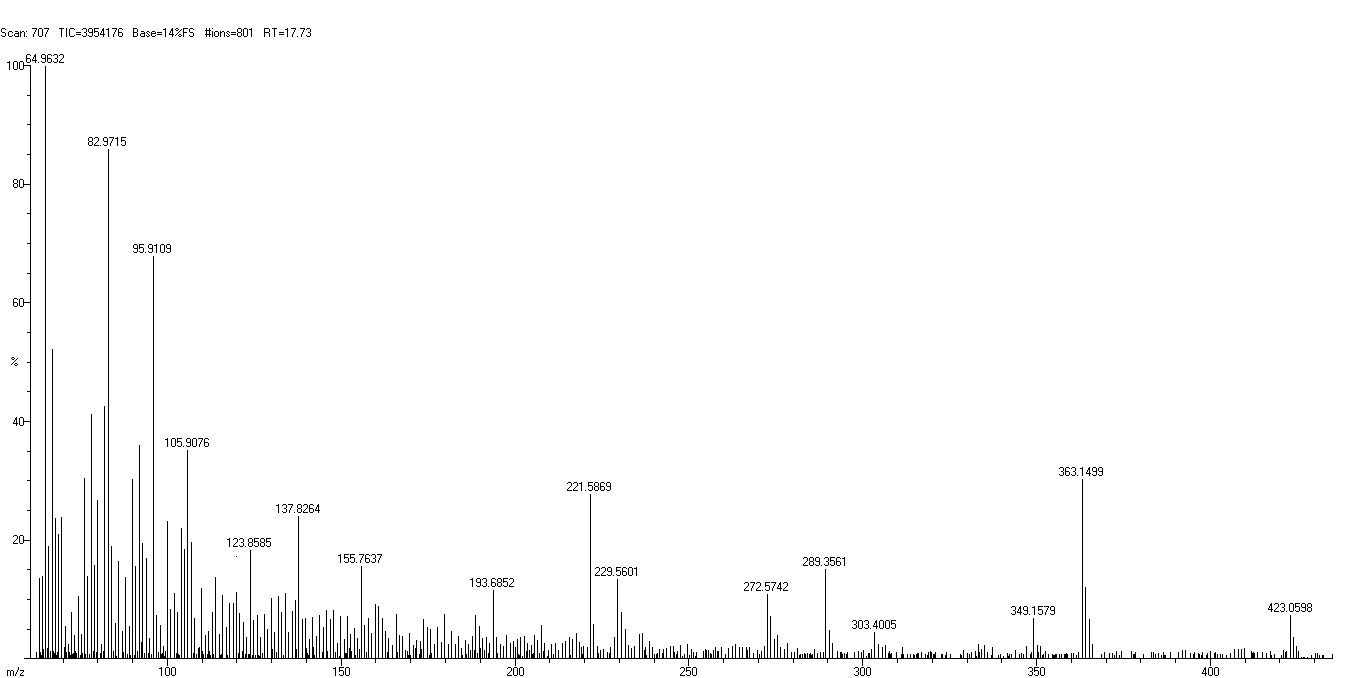


**Figure S9.** MASS of **IS3** compound.

***(Z)-N'-(5-bromo-2-oxoindolin-3-ylidene)-4-fluorobenzohydrazide (IS4)***: Yield 78%. NMR (400 MHz,) δ 10.99 (1H, NH), 7.84 (m, 2H, Ar-H), 7.57 (m, 1H, Ar-H), 7.48-7.38 (m, 1H, Ar-H), 7.13 (m, 1H, Ar-H), 7.07-6.89 (m, 2H, Ar-H). ^13^C NMR (400 MHz) δ: 163.20, 142.13, 134.65, 132.79, 131.55, 123.80, 122.41, 119.29, 115.02, 113.84. Molecular Formula: C_15_H_9_BrFN_3_O_2_ (ESI) Calculated= 362.1532, Observed= 362.1599.


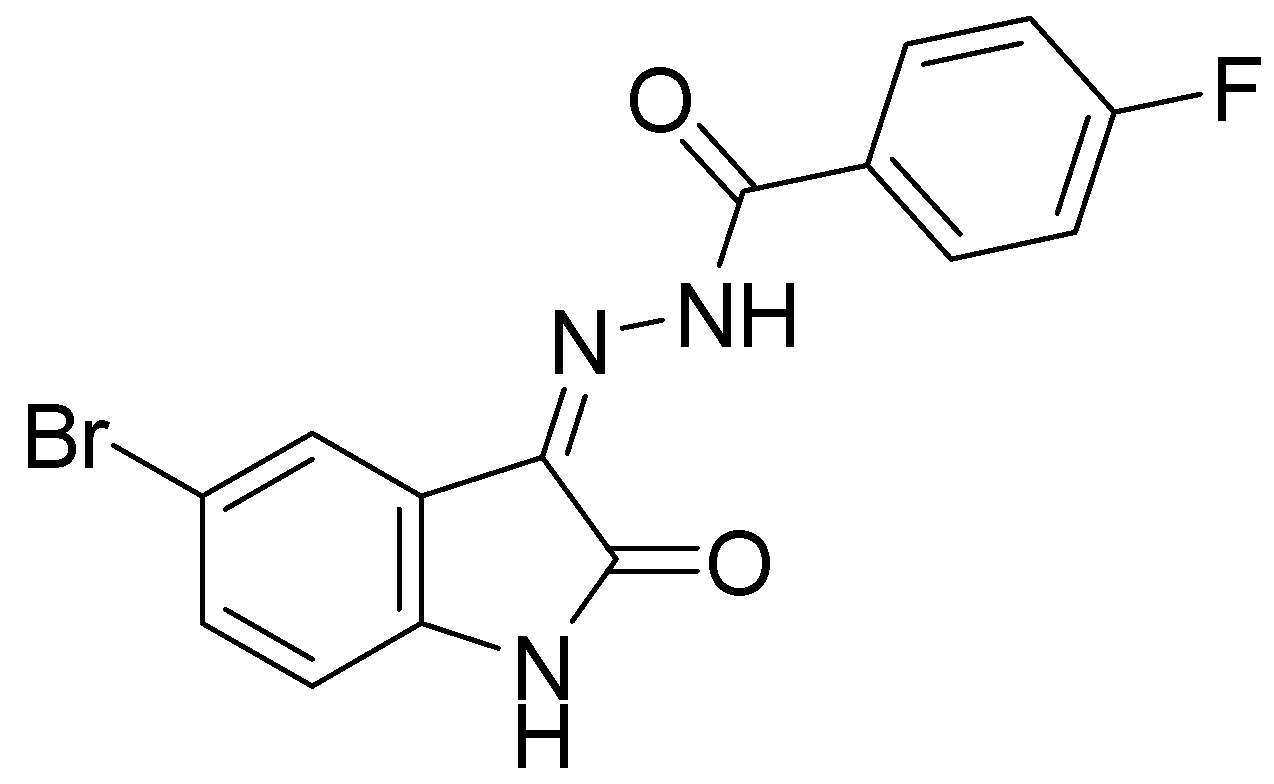


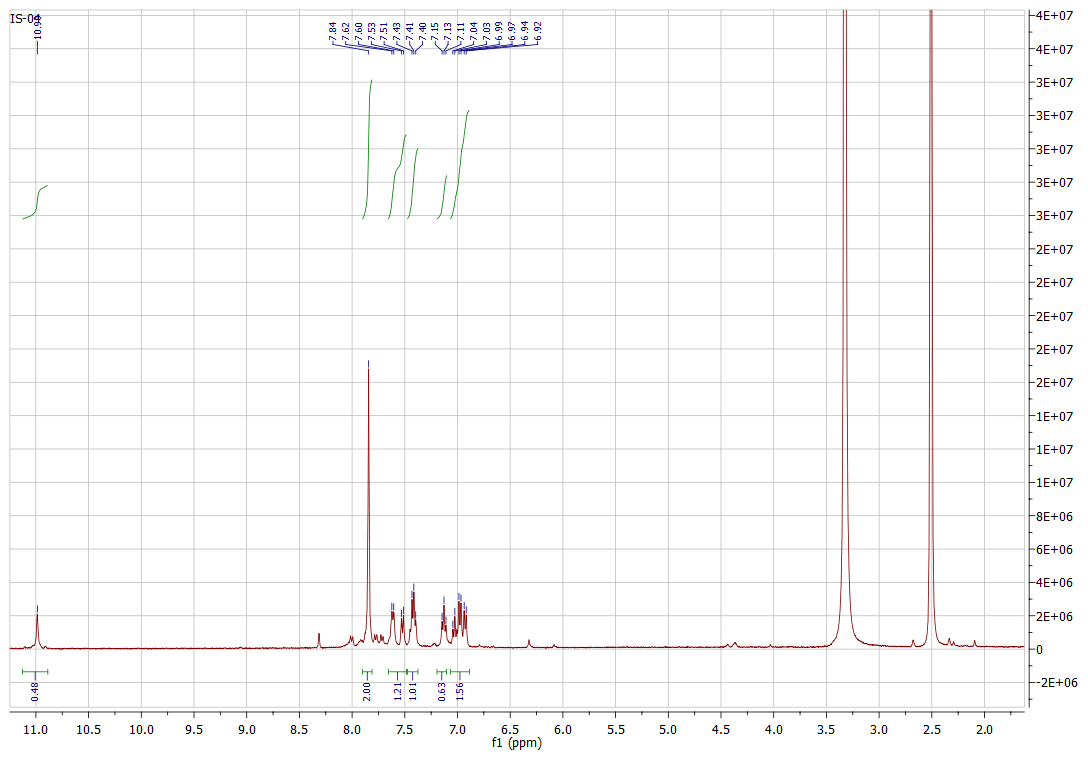


**Figure S10.** ^1^H NMR of **IS4** compound.


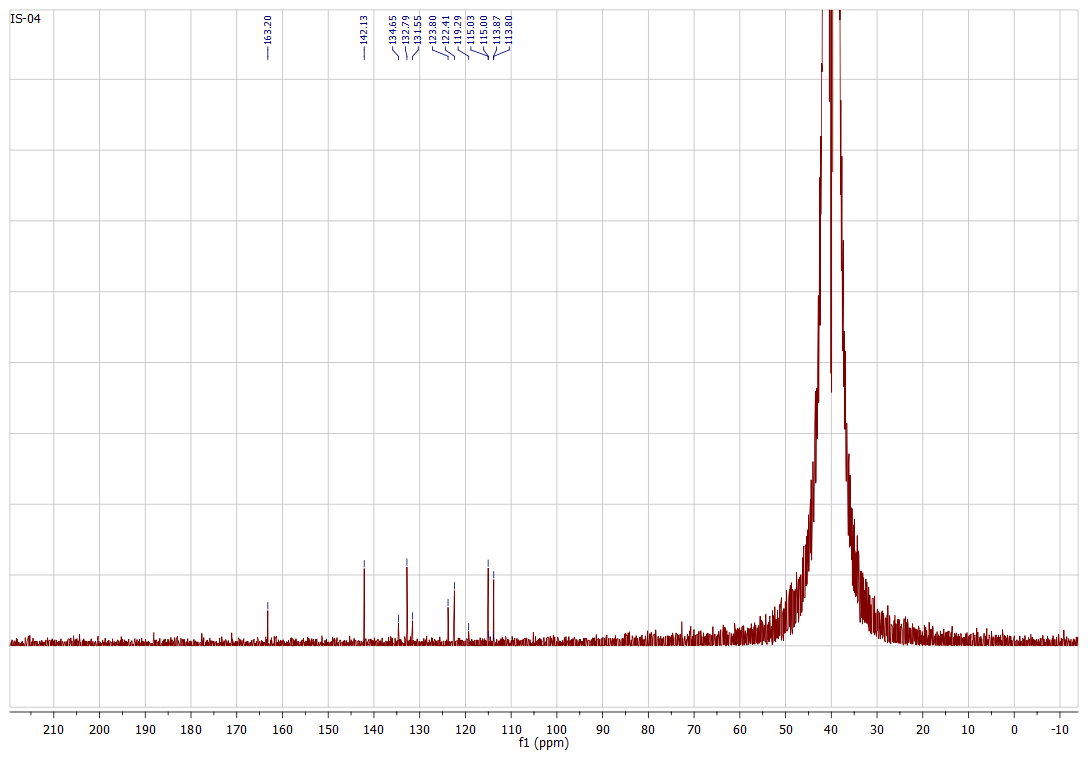


**Figure S11.** ^13^C NMR of **IS4** compound.
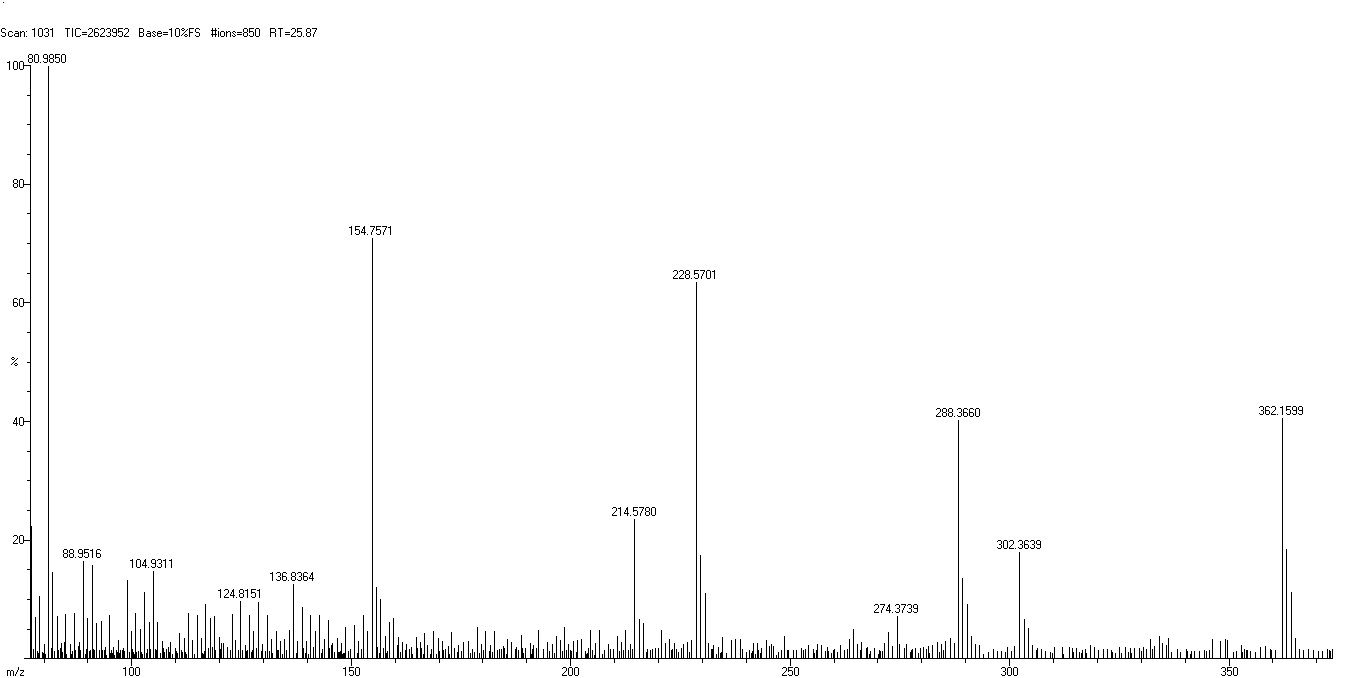


**Figure S12.** MASS of **IS4** compound.

***(Z)-N'-(2-oxoindolin-3-ylidene)benzohydrazide (IS5):*** Yield 96%. NMR (400 MHz) δ 11.32 (1H, NH), 7.86 (m, 2H, Ar-H), 7.65 (m, 1H, Ar-H), 7.61-7.53 (m, 3H, Ar-H), 7.36 (d, 1H, Ar-H), 7.06 – 7.10 (m, 1H, Ar-H), 6.93 (d, 1H, Ar-H). ^13^C NMR (400 MHz) δ: 163.59, 142.98, 133.44, 132.48, 129.72, 127.93, 123.35, 121.54, 120.31, 111.79. Molecular Formula: C_15_H_11_N_3_O_2_ (ESI) Calculated= 265.2667, Observed= 265.2699.


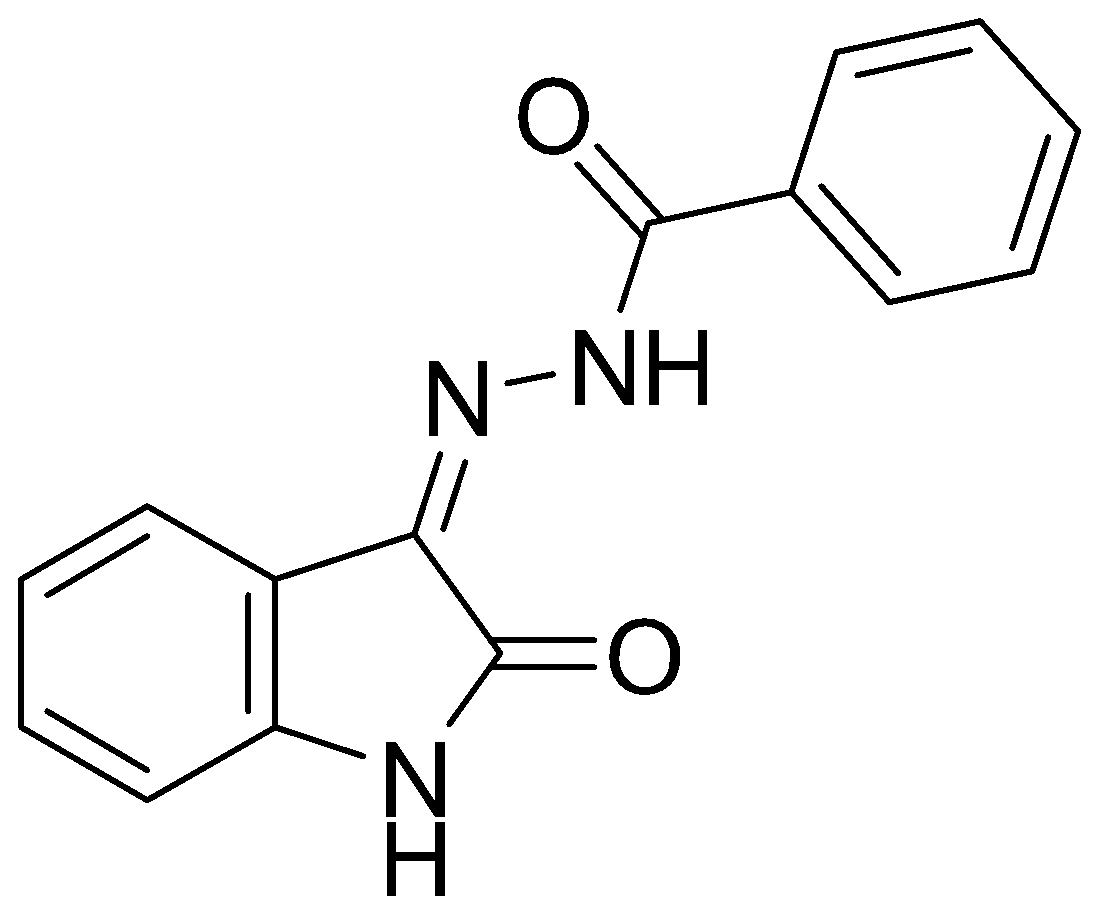

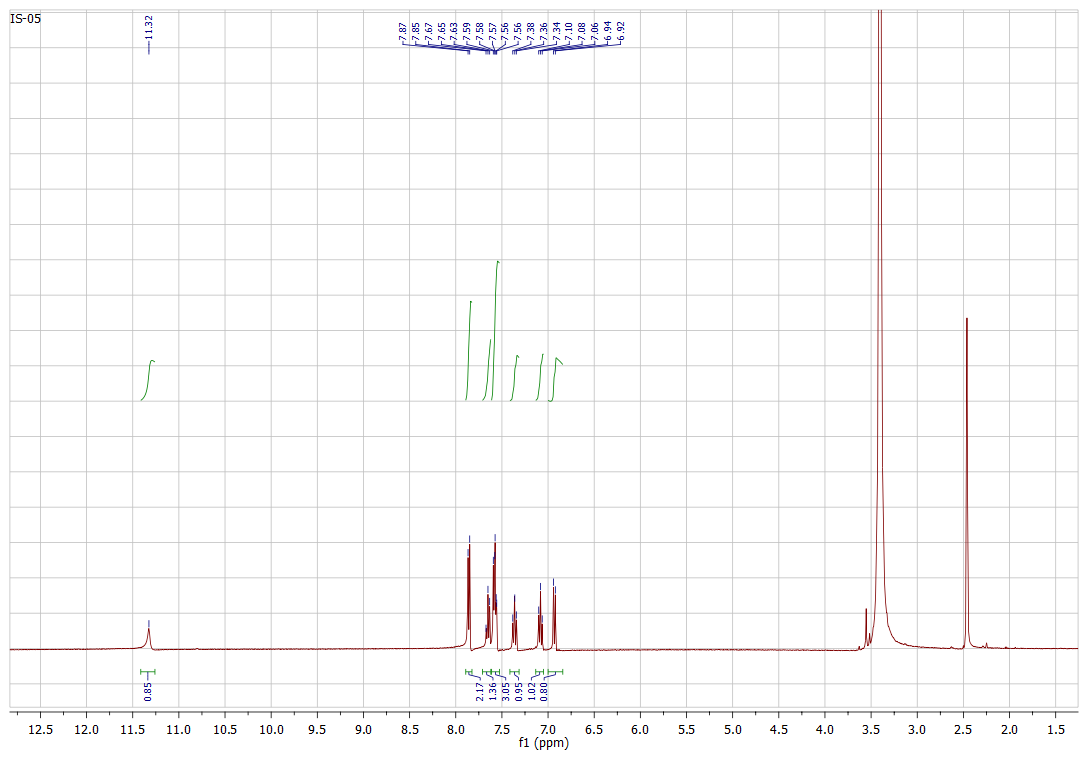


**Figure S13.** ^1^H NMR of **IS5** compound.


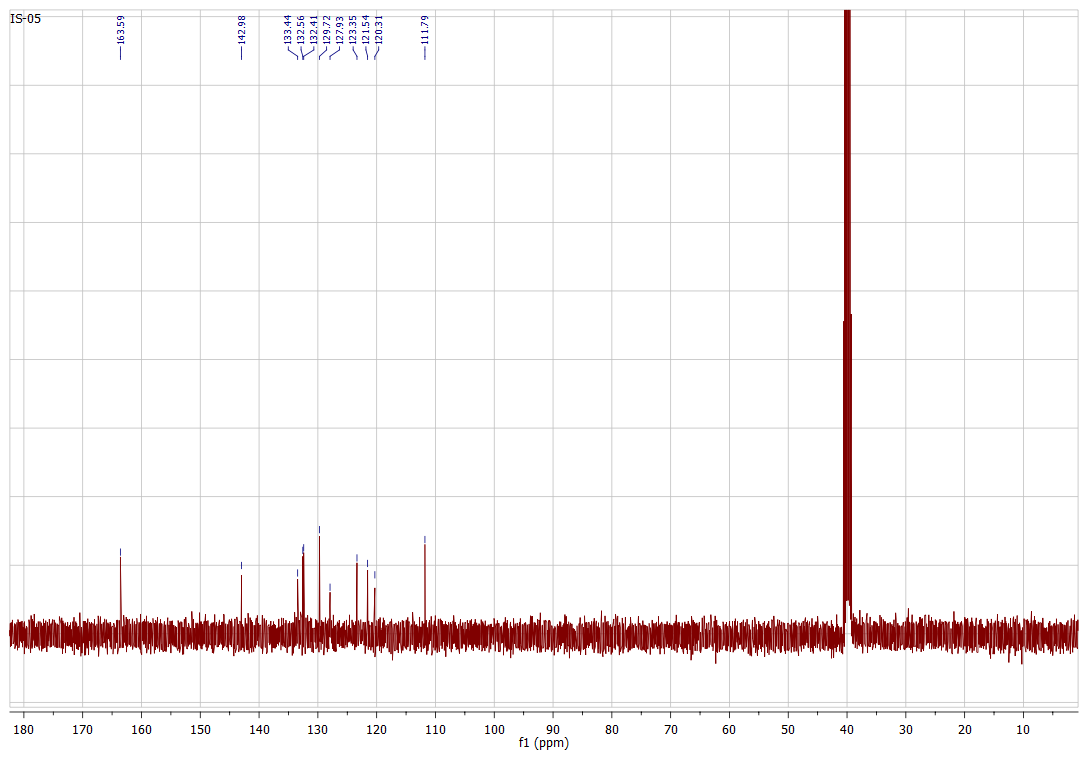


**Figure S14.** ^13^C NMR of **IS5** compound.


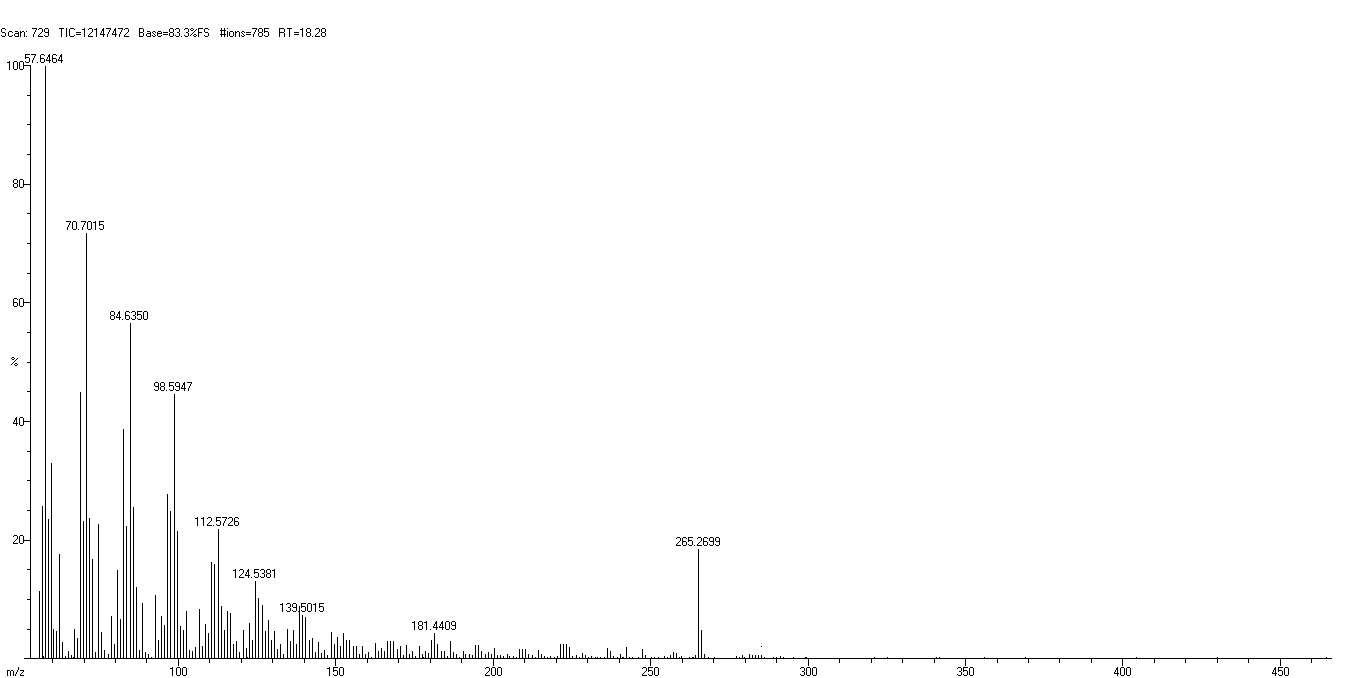


**Figure S15.** MASS of **IS5** compound.

**(Z)-4-chloro-N'-(2-oxoindolin-3-ylidene) benzohydrazide (IS6)**: Yield 91%. ^1^H NMR (400 MHz, DMSO) δ: 13.89 (s, 1H, NH), 11.38 (s, 1H, NH), 8.09-7.80 (d, 2H, Ar-H), 7.80–7.51 (m, 3H, Ar-H), 7.51-7.40 (m, 1H, Ar-H), 7.15-6.77 (m, 2H, Ar-H). ^13^C NMR (400 MHz) δ: 163.35, 141.74, 138.35, 131.85, 131.19, 129.84, 127.46, 122.00, 121.07, 113.35. Molecular Formula: C_15_H_10_ClN_3_O_2_ (ESI) Calculated= 299.7118, Observed= 299.7098.


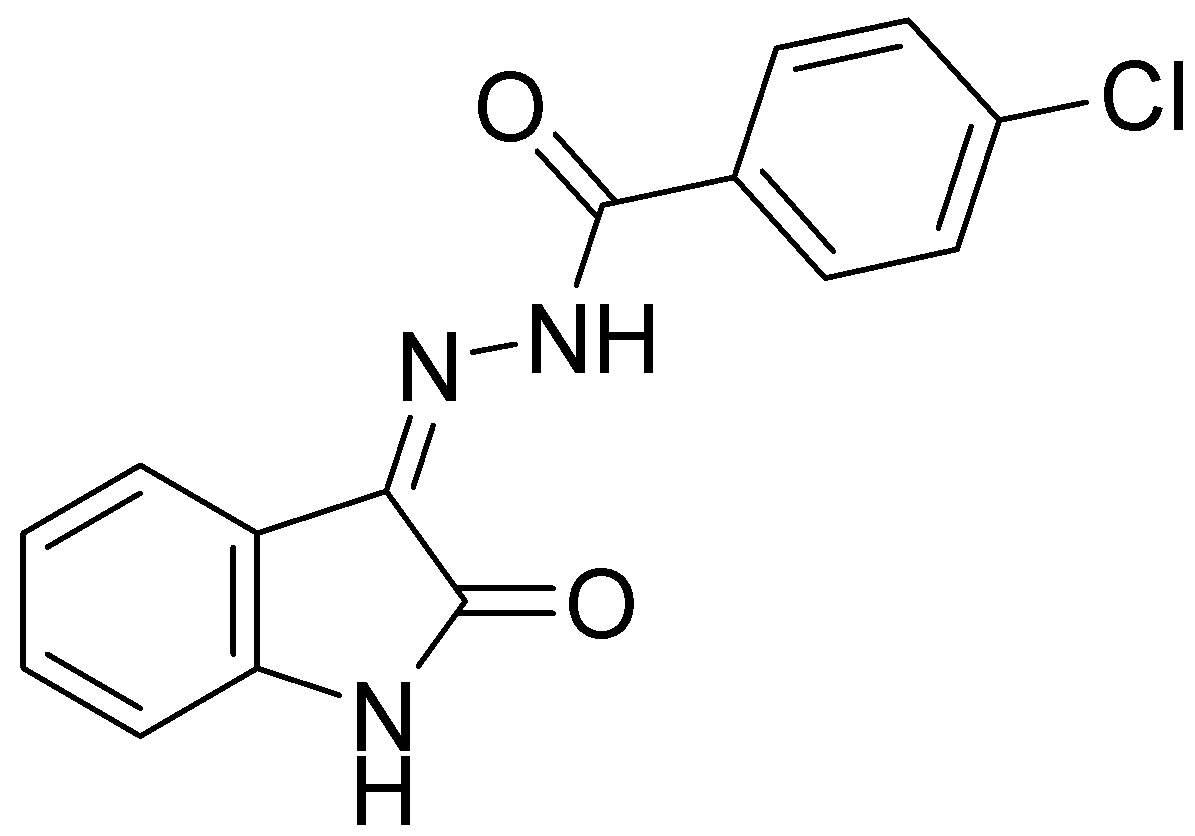


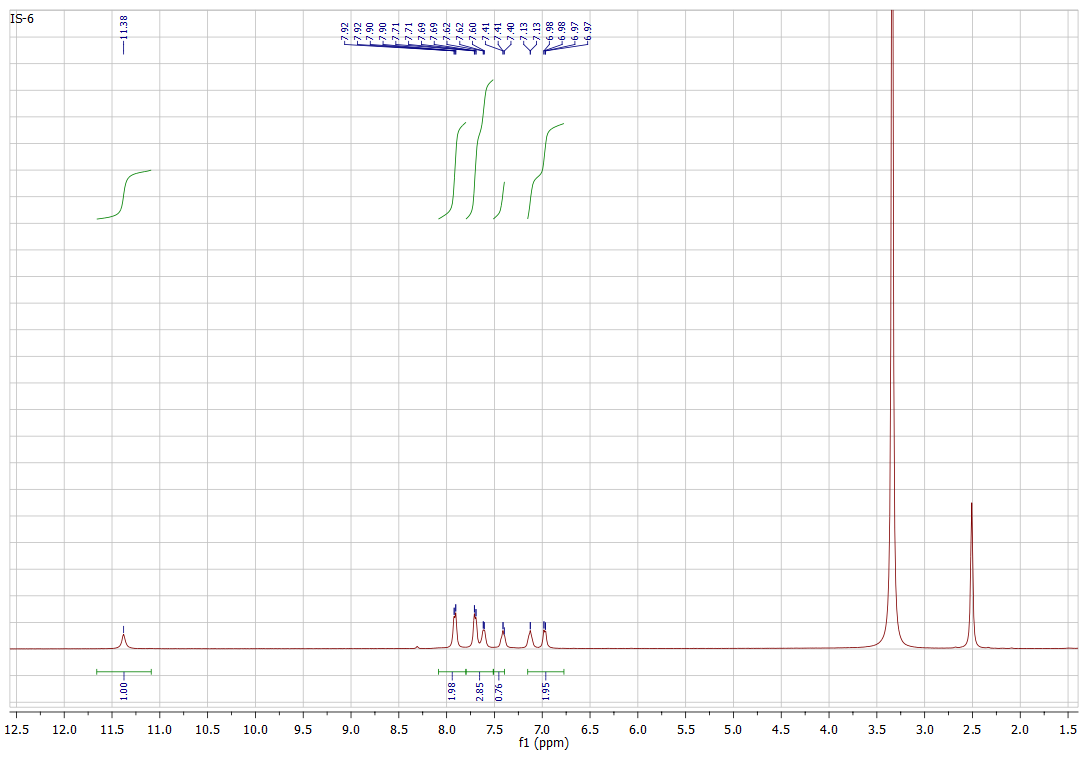


**Figure S16.** ^1^H NMR of **IS6** compound.


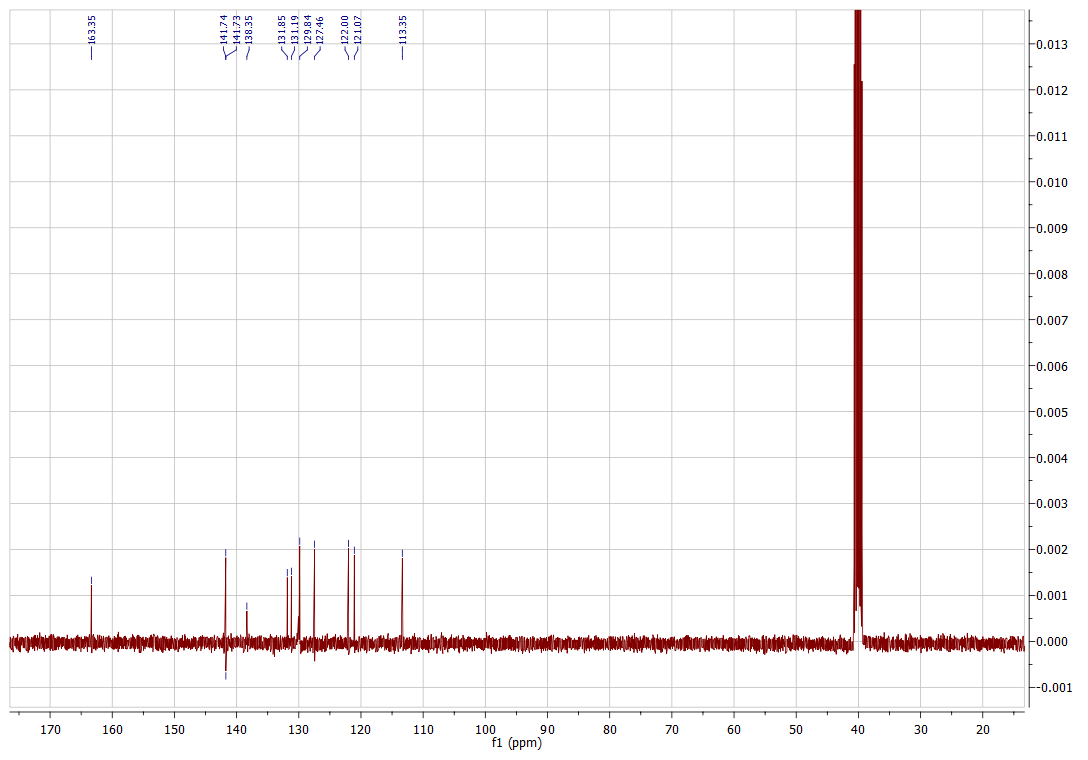


**Figure S17.** ^13^C NMR of **IS6** compound.


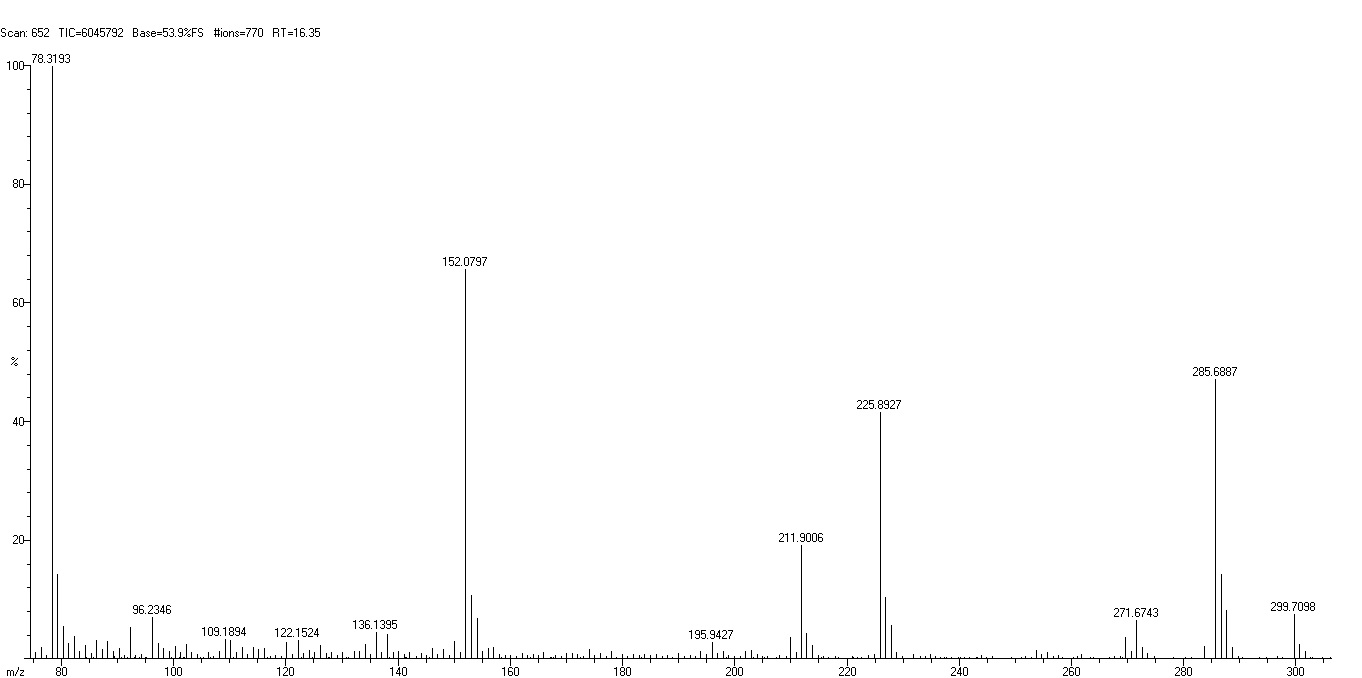


**Figure S18.** MASS of **IS6** compound.

**(Z)-4-bromo-N'-(2-oxoindolin-3-ylidene) benzohydrazide (IS7)**: Yield 88%. ^1^H NMR (400 MHz, DMSO) δ: 13.89 (s, 1H, NH), 11.24 (s, 1H, NH), 7.91 (d, 2H, Ar-H), 7.68 (m, 2H, Ar-H), 7.62 (d, 1H, Ar-H), 7.41 (d, 1H, Ar-H), 6.97 (d, 1H, Ar-H). ^13^C NMR (400 MHz) δ: 163.20, 142.13, 134.65, 132.79, 131.55, 123.80, 122.41, 115.03, 113.80. Molecular Formula: C_15_H_10_BrN_3_O_2_ (ESI) Calculated= 344.1628, Observed= 344.1699.


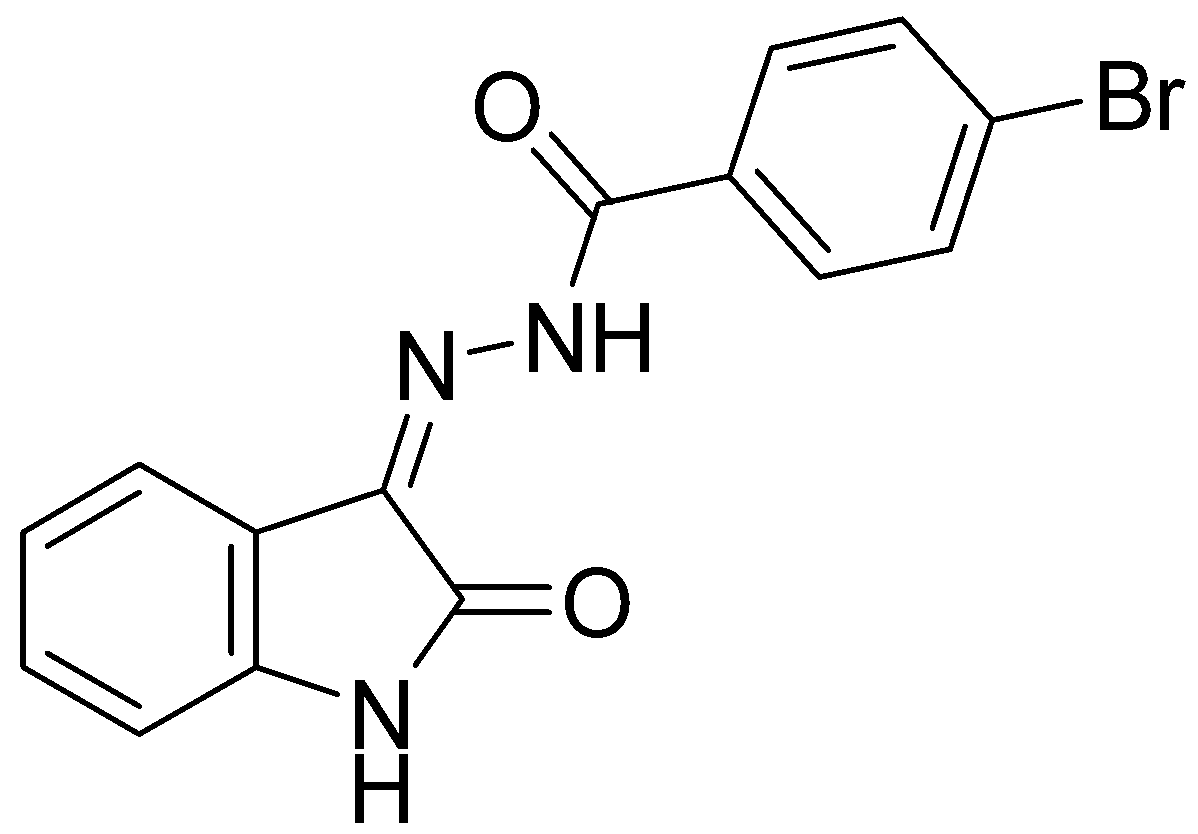


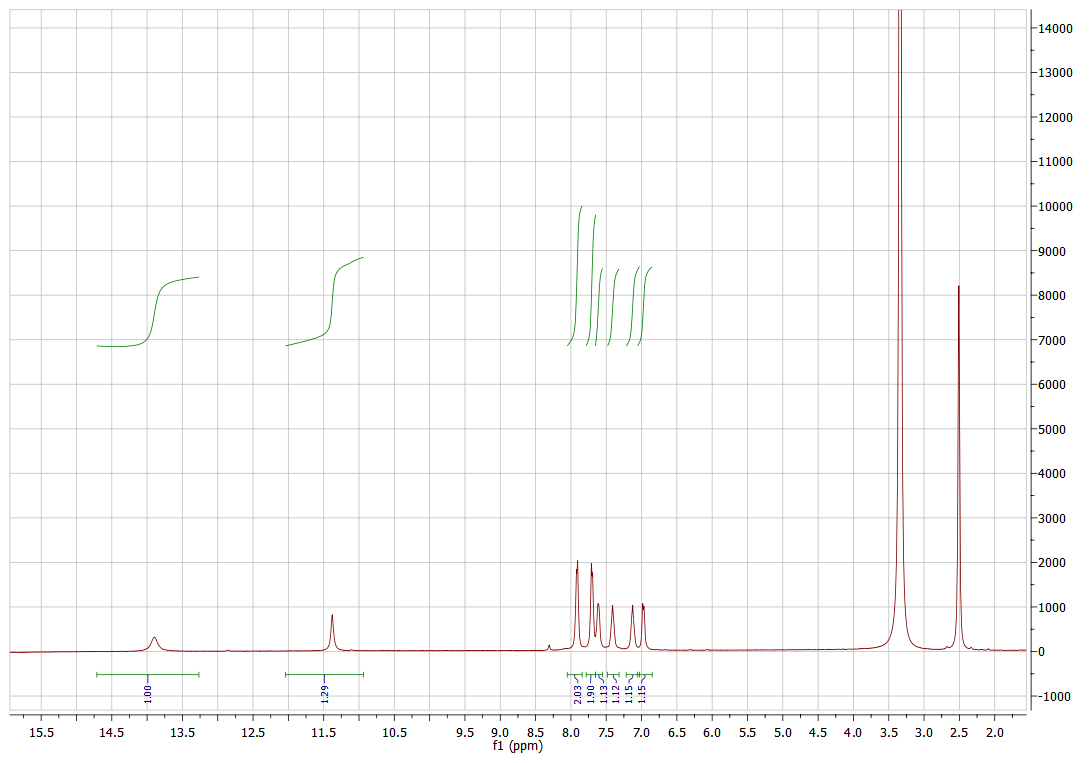


**Figure S19.** ^1^H NMR of **IS7** compound.


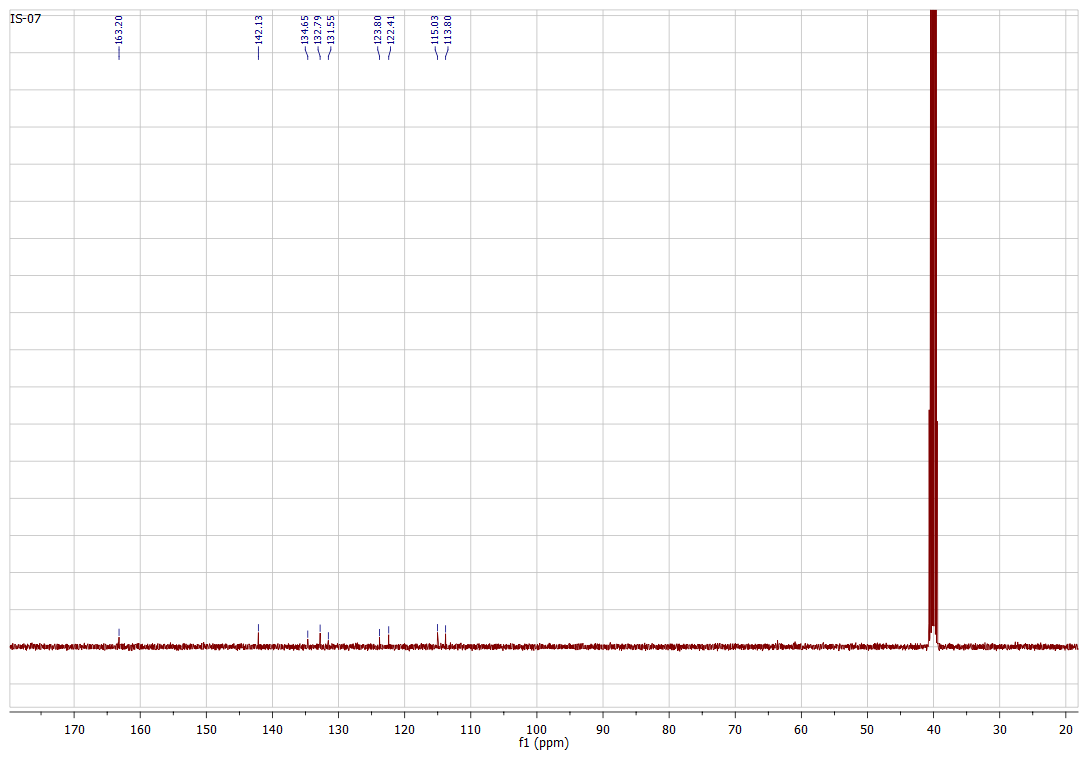


**Figure S20.** ^13^C NMR of **IS7** compound.


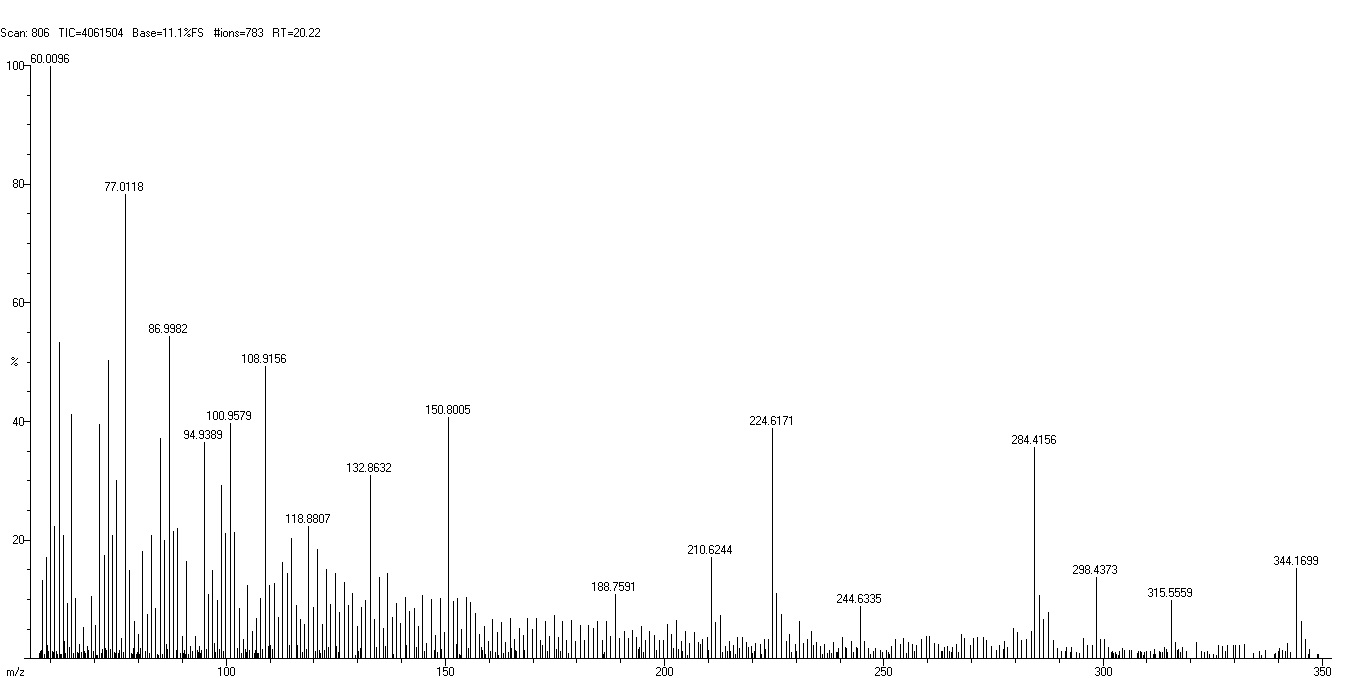


**Figure S21.** MASS of **IS7** compound.

***(Z)-4-fluoro-N'-(2-oxoindolin-3-ylidene)benzohydrazide (IS8)***: Yield 87%. ^1^H NMR (400 MHz, DMSO) δ 13.89 (s, 1H, NH), 11.38 (s, 1H, NH), 7.82 (d, *2*H, Ar-H), 7.61 (d, 1H, Ar-H), 7.52 (d, 1H, Ar-H), 7.42 (dd,1H, Ar-H), 7.13 (t, 1H, Ar-H), 7.06 – 6.90 (m, 2H, Ar-H). ^13^C NMR (400 MHz) δ: 163.40, 141.66, 133.56, 132.38, 131.76, 129.75, 127.44, 122.06, 121.02, 113.33. Molecular Formula: C_15_H_10_FN_3_O_2_ (ESI) Calculated= 283.2572, Observed= 283.2599.


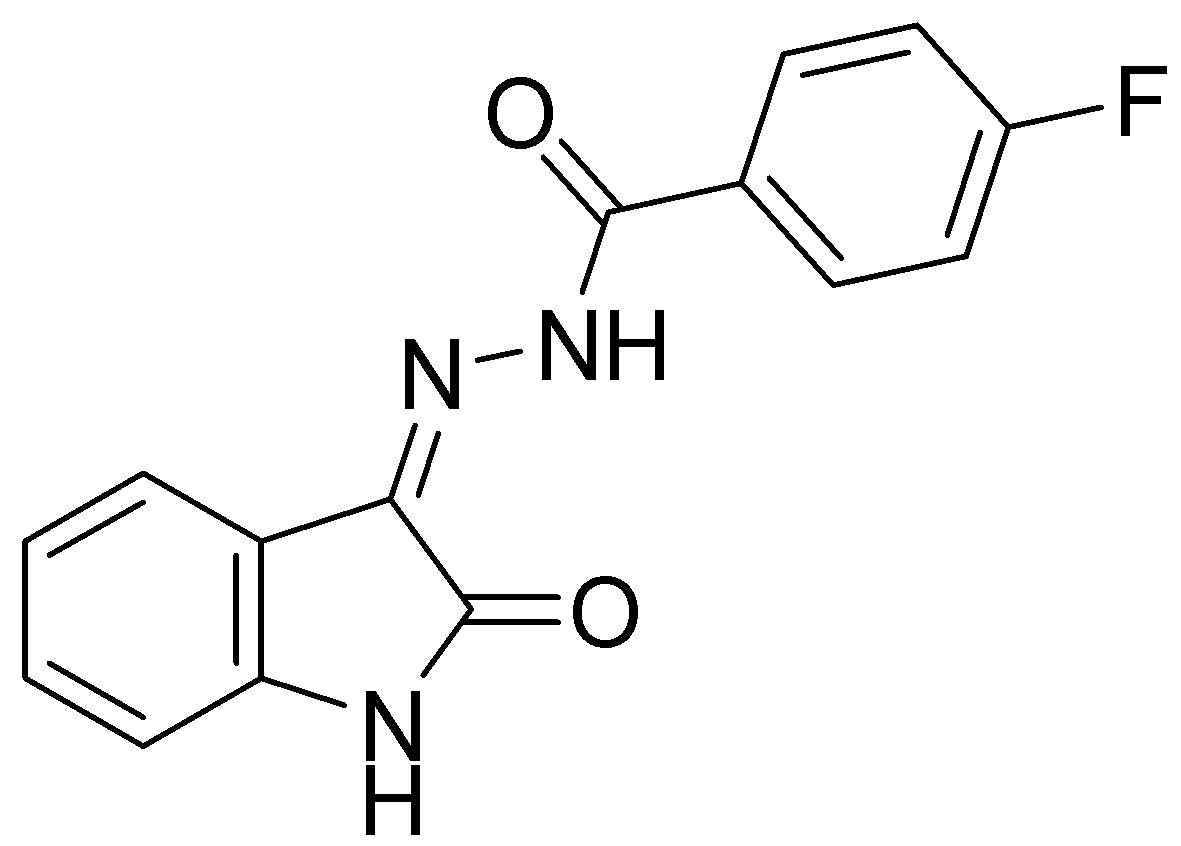


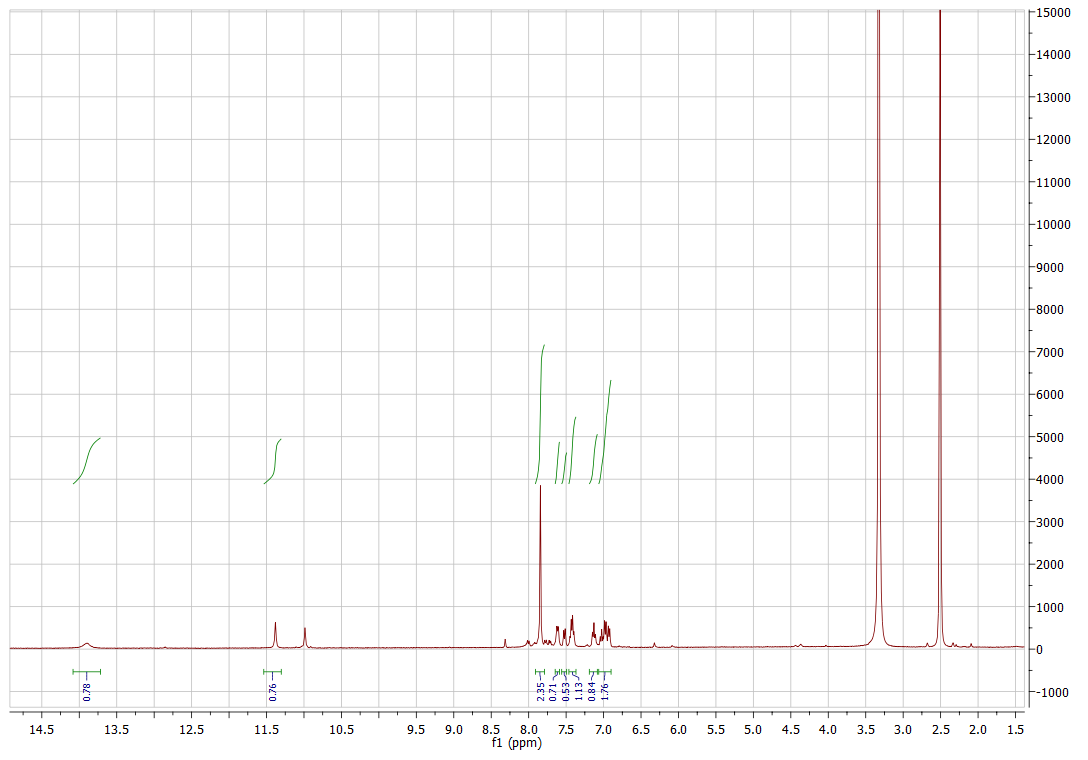


**Figure S22.** ^1^H NMR of **IS8** compound.


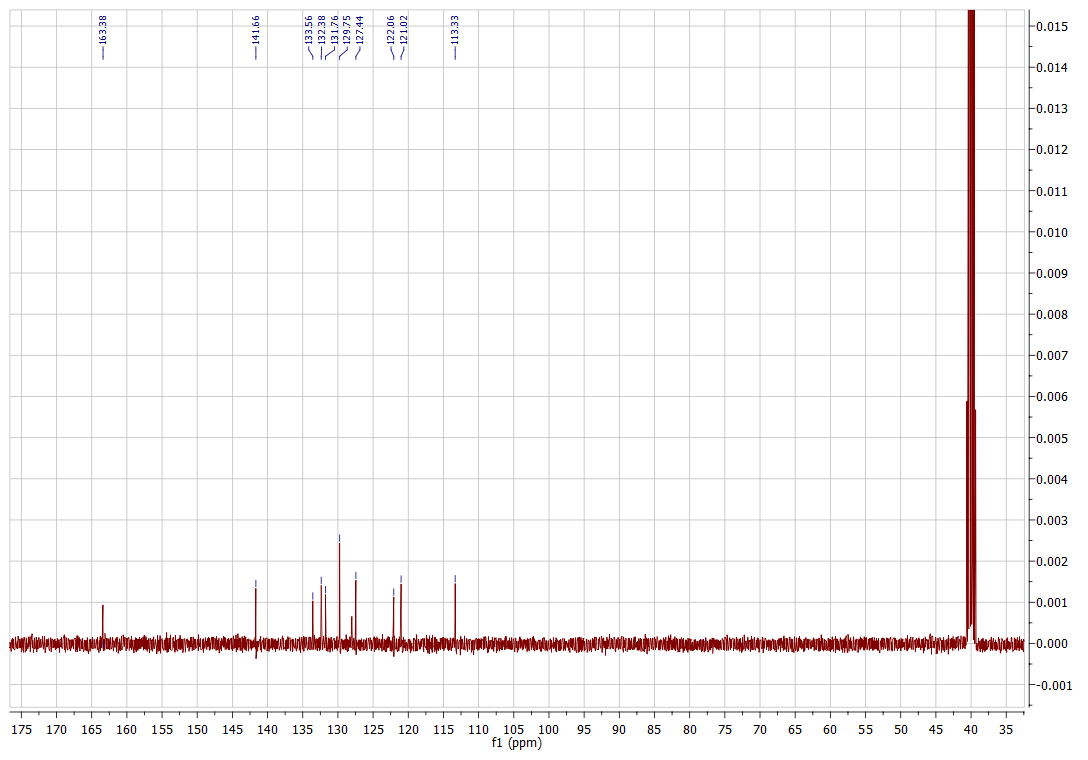


**Figure S23.** ^13^C NMR of **IS8** compound.


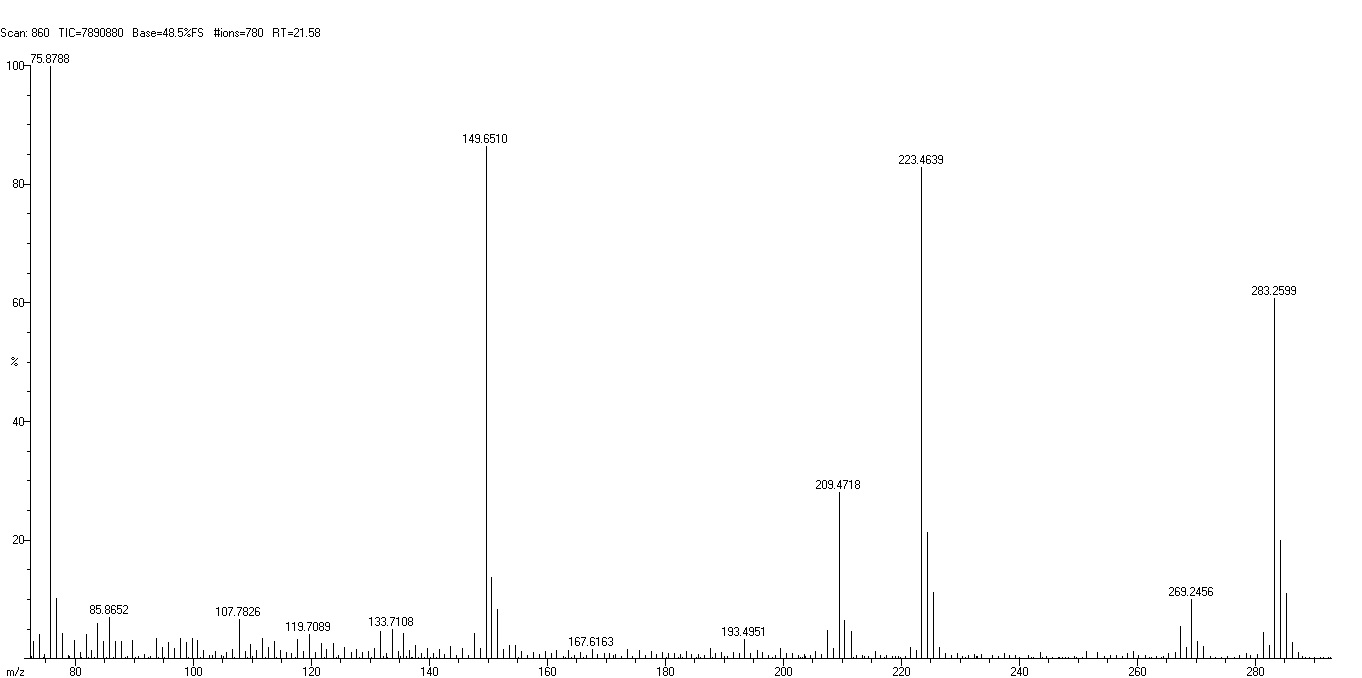


**Figure S24.** MASS of **IS8** compound.

***(Z)-N'-(5-fluoro-2-oxoindolin-3-ylidene)benzohydrazide (IS9***): Yield 82%. NMR (400 MHz, ) δ 11.37 (1H, NH), 7.93 – 7.84 (m, 2H, Ar-H), 7.71-7.54 (m, 3H, Ar-H), 7.40 (m, 1H, Ar-H), 7.25-7.16 (m, 1H, Ar-H), 6.93 (m 1H, Ar-H). ^13^C NMR (400 MHz) δ: 163.73, 157.76, 139.25, 133.54, 132.44, 129.76, 128.01, 121.57, 118.87, 112.95, 108.75. Molecular Formula: C_15_H_10_FN_3_O_2_ (ESI) Calculated= 283.2572, Observed= 283.2598.


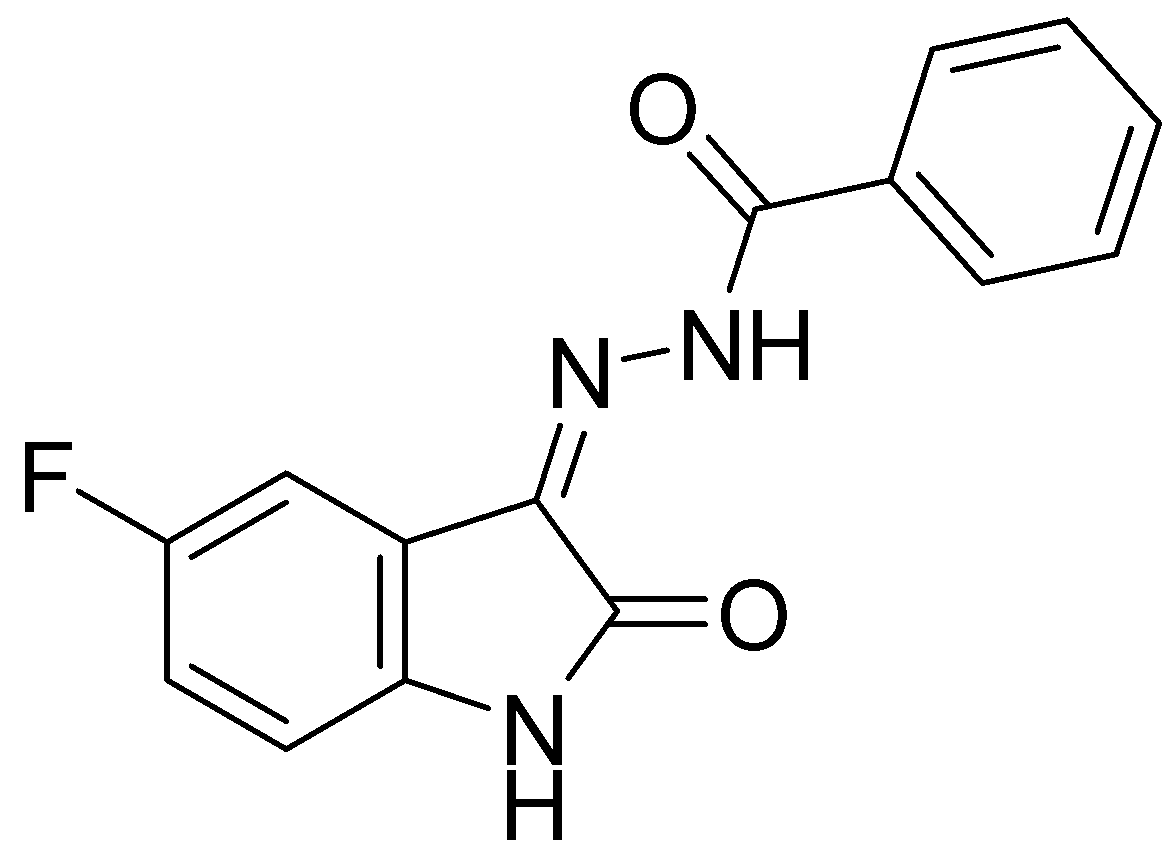


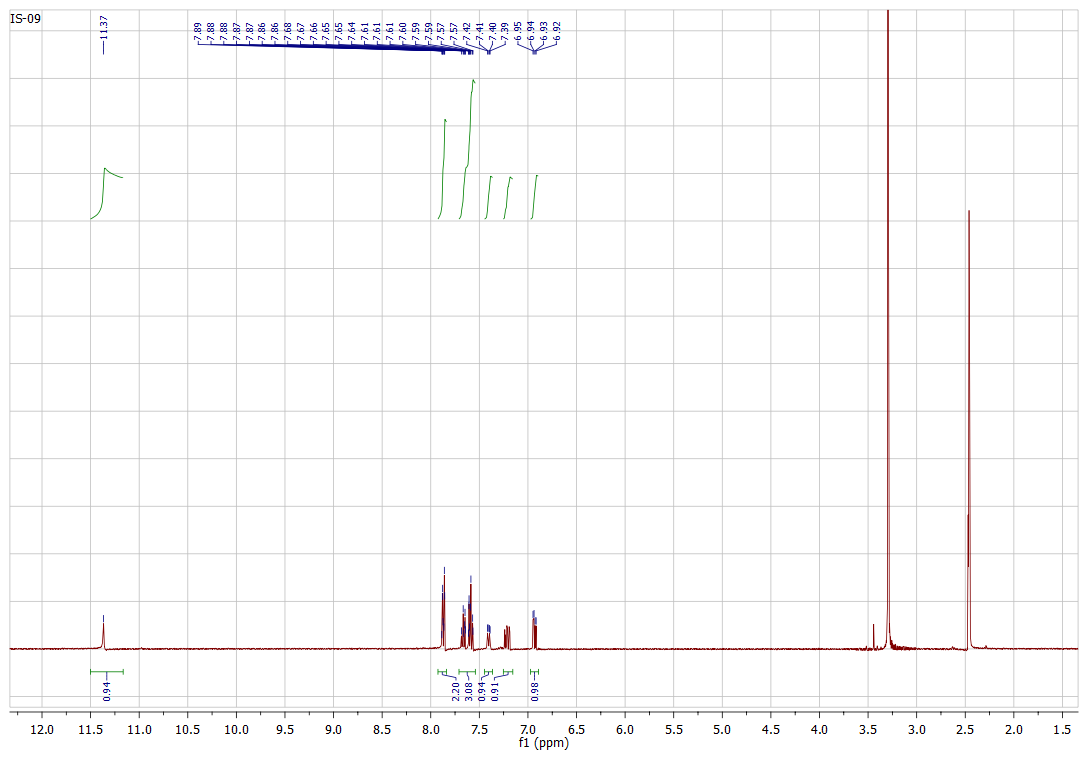


**Figure S25.** ^1^H NMR of **IS9** compound.


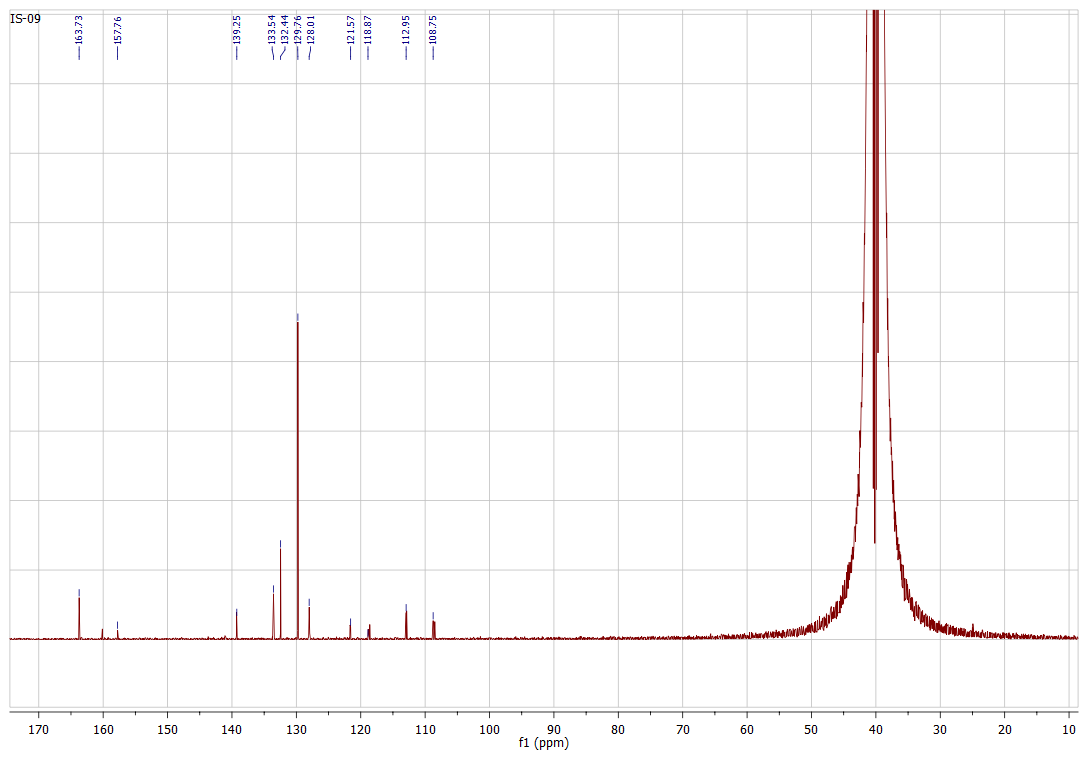


**Figure S26.** ^13^C NMR of **IS9** compound.


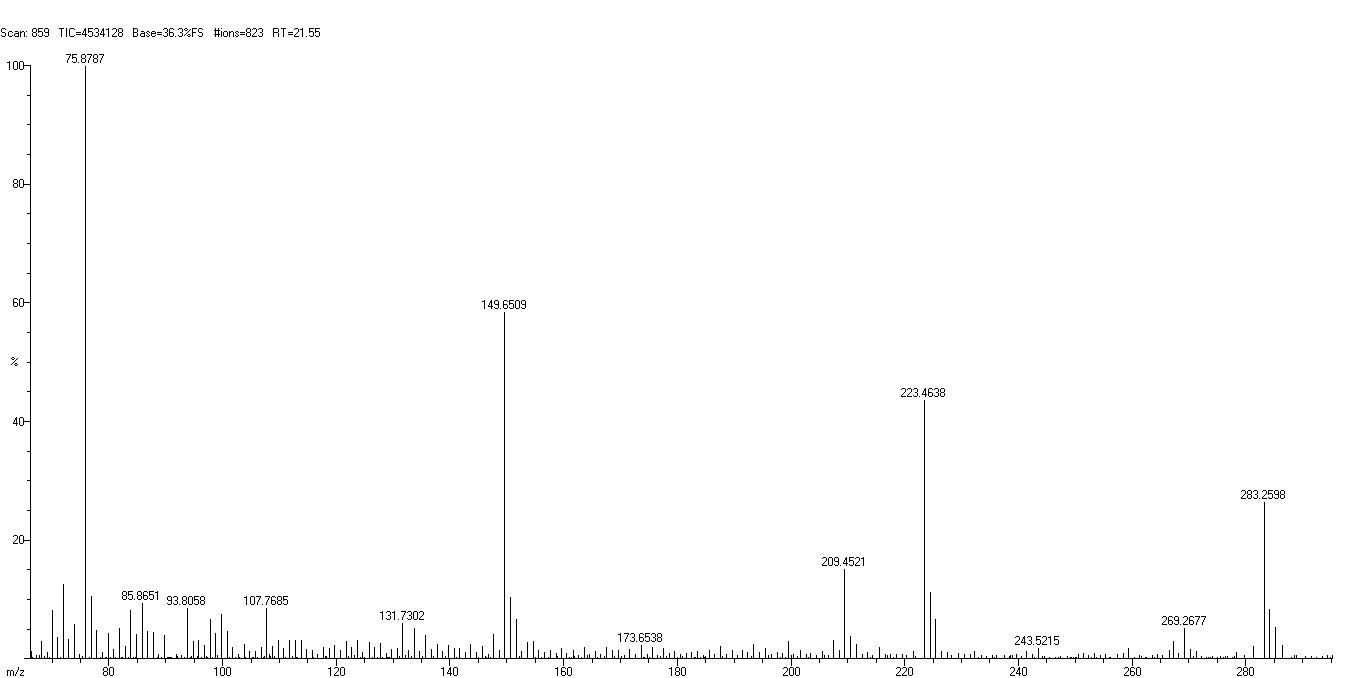


**Figure S27.** MASS of **IS9** compound.

**(Z)-4-chloro-N'-(5-fluoro-2-oxoindolin-3-ylidene)benzohydrazide (IS10):** Yield 81%. NMR (400 MHz, ) δ: 11.46 (s, 1H, NH), 7.88 (1H, Ar-H), 7.86 (1H, Ar-H), 7.67 (1H, Ar-H), 7.66 (1H, Ar-H) 7.54 (1H, Ar-H), 7.40 (1H, Ar-H), 6.94 (1H, Ar-H). ^13^C NMR (400 MHz) δ: 163.36, 141.77, 132.78, 131.85, 131.54, 127.45, 122.00, 121.07, 113.35. Molecular Formula: C_15_H_9_FClN_3_O_2_ (ESI) Calculated= 317.7022, Observed= 317.6998.


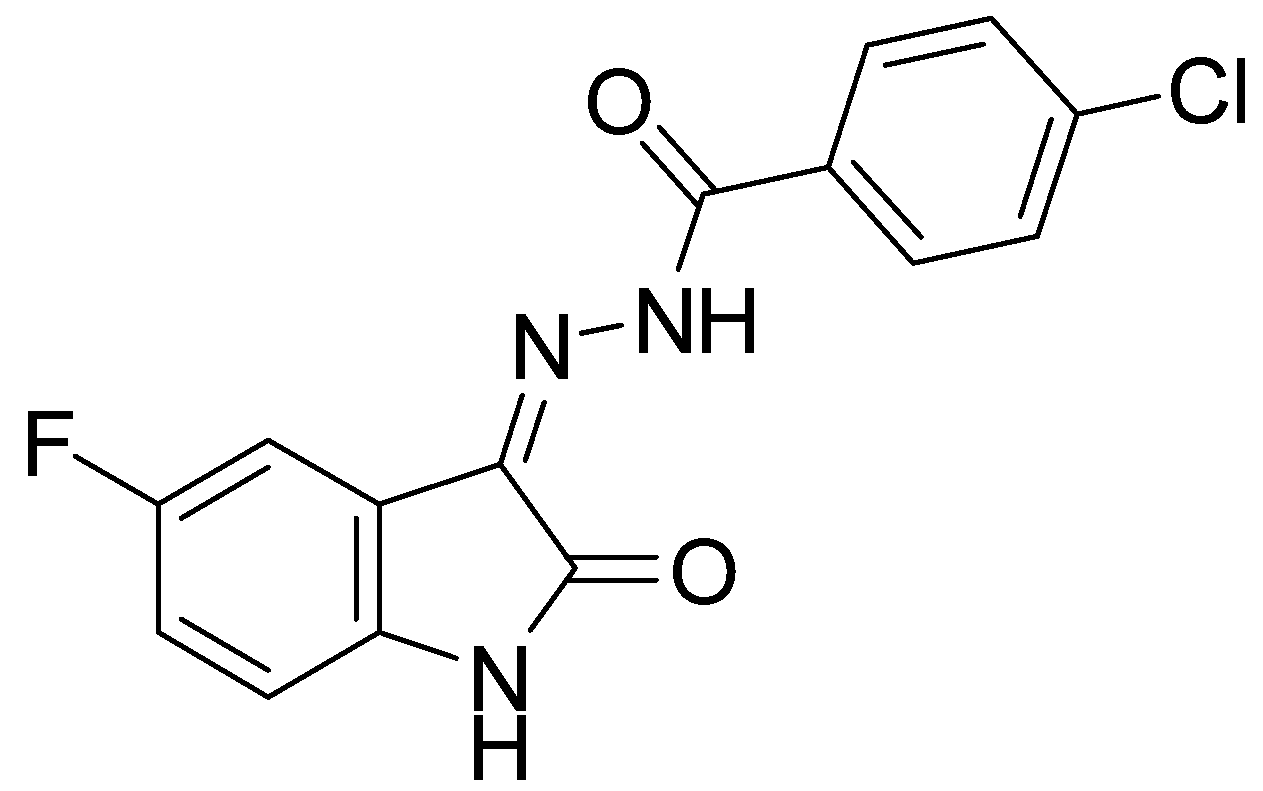


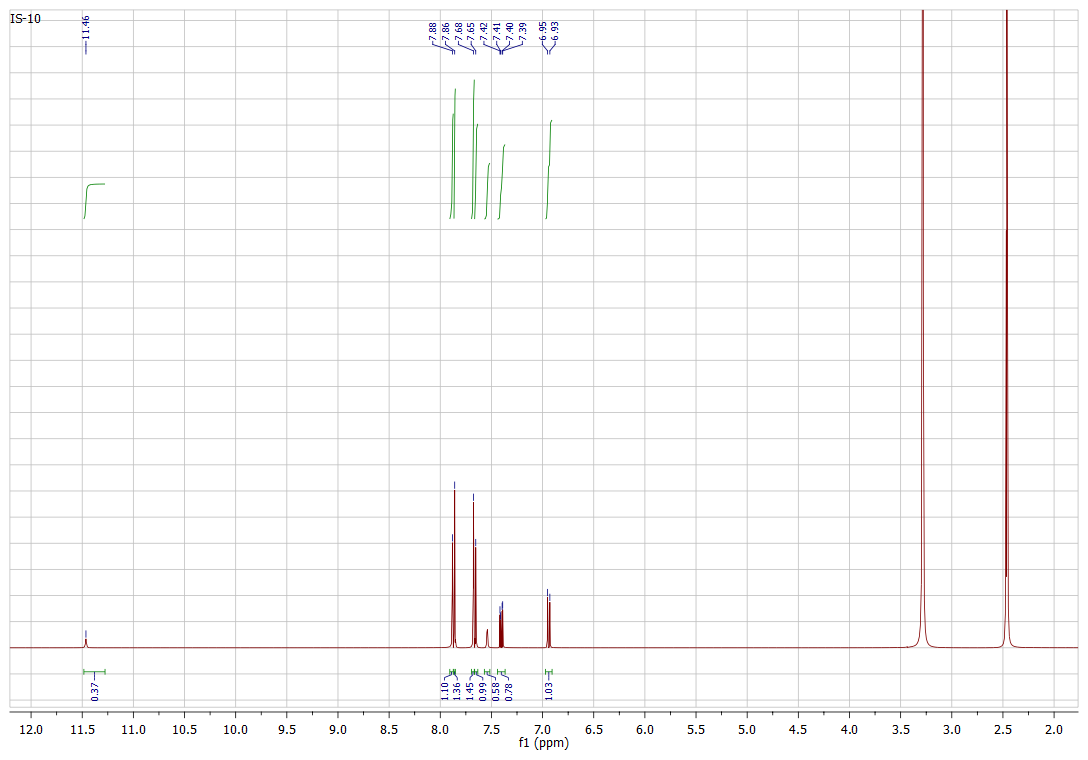


**Figure S28.** ^1^H NMR of **IS10** compound.


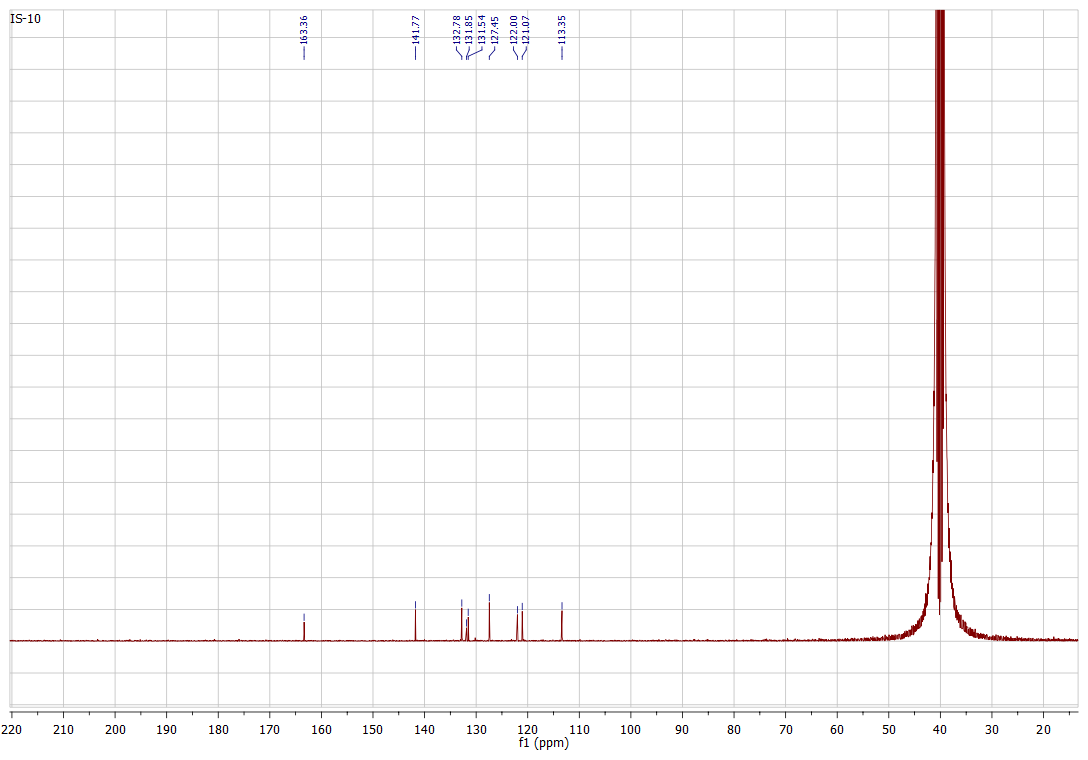


**Figure S29.** ^13^C NMR of **IS10** compound.


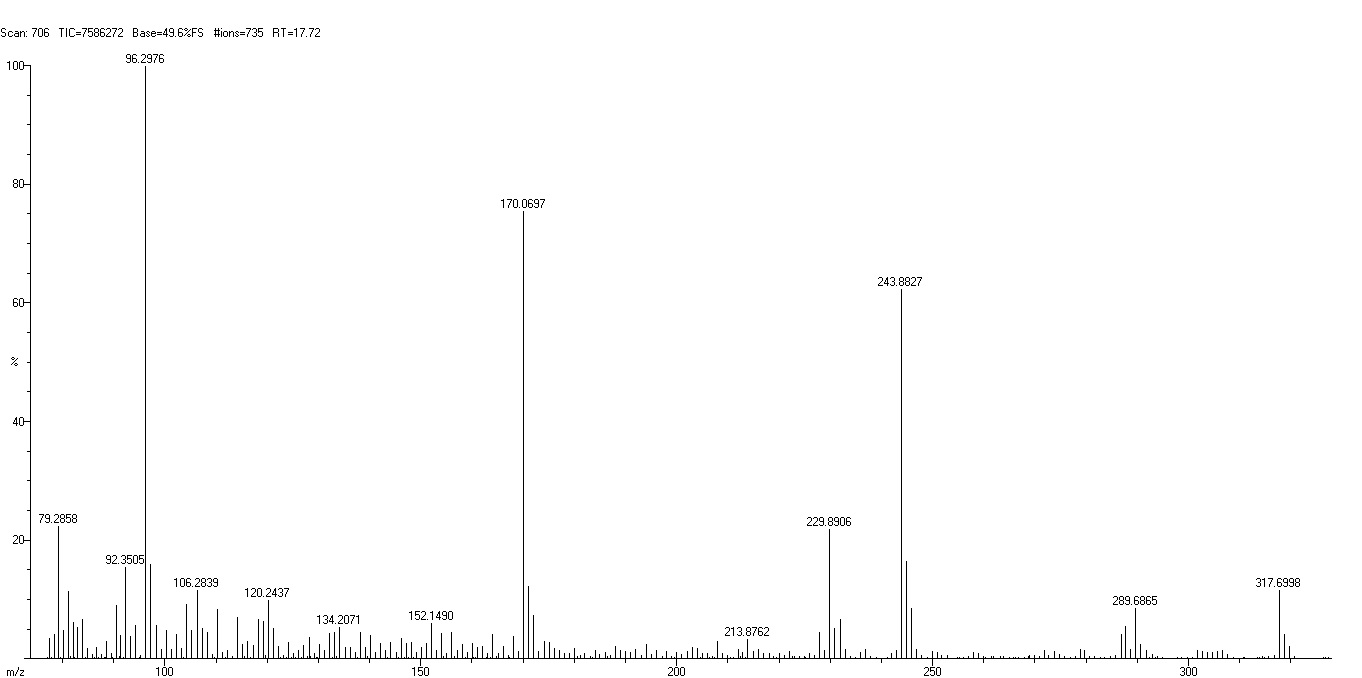


**Figure S30.** MASS of **IS10** compound.

**(Z)-4-bromo-N'-(5-fluoro-2-oxoindolin-3-ylidene)benzohydrazide (IS11) :** Yield 83%. NMR (400 MHz, ) δ 11.46 (s, 1H, NH), 7.88 – 7.86 (m, 2H, Ar-H), 7.68-7.65 (d, 2H, Ar-H), 7.54 (d, 1H, Ar-H), 7.43-7.39 (m,1H, Ar-H), 6.95 6.93 (m, 1H, Ar-H).  ^13^C NMR (400 MHz) δ: 163.40, 141.66, 133.56, 132.38, 131.76, 129.75, 128.02, 127.44, 121.02, 113.33. Molecular Formula: C_15_H_9_FBrN_3_O_2_ (ESI) Calculated= 362.1532, Observed= 362.1598.


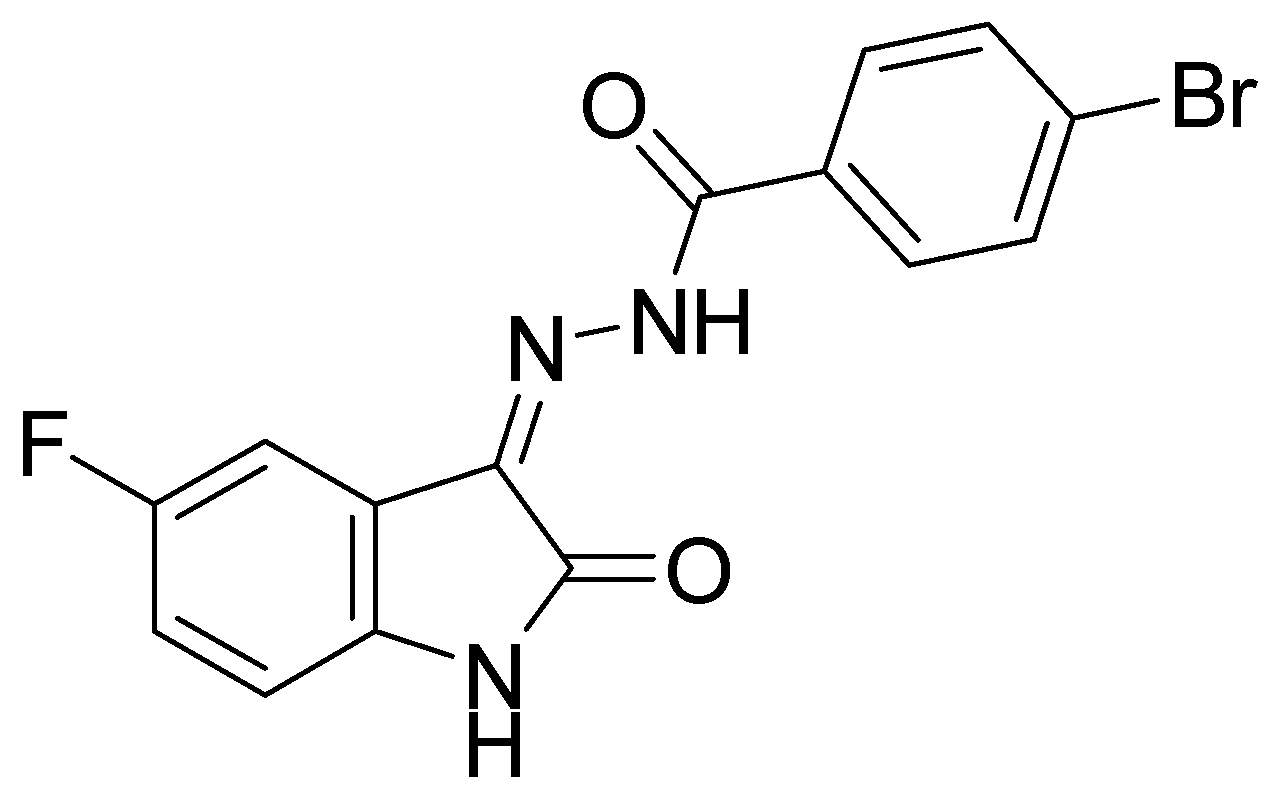


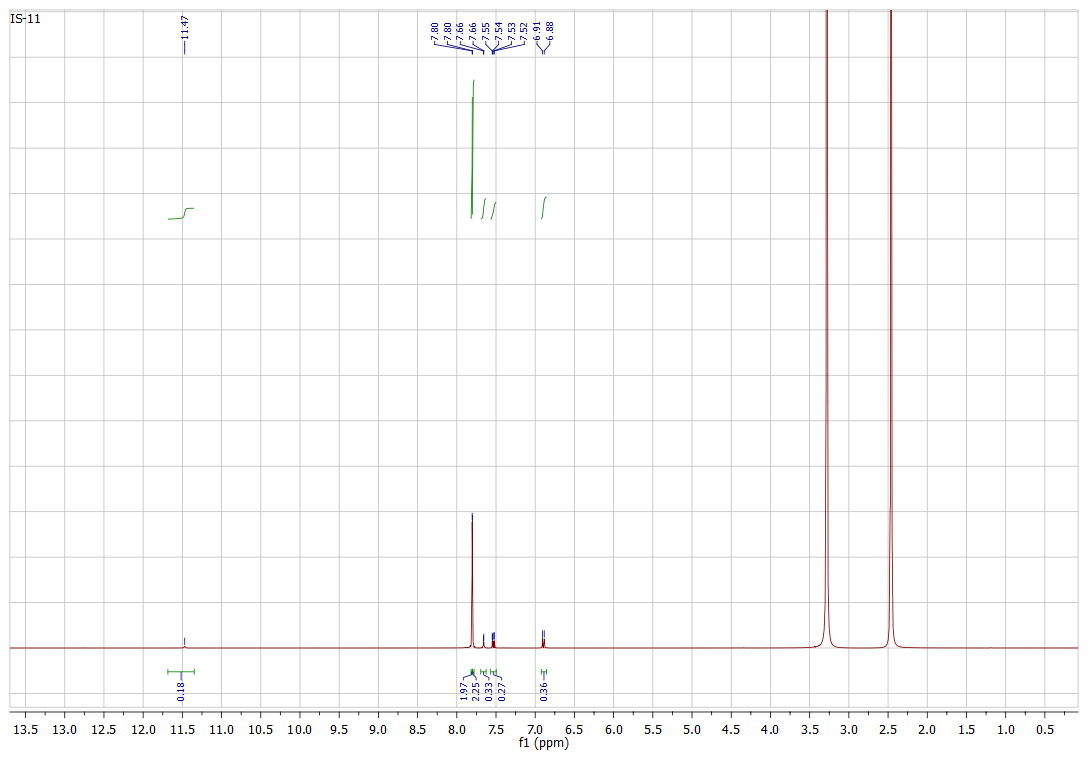


**Figure 31.** ^1^H NMR of **IS11** compound.


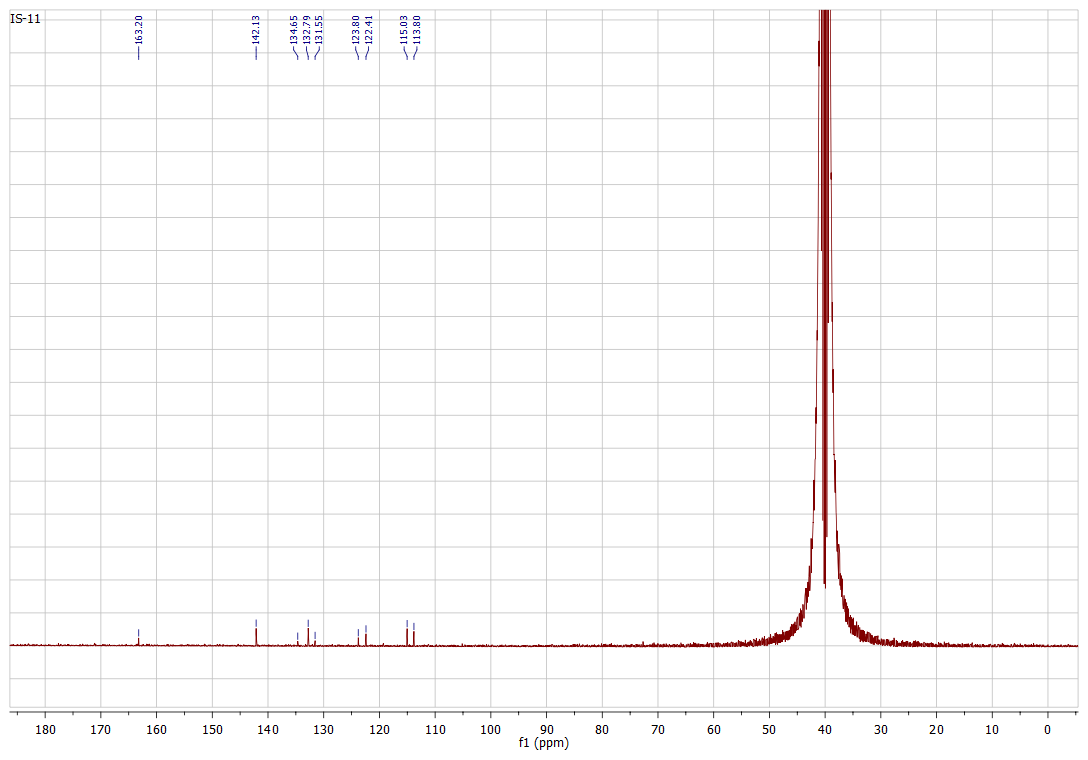


**Figure S32.** ^13^C NMR of **IS11** compound.


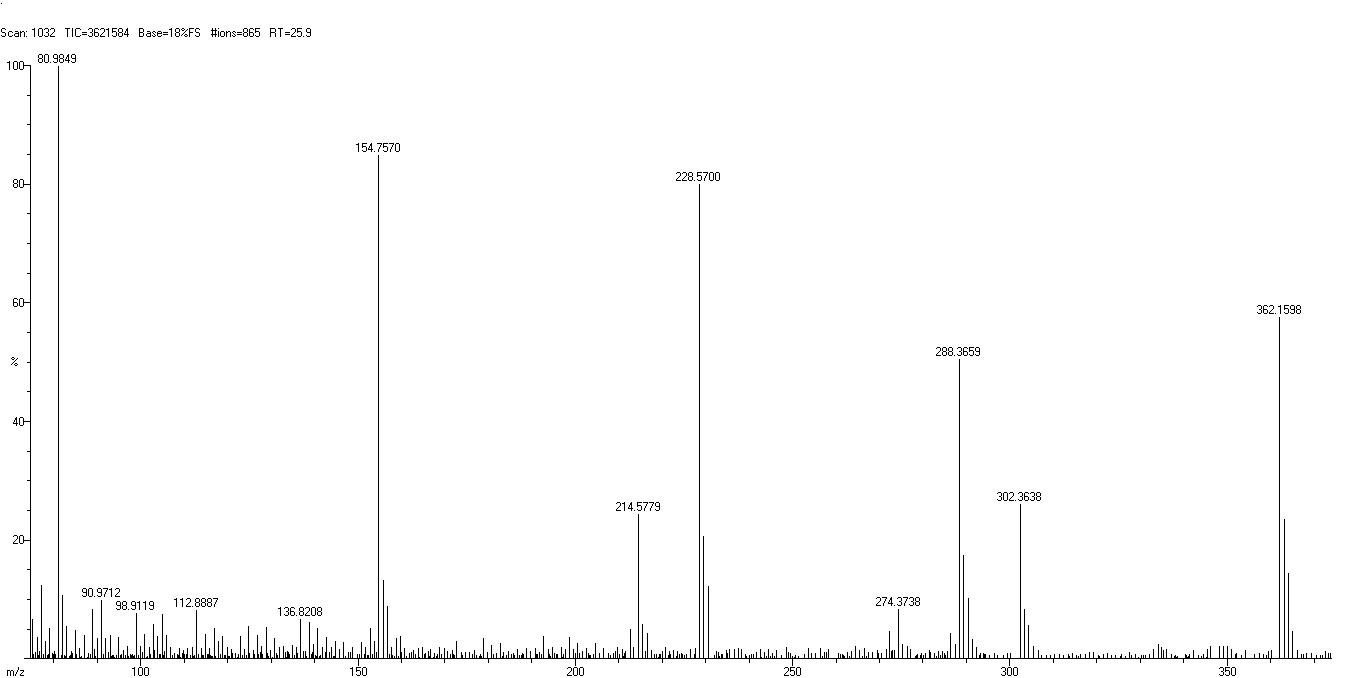


**Figure S33.** MASS of **IS11** compound.

**(Z)-4-fluoro-N'-(5-fluoro-2-oxoindolin-3-ylidene)benzohydrazide (IS12) :** Yield 76%. NMR (400 MHz) δ 12.76 (s, 1H), 11.48 (s, 1H), 7.3 – 7.86 (m, 4H), 7.71 – 7.67 (m, 1H), 7.44 – 7.40 (m, 1H), 6.99 – 6.95 (m, 1H). ). ^13^C NMR (400 MHz) δ 163.35, 141.74, 138.35, 131.85, 131.19, 129.84, 127.46, 122.00, 121.07, 113.35. Molecular Formula: C_15_H_9_F_2_N_3_O_2_ (ESI) Calculated= 301.2476, Observed= 301.2499.


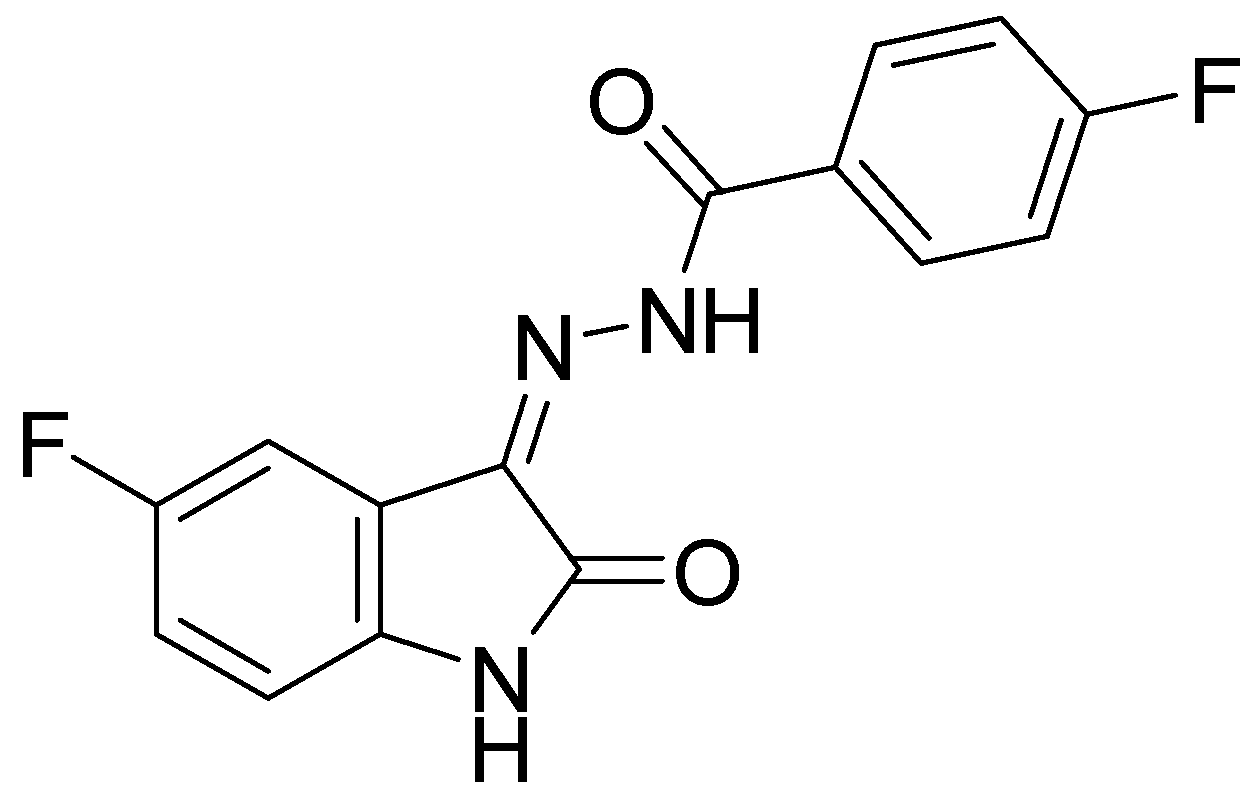


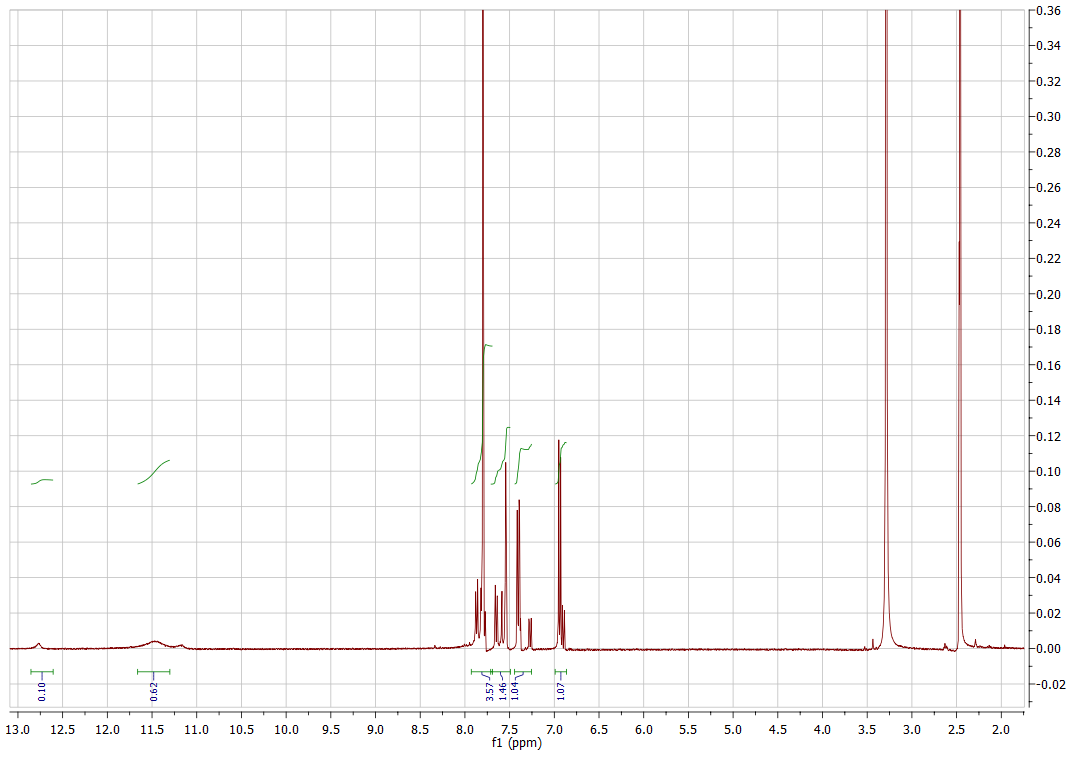


**Figure S34.** ^1^H NMR of **IS12** compound.


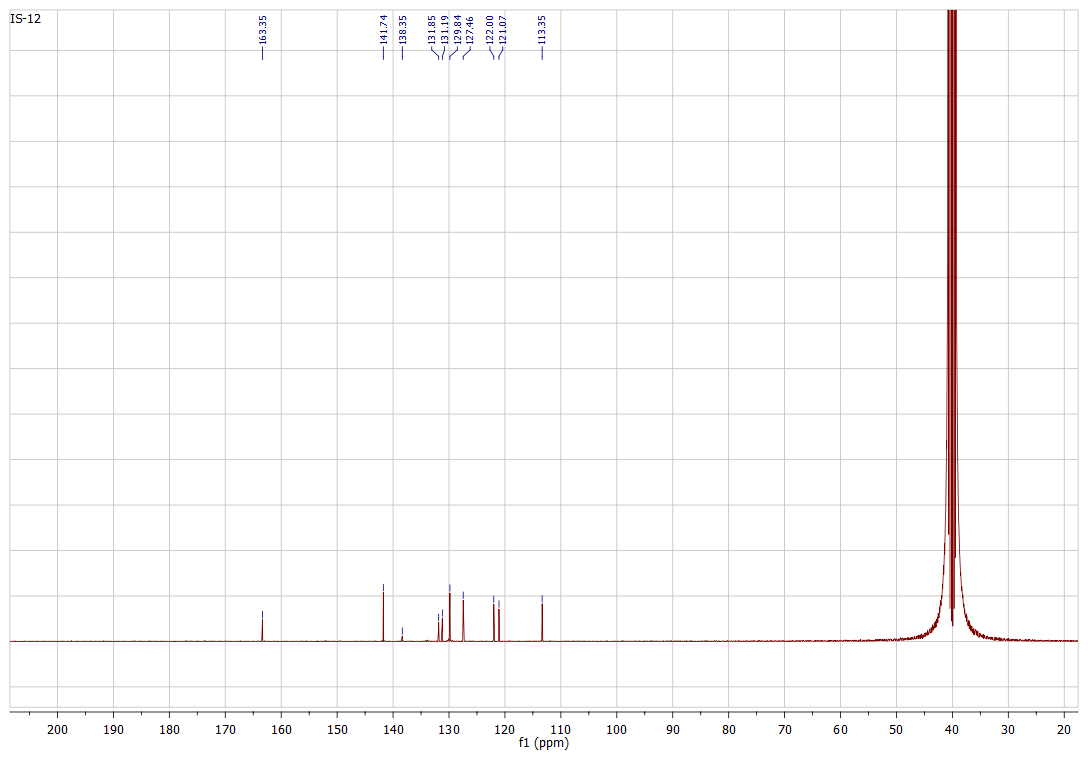


**Figure S35.** ^13^C NMR of **IS12** compound.


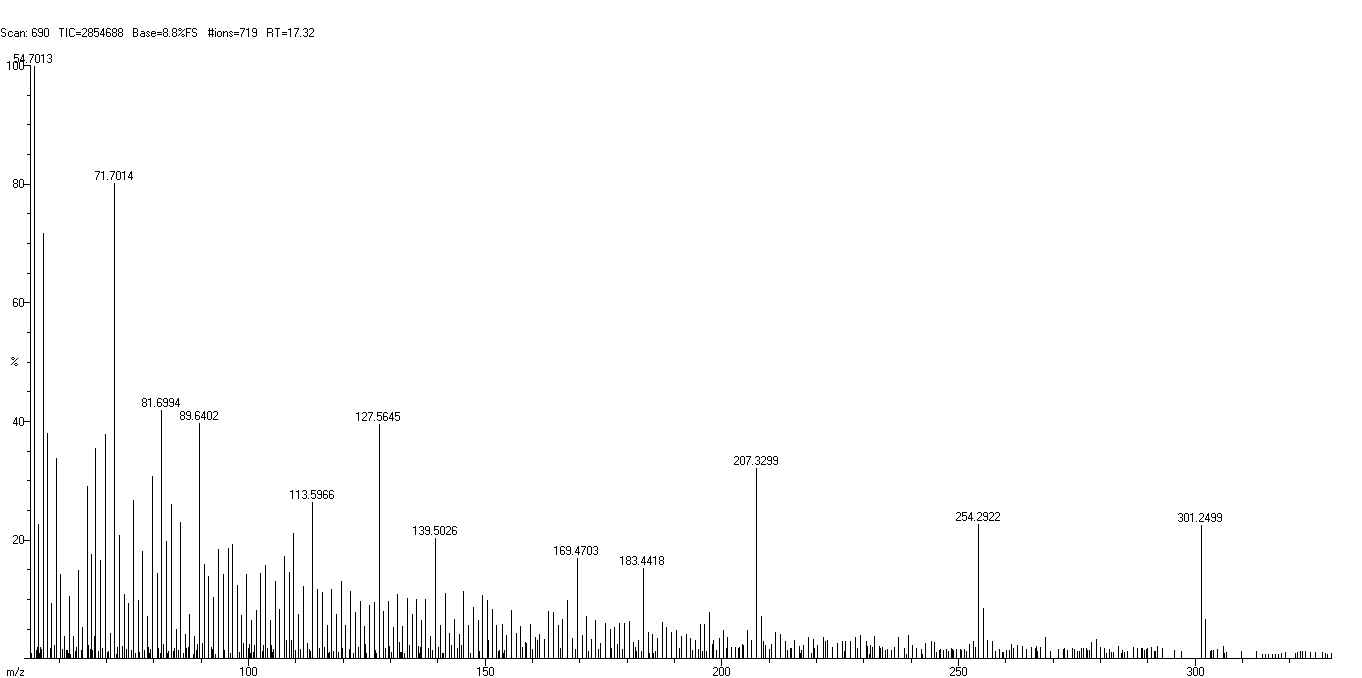


**Figure S36.** MASS of **IS12** compound.

**(Z)-N'-(5-chloro-2-oxoindolin-3-ylidene)benzohydrazide (IS13**): Yield 89%. NMR (400 MHz, ) δ 11.45 (s, 1H, NH), 7.86 (m, 3H, Ar-H), 7.65 (m, 1H, Ar-H), 7.57 (m, 2H, Ar-H), 7.39 (m, 1H, Ar-H), 6.94 (m, 1H, Ar-H). ^13^C NMR (400 MHz) δ: δ 163.40, 141.66, 133.56, 131.76, 129.75, 128.02, 121.02, 113.33, Molecular Formula: C_15_H_10_ClN_3_O_2_ (ESI) Calculated= 299.7118, Observed= 299.7098.


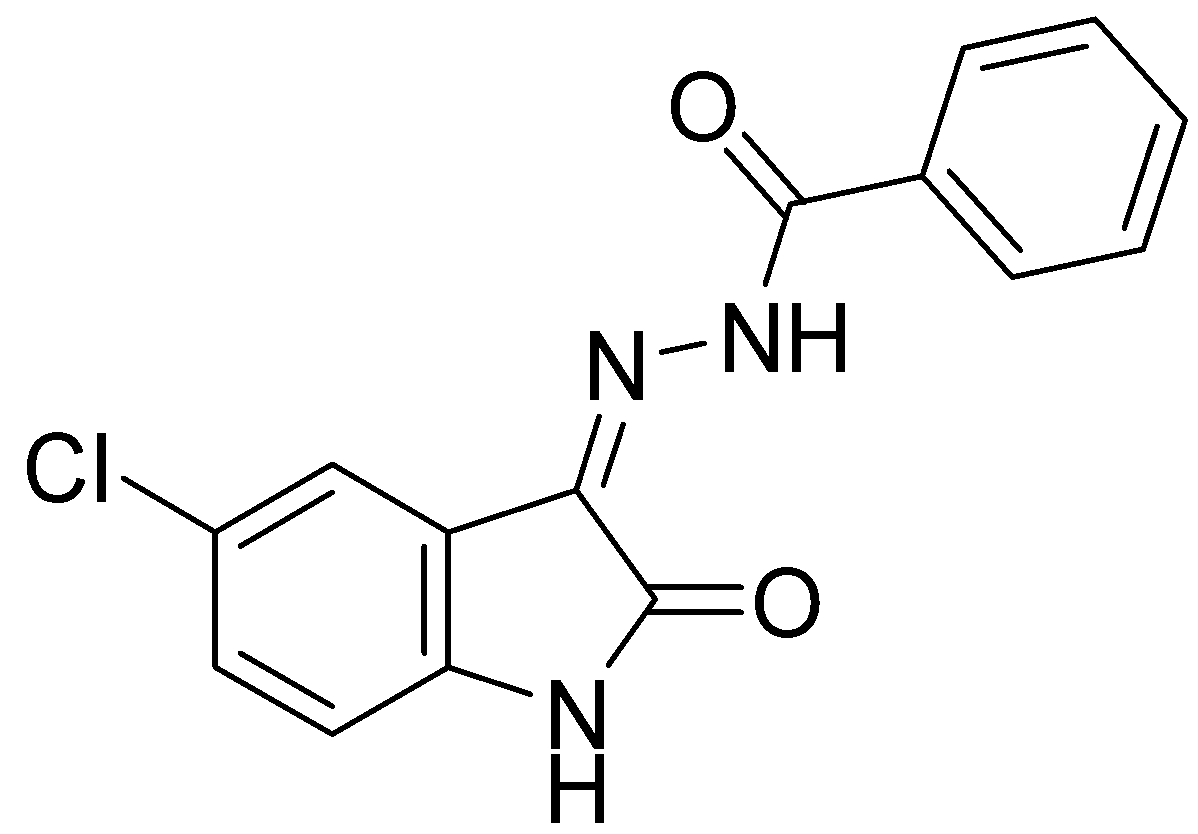


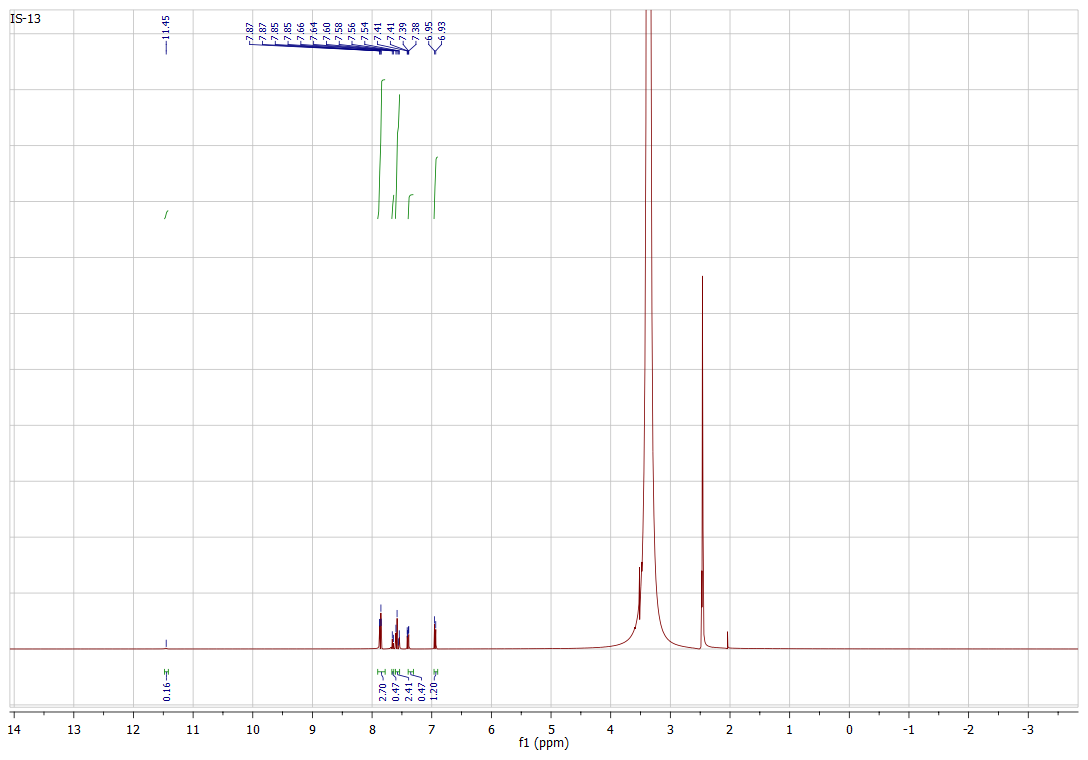
**Figure S37.** ^1^H NMR of **IS13** compound.


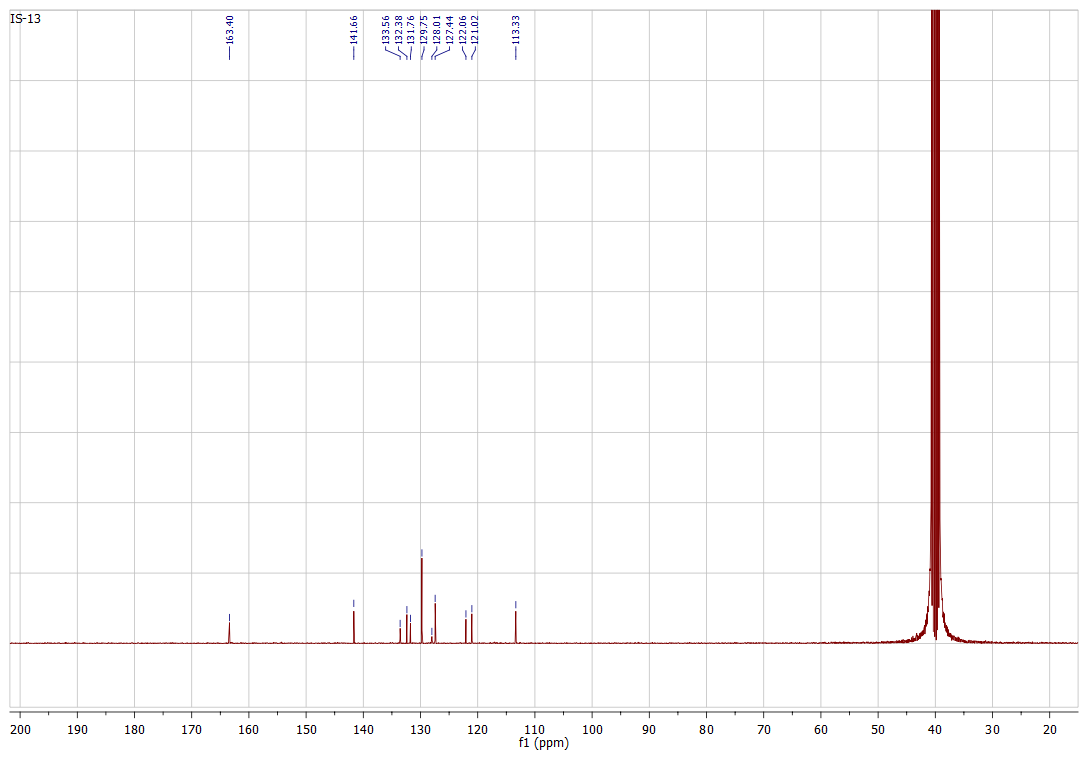


**Figure S38.** ^13^C NMR of **IS13** compound.


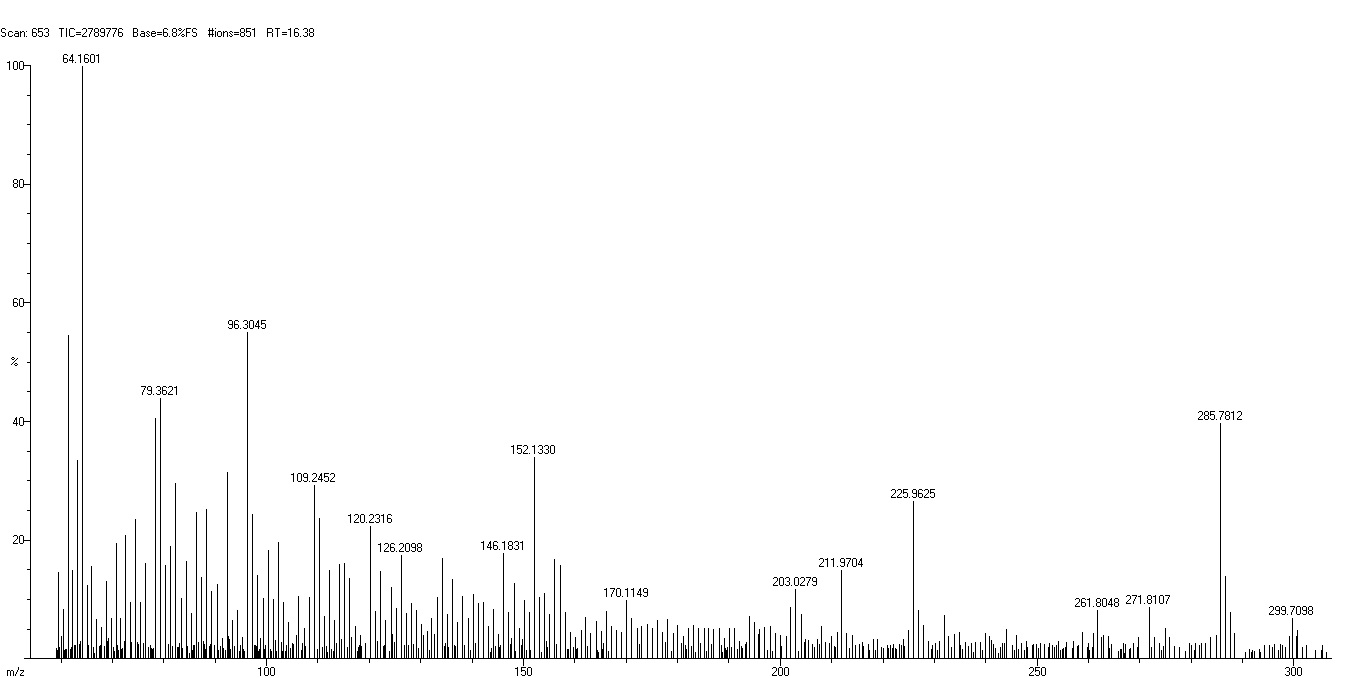


**Figure S39.** MASS of **IS13** compound.

***(Z)-4-chloro-N'-(5-chloro-2-oxoindolin-3-ylidene)benzohydrazide (IS14)***: Yield 86%. NMR (400 MHz, ) δ 11.46 (s, 1H, NH), 7.87 (m, 2H, Ar-H), 7.66 (m, 2H, Ar-H), 7.54 (m, 1H, Ar-H), 7.40 (m, 1H, Ar-H), 6.98 (m, 1H, Ar-H).  ^13^C NMR (400 MHz) δ: 163.35, 141.74, 138.35, 131.85, 131.19, 129.84, 127.46, 122.00, 121.07, 113.35. Molecular Formula: C_15_H_9_Cl_2_N_3_O_2_ (ESI) Calculated= 334.1568, Observed= 334.1598.


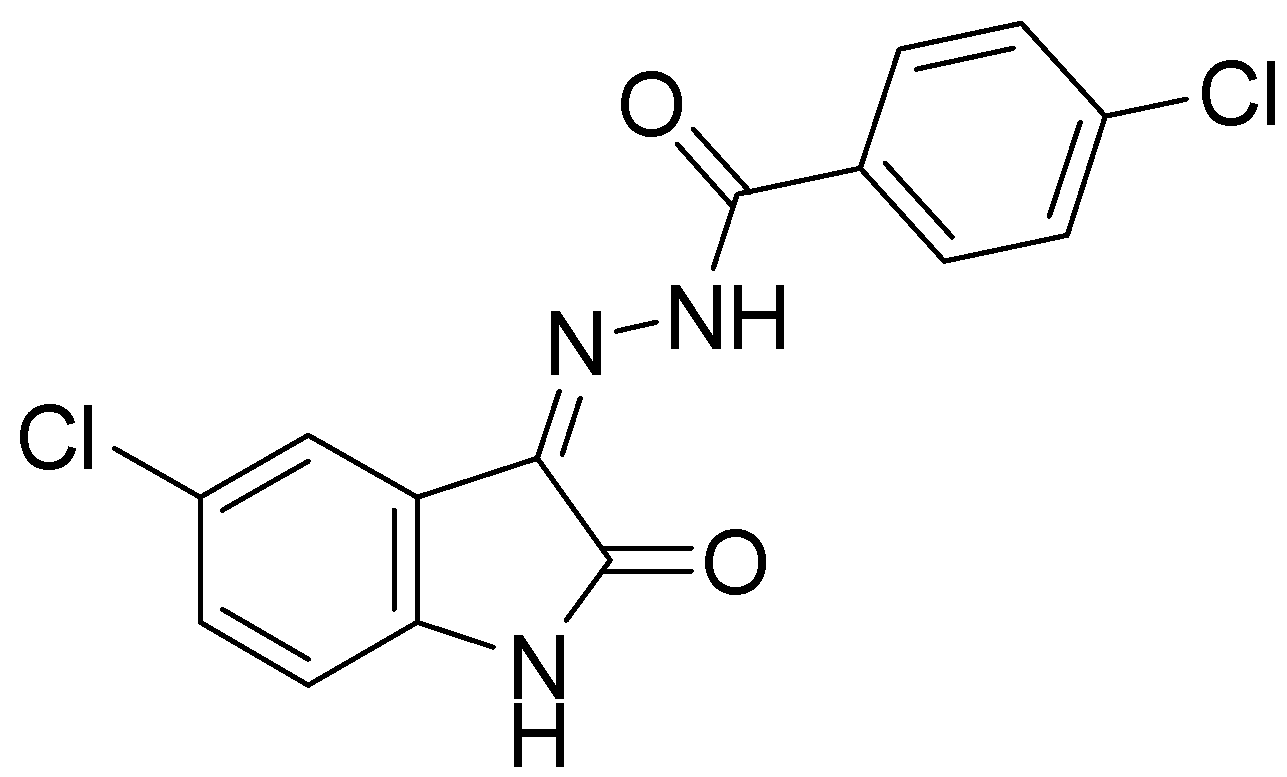


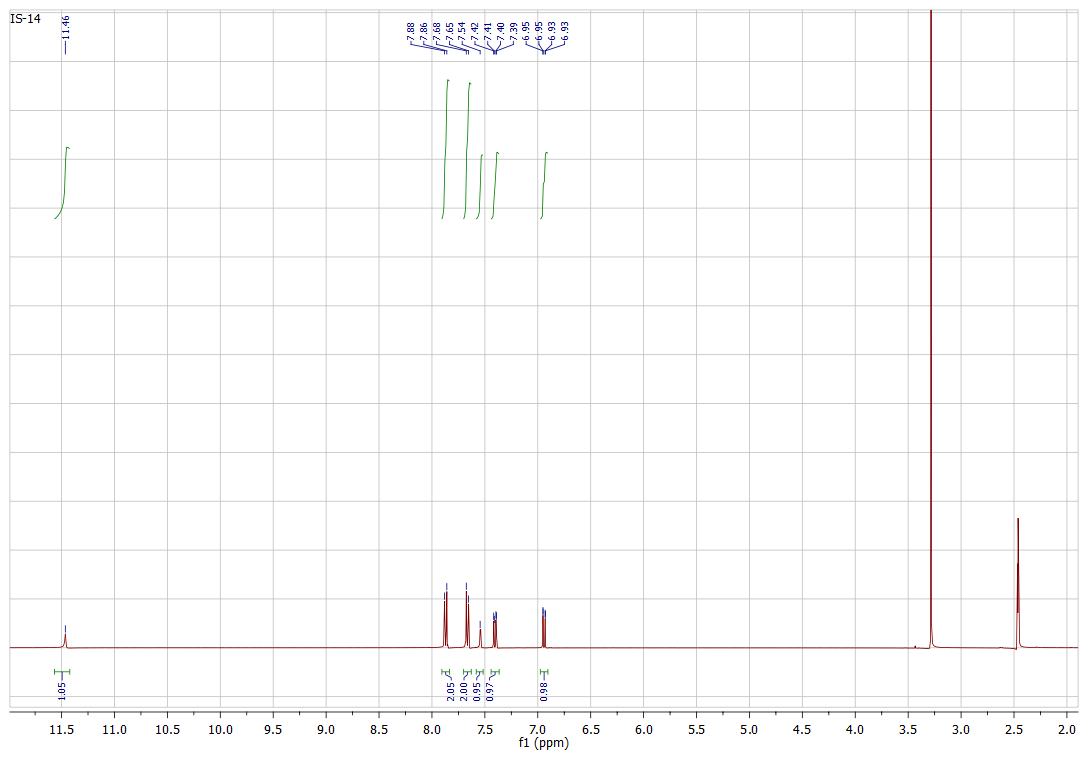


**Figure S40.** ^1^H NMR of **IS14** compound.
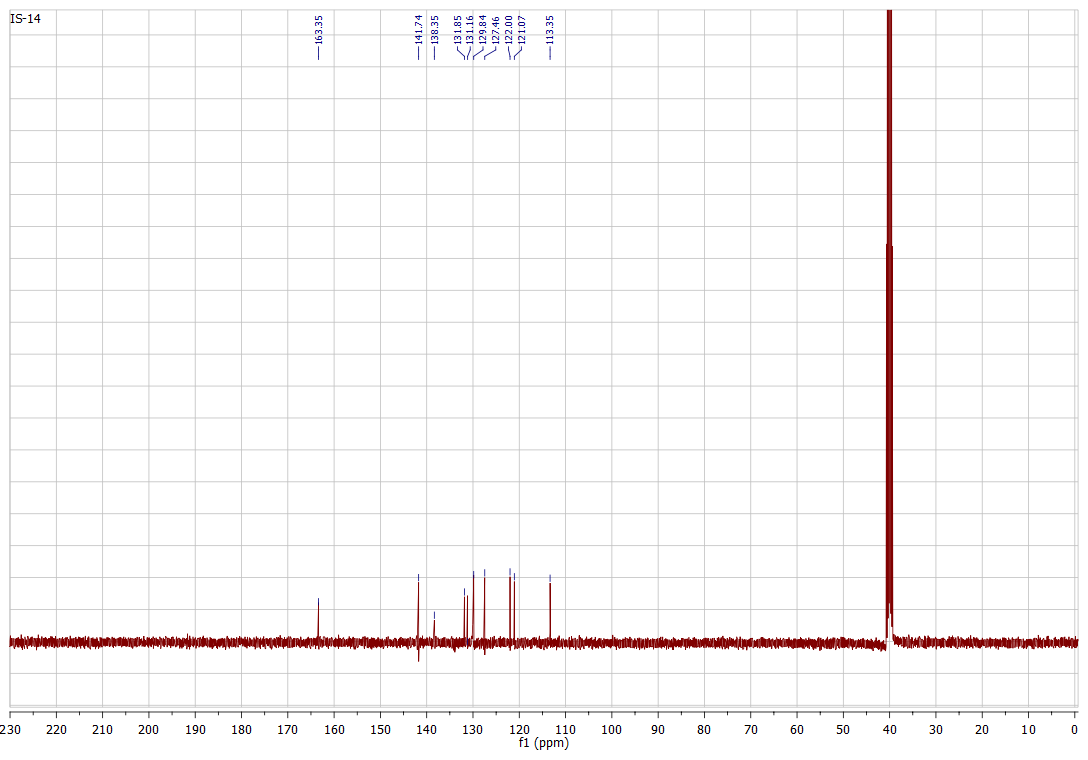


**Figure S41.** ^13^C NMR of **IS14** compound.


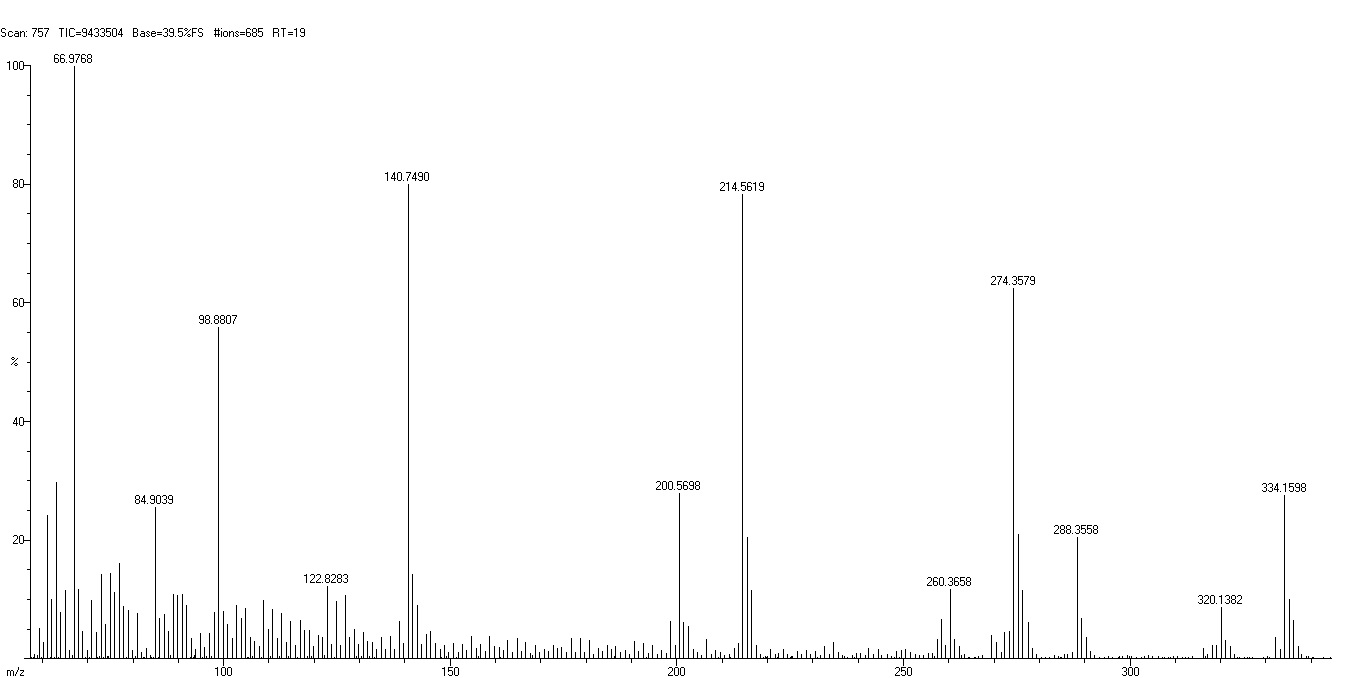


**Figure S42.** MASS of **IS14** compound.

***(Z)-4-bromo-N'-(5-chloro-2-oxoindolin-3-ylidene)benzohydrazide (IS15)***: Yield 95%. NMR (400 MHz, ) δ 11.48 (s, 1H, NH), 7.80 (m, 4H, Ar-H), 7.54 (m, 1H, Ar-H), 7.40 (m, 1H, Ar-H), 6.94 (m, 1H, Ar-H). ^13^C NMR (400 MHz) δ: 163.36, 141.77, 132.78, 131.85, 131.54, 127.45, 122.00, 121.06, 113.34. Molecular Formula: C_15_H_9_ClBrN_3_O_2_ (ESI) Calculated= 378.6078, Observed= 378.6099.


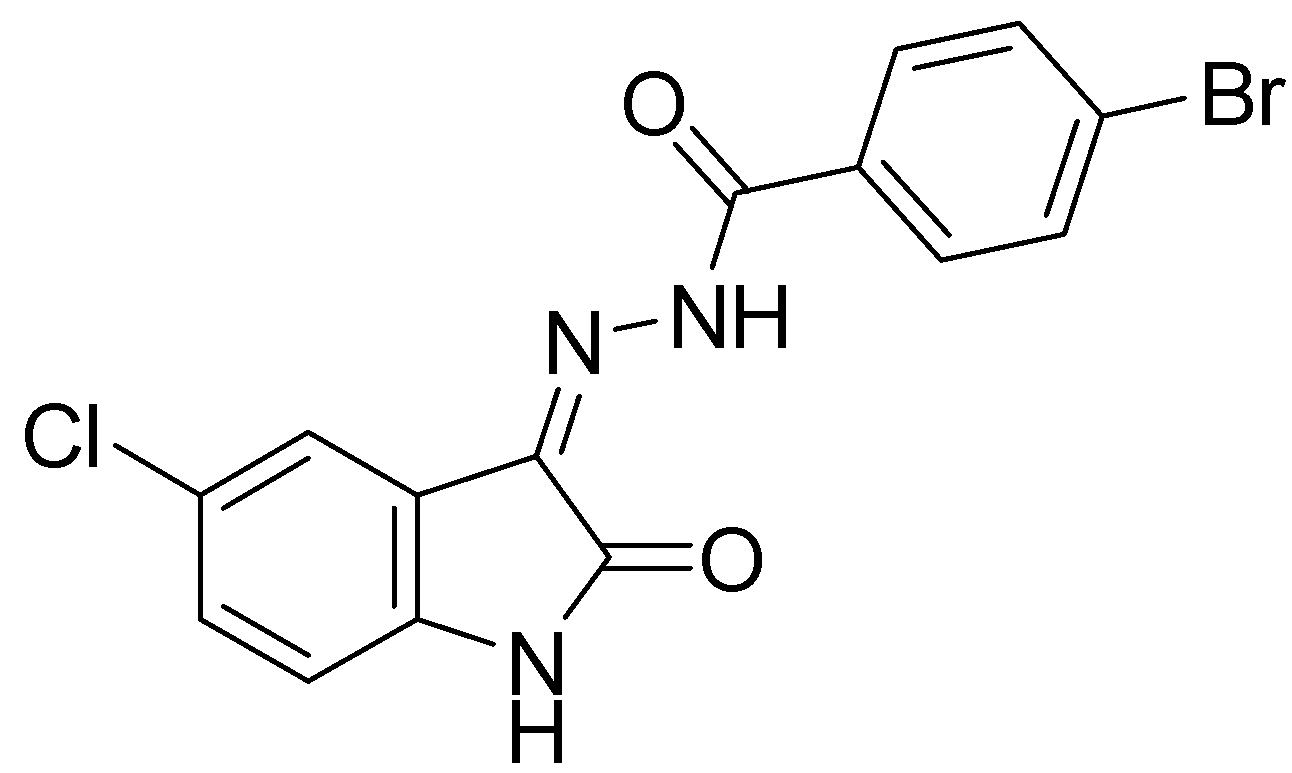


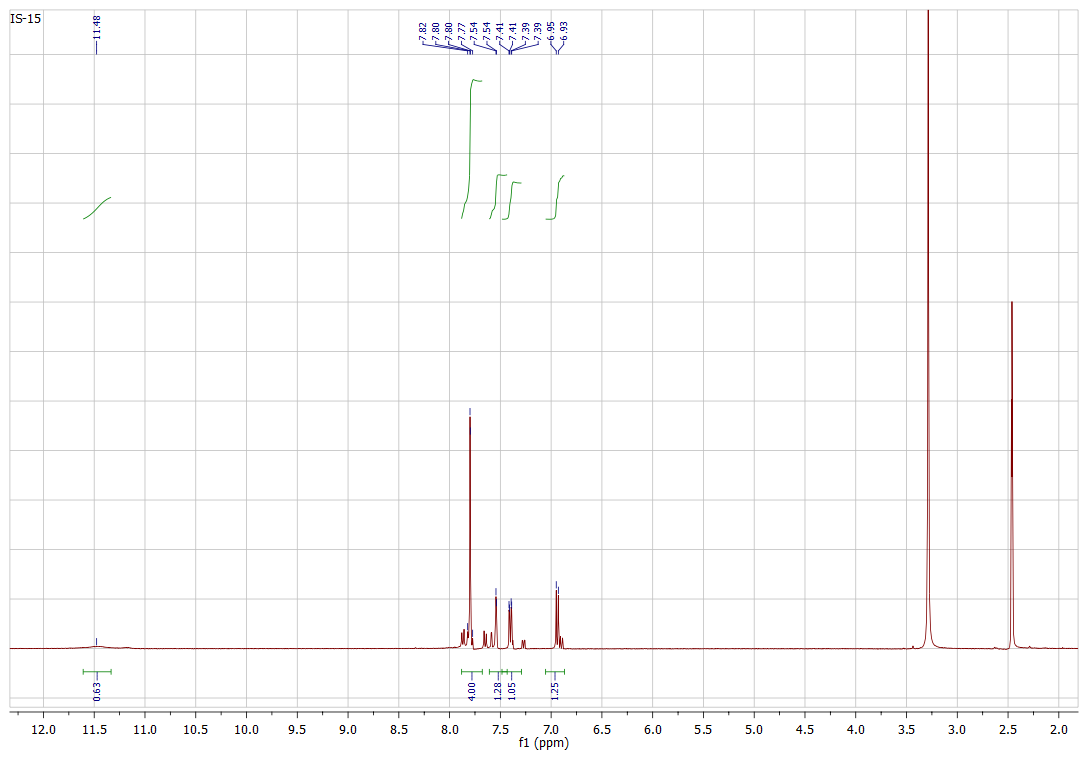


**Figure S43.** ^1^H NMR of **IS15** compound.


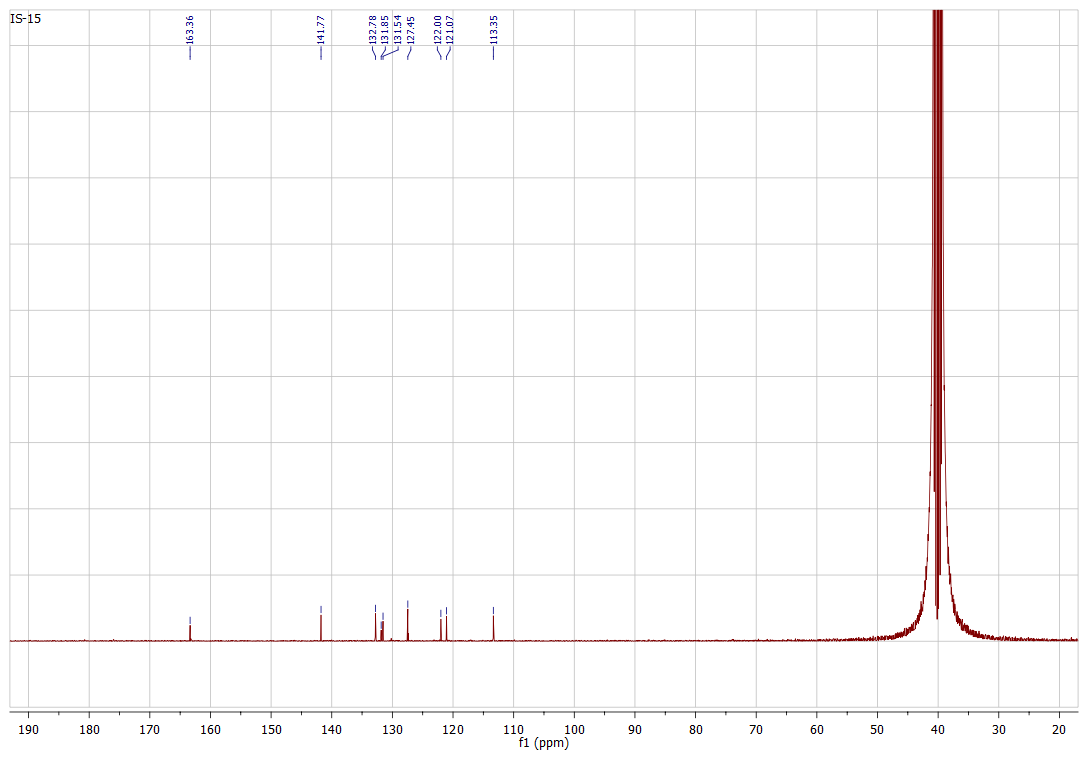


**Figure S44.** ^13^C NMR of **IS15** compound.


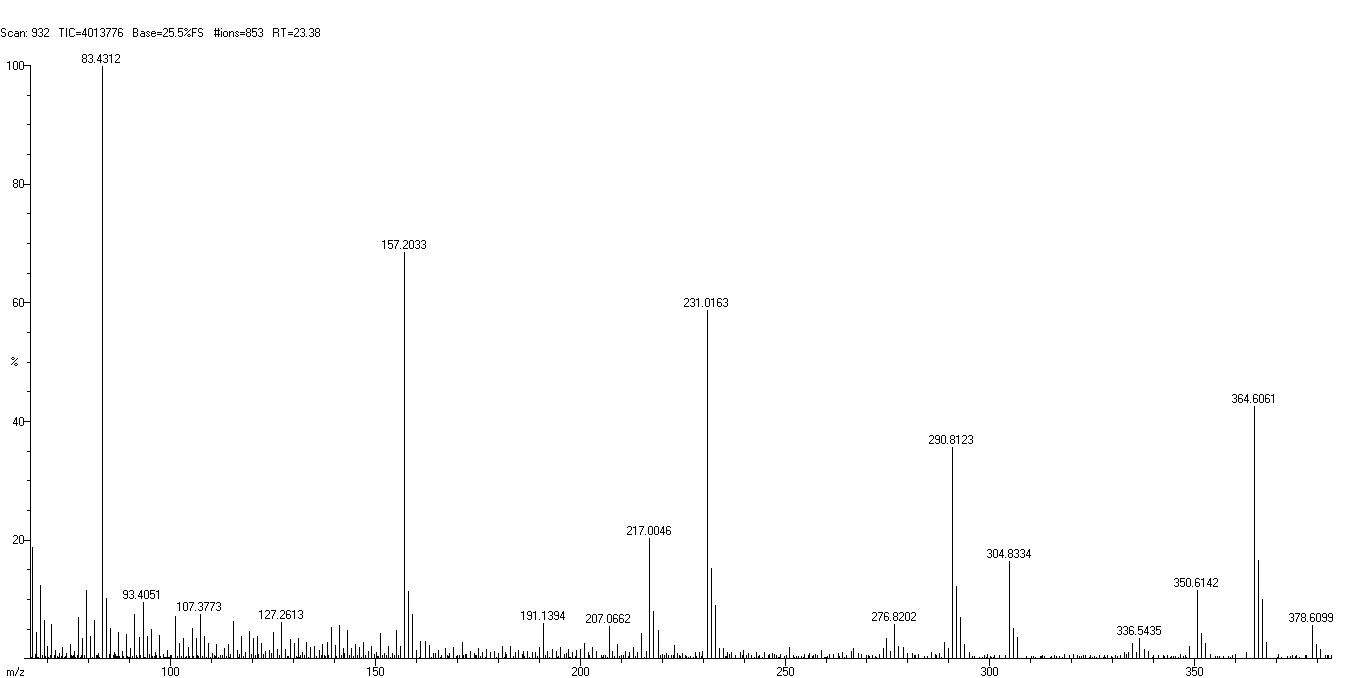


**Figure S45.** MASS of **IS15** compound.

***(Z)-N'-(5-chloro-2-oxoindolin-3-ylidene)-4-fluorobenzohydrazide (IS16)***: Yield 80%. NMR (400 MHz) δ 12.77 (s, 1H), 11.46 (s, 1H), 7.90-7.76 (m, 2H), 7.61-7.50 (m, 2H), 7.40 (m, 1H), 6.94 (d, 1H). ). ^13^C NMR (400 MHz) δ: 162.35, 141.75, 138.35, 131.85, 131.19, 130.06, 129.84, 127.46, 122.00, 121.07, 113.35. Molecular Formula: C15H9ClFN3O2 (ESI) Calculated= 317.7022, Observed= 317.6998.


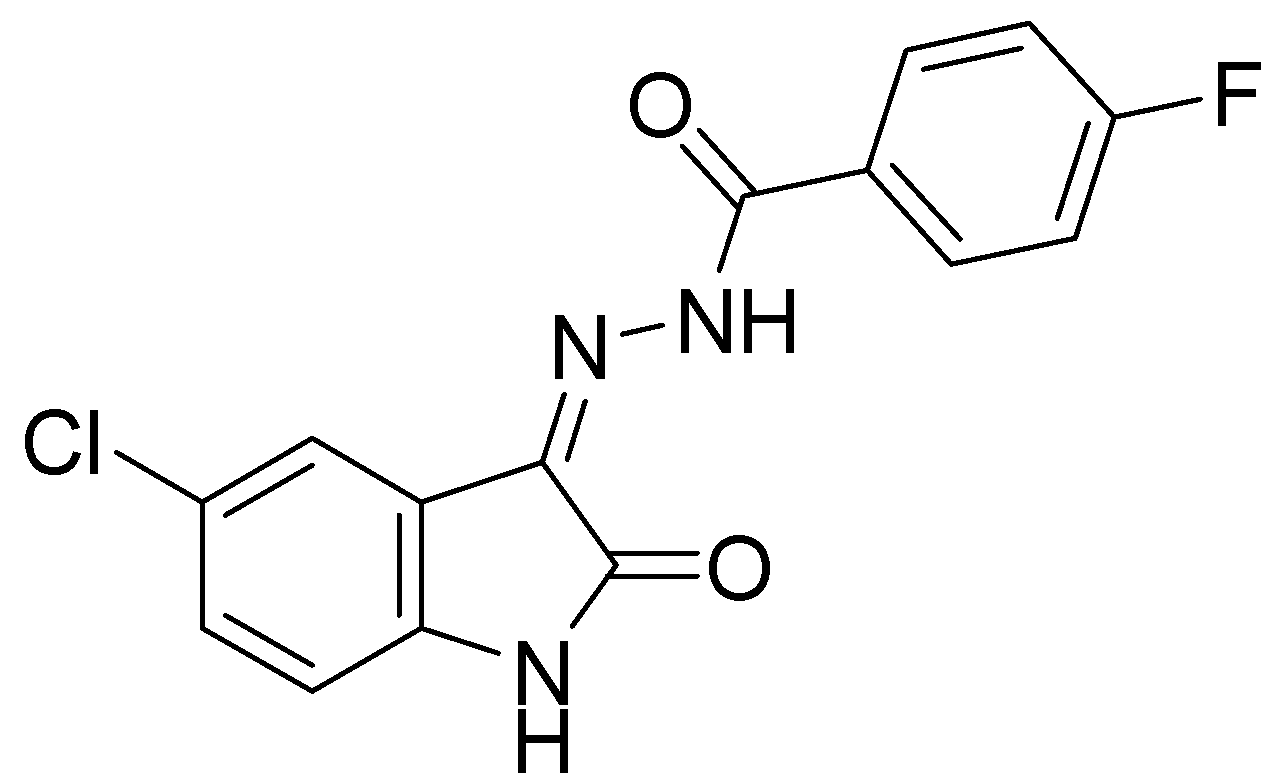


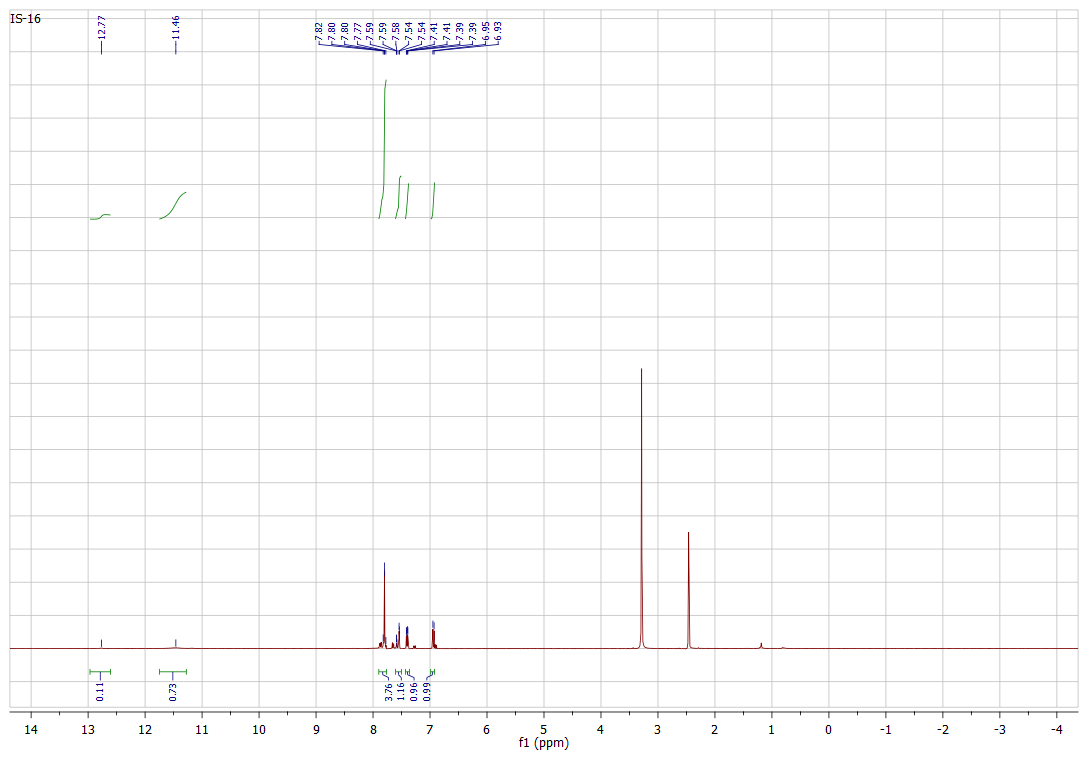


**Figure S46.** ^1^H NMR of **IS16** compound.
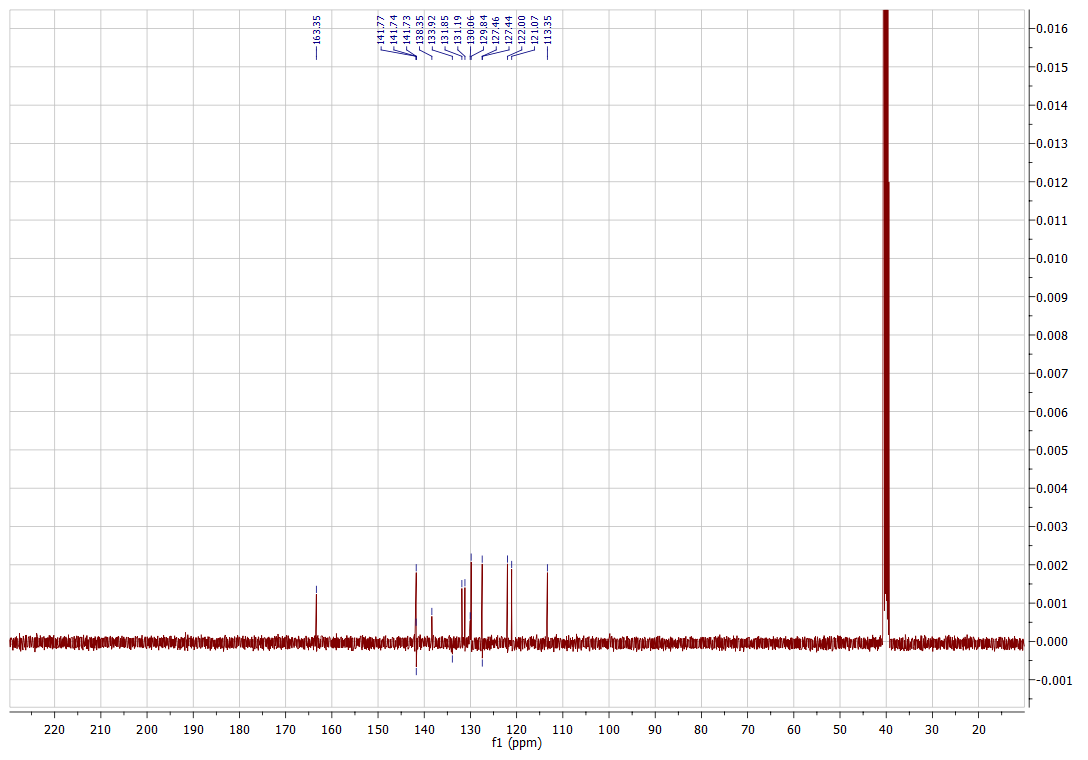


**Figure S47.** ^1^H NMR of **IS16** compound.


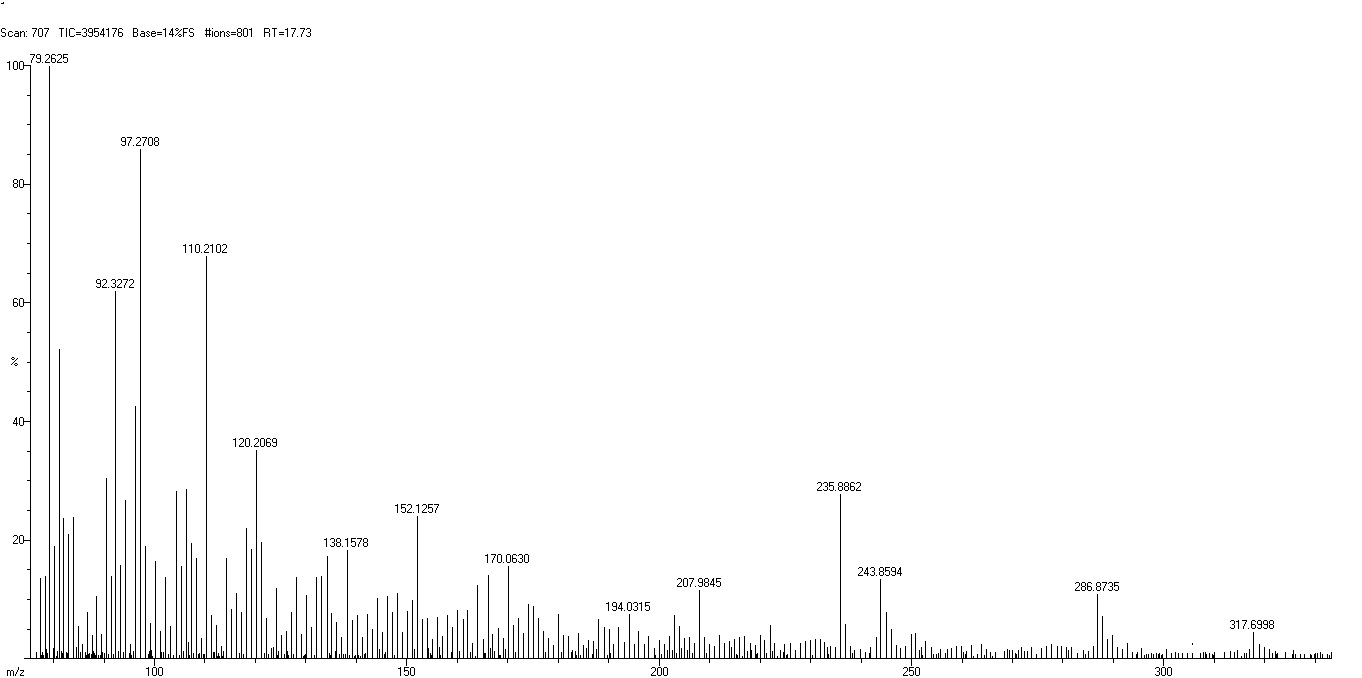


**Figure S48.** MASS of **IS16** compound.


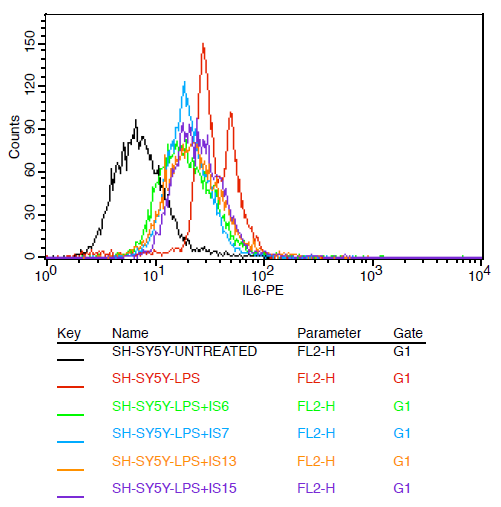


**Figure S49**. Overlaid IL-6 expression observed in LPS-induced SH-SY5Y cells in treated and untreated conditions.


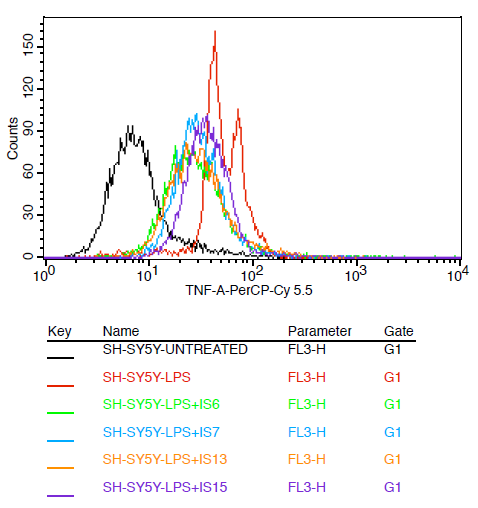


**Figure S49**. Overlaid TNF-alpha expression in LPS-induced SH-SY5Y cells in treated and untreated conditions


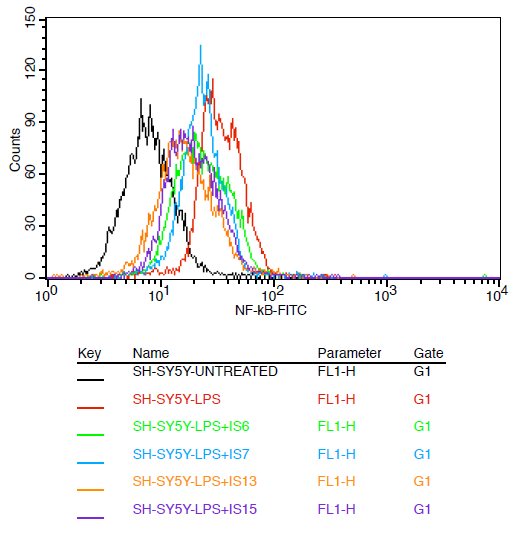


**Figure S50**. Overlaid NF-kB expression in LPS-induced SH-SY5Y cells in treated and untreated conditions


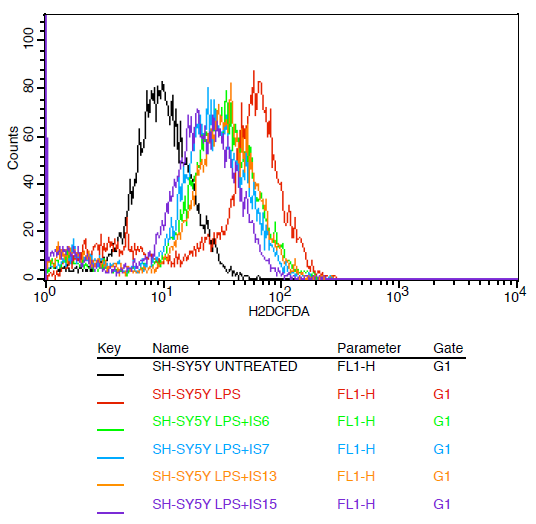


**Figure S51**. Overlaid histograms representing the DCF expression in Untreated, LPS induced alone and LPS stimulation followed by the treatment of **IS6**, **IS7**, **IS13**, and **IS15** with 10 µM concentration on Human Bone marrow neuroblastoma cells.

**(A) (B)**


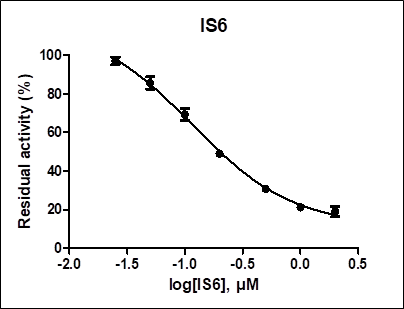

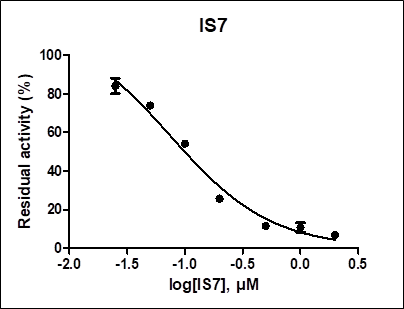


**(C)**


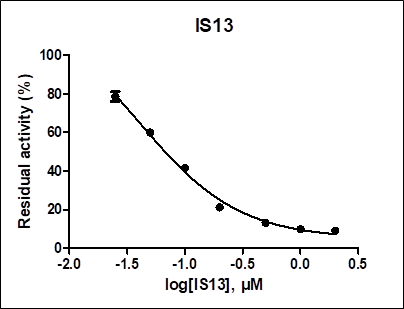


**Figure S52**. The IC_50_ curves of **IS6** (**A**), **IS7** (**B**), and **IS13** (**C**) for MAO-B.


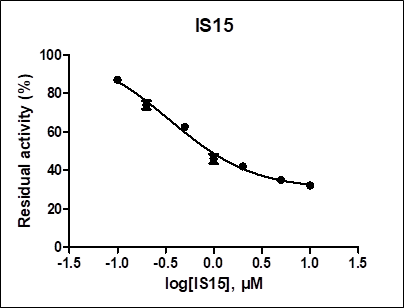


**Figure S53**. The IC_50_ curve of **IS15** for MAO-A.

**PAMPA assay**

Using a microtiter plate with 96 wells and a Millipore filter plate with 96 wells (ipvh, 125 μm thick filter, 0.45 μm pore), which was then saturated with 0.1 mL of n-dodecane, a "sandwich" structure was created in PAMPA. Stock solutions of drug samples in DMSO were created at a 10 mM concentration. The stock solution was diluted in buffer at pH 7.4 before being added to a 96-well filter plate in order to achieve a final sample concentration (0.01, 0.1, and 1 mM) and keep the DMSO concentration at 1% (v/v). The final dilutions were divided into 270 μL for the donor wells and 200 μL of pH 7.4 buffer for the acceptor well. The donor plate was placed on the top of the acceptor filter plate to form a sandwich (comprising of a synthetic lipid membrane in the center, an aqueous receiver on atop, and an aqueous donor carrying an analyte on the bottom.). The test substance diffuses from the donor well across the lipid membrane and into the acceptor well. The sandwich is said to have been intact when the penetration occurred. The drug concentration in the reference, donor, and receiver wells was measured using UV spectroscopy. The rate of penetration is calculated using the following expression.

**Log *Pe* = -ln [1-C_A_ /E*_quilibrium_*]/A× (1/V_D_ + 1/V_A_) × t**

Where, *Pe* is permeability (cm/s)

C_A_ = receptor concentration

A = area of effective filtration (0.3 cm^2^)

V_D_ = donor volume (mL)

V_A_ = acceptor volume (mL)

t = time of incubation (s)

E*_quilibrium_=* (C_D_×V_D_ + C_A_×V_A_)/(V_D_+V_A_)

**Abbreviations**

MTT: (3-[4,5-dimethylthiazol-2-yl]-2,5 diphenyl tetrazolium bromide)

ROS: Reactive oxygen species

SOD: Superoxide dismutase

CAT: Catalase

GSH: Glutathione

GPx: Glutathione peroxidase

IL-6: Interleukin-6

TNF-alpha: Tumor necrosis factor-αlpha

NF-kB: Nuclear factor kappa B

LPS: lipopolysaccharide

CNS: central nervous system

MAO: monoamine oxidase

PAMPA: Parallel artificial membrane permeability assay

AD: Alzheimer’s disease

PD: Parkinson’s disease

NA: noradrenaline

DA: dopamine

SNpc: substantia nigra pars compacta

TLC: thin layer chromatography
